# The dominant *Anopheles *vectors of human malaria in Africa, Europe and the Middle East: occurrence data, distribution maps and bionomic précis

**DOI:** 10.1186/1756-3305-3-117

**Published:** 2010-12-03

**Authors:** Marianne E Sinka, Michael J Bangs, Sylvie Manguin, Maureen Coetzee, Charles M Mbogo, Janet Hemingway, Anand P Patil, Will H Temperley, Peter W Gething, Caroline W Kabaria, Robi M Okara, Thomas Van Boeckel, H Charles J Godfray, Ralph E Harbach, Simon I Hay

**Affiliations:** 1 Spatial Ecology and Epidemiology Group, Tinbergen Building, Department of Zoology, University of Oxford, South Parks Road, Oxford OX1 3PS, UK; 2 Public Health and Malaria Control Department, PT Freeport Indonesia, Kuala Kencana, Papua, Indonesia; 3 Institut de Recherche pour le Développement, Lab. d'Immuno-Physiopathologie Moléculaire Comparée, UMR-MD3/Univ. Montpellier I, Faculté de Pharmacie, 15, Ave Charles Flahault, 34093 Montpellier, France; 4 Malaria Entomology Research Unit, School of Pathology, Faculty of Health Sciences, University of the Witwatersrand, Johannesburg, South Africa; 5 Vector Control Reference Unit, National Institute for Communicable Diseases of the National Health Laboratory Service, Private Bag X4, Sandringham 2131, Johannesburg, South Africa; 6 KEMRI/Wellcome Trust Programme, Centre for Geographic Medicine Research - Coast, Kilifi, Kenya; 7 Liverpool School of Tropical Medicine, Liverpool, UK; 8 Malaria Public Health and Epidemiology Group, Centre for Geographic Medicine, KEMRI - Univ. Oxford - Wellcome Trust Collaborative Programme, Kenyatta National Hospital Grounds, P.O. Box 43640-00100 Nairobi, Kenya; 9 Biological Control and Spatial Ecology, Université Libre de Bruxelles CP160/12, Av FD Roosevelt 50, B1050, Brussels, Belgium; 10 Department of Entomology, The Natural History Museum, Cromwell Road, London, SW7 5BD, UK

## Abstract

**Background:**

This is the second in a series of three articles documenting the geographical distribution of 41 dominant vector species (DVS) of human malaria. The first paper addressed the DVS of the Americas and the third will consider those of the Asian Pacific Region. Here, the DVS of Africa, Europe and the Middle East are discussed. The continent of Africa experiences the bulk of the global malaria burden due in part to the presence of the *An. gambiae *complex. *Anopheles gambiae *is one of four DVS within the *An. gambiae *complex, the others being *An. arabiensis *and the coastal *An. merus *and *An. melas*. There are a further three, highly anthropophilic DVS in Africa, *An. funestus*, *An. moucheti *and *An. nili*. Conversely, across Europe and the Middle East, malaria transmission is low and frequently absent, despite the presence of six DVS. To help control malaria in Africa and the Middle East, or to identify the risk of its re-emergence in Europe, the contemporary distribution and bionomics of the relevant DVS are needed.

**Results:**

A contemporary database of occurrence data, compiled from the formal literature and other relevant resources, resulted in the collation of information for seven DVS from 44 countries in Africa containing 4234 geo-referenced, independent sites. In Europe and the Middle East, six DVS were identified from 2784 geo-referenced sites across 49 countries. These occurrence data were combined with expert opinion ranges and a suite of environmental and climatic variables of relevance to anopheline ecology to produce predictive distribution maps using the Boosted Regression Tree (BRT) method.

**Conclusions:**

The predicted geographic extent for the following DVS (or species/suspected species complex*) is provided for Africa: *Anopheles *(*Cellia*) *arabiensis*, *An. *(*Cel.*) funestus*, *An. *(*Cel.*) *gambiae*, *An. *(*Cel.*) *melas*, *An. *(*Cel.*) *merus*, *An. *(*Cel.*) *moucheti *and *An. *(*Cel.*) nili*, and in the European and Middle Eastern Region: *An. *(*Anopheles*) *atroparvus*, *An. *(*Ano.*) *labranchiae*, *An. *(*Ano.*) *messeae*, *An. *(*Ano.*) *sacharovi*, *An. *(*Cel.*) *sergentii *and *An. *(*Cel.*) superpictus*. These maps are presented alongside a bionomics summary for each species relevant to its control.

## Background

This paper is a second in a series of three contributions discussing the geographic distribution and bionomics of the dominant vector species (DVS) of human malaria [1,2]. It deals specifically with the DVS of Africa, Europe and the Middle East.

Despite highly variable levels of transmission across Africa [3,4], the global public heath impact of *P. falciparum *malaria is overwhelmingly felt on this continent [5,6]. Africa contains areas with the highest entomological inoculation rates [3,7] and prevalence levels [8] globally, and thus the highest morbidity and mortality [5]. This situation arises partly because Africa has the most effective and efficient DVS of human malaria [9,10]: *An. gambiae *(*sensu stricto *- herein, referred to as '*An. gambiae*'; it is not necessary to use '*sensu stricto*' (or the abbreviation '*s.s.*') when there is no doubt that the biological species being referred to is the one that bears the name *An. gambiae*) [5,10], with its sibling, *An. arabiensis*, also of major importance [11]. The DVS members of the *An. gambiae *complex also include the salt water tolerant, coastal species *An. melas *and *An. merus *[12] and these, whilst not being as efficient at transmitting malaria as *An. gambiae *or *An. arabiensis*, are often found in such high densities that they achieve DVS status [13-15]. Other members of the *An. gambiae *complex are either highly restricted in their distribution (*e.g*. *An. bwambae*, only currently known to occur in geothermal springs in western Uganda [11,16]) or are zoophilic in behaviour and not considered vectors of human malaria (*e.g. An. quadriannulatus *and *An. quadriannulatus *B) [17]. In addition to the four DVS within the *An. gambiae *complex, large parts of Africa are also home to other DVS, including *An. funestus*, *An. nili *and *An. moucheti*, with *An. funestus*, in some cases, having a greater impact on malaria transmission even than *An. gambiae *[10,11,18]. The anthropophilic habits of these DVS are a major contributing factor to their public health impact, indeed *An. funestus *is considered to be one of the first species to have adapted to human hosts [19].

The vast majority of current malaria control efforts use interventions aimed at limiting human-vector contact [20,21]. Foremost among these interventions has been the rapid scale-up of insecticide treated bednets (ITNs) [22], followed by the scale-up of indoor residual spraying (IRS) in Africa [23]. These interventions are often deployed without a detailed understanding of the distribution, species composition and behaviour of local vectors. This complicates impact monitoring [24], the appraisal of arguments for more holistic integrated vector control [25] and evaluation of the potential of novel vector control methods [26-28]. Distribution maps can also be applied to gauge the importance of emerging insecticide resistance among the DVS of Africa [29-37]. In contrast to Africa, the European and the Middle Eastern region contain areas with low to no malaria transmission [8]. Despite this, the existence of *Anopheles *species with the capacity to transmit malaria is often highlighted as providing the potential for the re-introduction of malaria [38-43].

A number of vector species modelling and mapping strategies have been applied on a country (*e.g. *[44-50]) and regional scale [51] and across the African continent [24,52-55], with fewer attempts directed at the European and Middle Eastern species [56-58]. No previous mapping efforts formally incorporate expert opinion (EO) distributions and the methods used range in complexity, from simply plotting presence or abundance on a map [24,44,48,57,58], to the application of more sophisticated predictive models [45-47,49,50,52-56]. This makes comparison between the maps difficult. Further difficulties also arise in the interpretation of existing maps as many previous studies include all historical occurrence records to compensate for poor data coverage. This can introduce taxonomic ambiguity; the *An. gambiae *complex, for example, was only fully categorised in 1998, with the addition of the provisionally designated *An. quadriannulatus *species B [12,59] and, even now, the status of *An. funestus *is under question [60-63]. Moreover, the morphological similarity that hides members of a species complex adds a level of uncertainty to the identity of species data recorded before the advent of cytological or molecular identification techniques.

This current work attempts to overcome many of these problems. The same Boosted Regression Tree (BRT) methodology is applied to all DVS making comparison between predicted maps possible. Despite only using data collected after 31 December 1984 the assimilated DVS occurrence records together comprise the largest contemporary dataset for prediction, with this evidence base to be made available in the public domain. Significant efforts were also expended to update the EO maps for all species [1] and these were used to inform the predictions. The outcome of these efforts and that of a comprehensive bionomics review are presented here for the DVS of Africa, Europe and the Middle East.

## Methods

The data assembly and mapping methods, climatic and environmental variable grid pre- and post-processing methods and the modelling protocol summarised here are described in detail in Sinka *et al. *[2]. The selection of the DVS is detailed in Hay *et al. *[1]. In brief, 13 DVS from a final list of 41 species and species complexes worldwide were considered, seven of which are found solely in Africa (Table 1) [1] with a further six distributed across Europe, the Middle East and in limited areas of northern Africa (Table 2).

**Table 1:** Defining the dominant *Anopheles* vector species and species complexes of human malaria in Africa.

Anopheline species or species complex	**White **[260]	**Service **[253,321]	**Kiszewski **[322]	**Mouchet **[223]	**Exc**.	**Inc**.	EO source
*An. arabiensis*	y	y	y	y	1	1	[260]; updated by TAG, 2009
*An. funestus*	y	y	y	y	1	1	[10]; updated by TAG, 2009
*An. gambiae*	y	y	y	y	1	1	[11]; updated by TAG, 2009
*An. melas*	y		y			1	[11]
*An. merus*			y			1	[10]; updated by TAG, 2009, 2010
*An. moucheti*				y		1	[10]; updated by TAG, 2009
An. nili*				y		1	[10]

The * denotes that a "species" is now recognized as a species complex. The exclusive (Exc.) column counts those species identified in all four reviews. The inclusive (Inc.) column counts those species identified by any of the four authors and are the candidate DVS considered for mapping. All of the African species are found in Macdonald's malaria epidemiology zones 6 and 7 (Afrotropical - formerly Ethiopian and Afro-Arabian) 320. The final DVS species listed were defined during two separate Technical Advisory Group (TAG) meetings. EO = Expert Opinion.

**Table 2:** Defining the dominant *Anopheles* vector species of human malaria in Europe and the Middle East.

Anopheline species or species complex	**White **[260]	**Service **[253,321]	**Kiszewski **[322]	**Mouchet **[223]	**Exc**.	**Inc**.	EO source
*An. atroparvus*	4, 5	4, 5	4, 5	4, 5	1	1	[260]; Manguin (pers comm, 2009); updated by TAG, 2009
*An. labranchiae*	5	5	5	5	1	1	[260]; Manguin (pers comm, 2009); updated by TAG, 2009
*An. messeae*			4, 5			1	[260]
*An. sacharovi*	5	5	5	5	1	1	[260]
*An. sergentii*	6	6	6	6	1	1	[260]; updated by TAG, 2009
*An. superpictus*	5	5	5	5	1	1	[260]

The exclusive (Exc.) column counts those species identified in all four reviews. The inclusive (Inc.) column counts those species identified by any of the four authors and are the candidate DVS considered for mapping. The numbers given in each of the review author columns record in which Macdonald's malaria epidemiology zones the species can be found: 4 - North Eurasian; 5 - Mediterranean; 6 - Afro-Arabian 320. The final DVS species listed were defined during two separate Technical Advisory Group (TAG) meetings. EO = Expert Opinion.

### Data assembly, data checks and expert opinion maps

Building on the existing Malaria Atlas Project (MAP [64]) library of parasite rate surveys, a systematic search of the published, peer-reviewed literature using online scientific bibliographic databases was performed and augmented with a range of other information previously described [2]. Literature searches were concluded on 31 October 2009 and all citations meeting our search criteria [2] were reviewed.

Occurrence data extracted from these sources (a detailed protocol is given in Hay *et al. *[1]) were subjected to a series of rigorous checks before being migrated from Excel into a web-based PostgreSQL database where a final series of checks were conducted (see Sinka *et al. *[2]).

Globally, the literature search resulted in 3857 publications or reports containing potential data to be reviewed. Of these publications, 2276 fulfilled the inclusion criteria, providing data for 147 countries. A total of 727 sources detailed surveys conducted across 46 countries in Africa with 45 sources found for 49 countries in Europe and the Middle East.

Using EO map overlays (Additional file 1: Expert opinion distribution maps for the seven DVS of Africa and the six DVS of the Europe and Middle Eastern region (Raster prediction files are available on request)), initially digitised from published, authoritative sources (Table 1, 2) and further refined by a Technical Advisory Group (TAG) of *Anopheles *experts (see acknowledgements), preliminary maps were produced displaying the occurrence data for each species. These maps were examined and points that fell outside the EO range were checked and either corrected or the EO maps adjusted to include all confirmed areas of occurrence.

### Boosted Regression Trees, climatic/environmental variables and model protocol

The BRT method [65,66] was chosen to generate the predictive maps of each DVS distribution. In a review comparing 16 species modelling methodologies, BRT consistently performed well [67] and benefits from being flexible (accommodating both categorical and continuous data), using freely available, reliable and well documented R code [68] and producing maps that are simple to interpret and include a ranked list of environmental or climatic predictors [2]. The method is described in full by Elith *et al*. [66] and its implementation for DVS mapping summarised by Sinka *et al*. [2]. The BRT also produces a number of evaluation statistics including Deviance, Correlation, Discrimination (Area Under the operating characteristic Curve: AUC) and Kappa (κ) which are used here as a guide to the predictive performance of each map.

The BRT model was provided with a suite of open access, environmental and climatic variable 5 × 5 km resolution grids, relevant to the ecology and bionomics of the DVS in the African, European and Middle Eastern regions. Each grid has undergone a series of processing steps to ensure all land and sea pixels exactly correspond, and, using nearest neighbour interpolation, to fill in any small gaps in the data due to, for example, cloud cover (see Sinka *et al. *[2]). Where the remotely sensed imagery was available as multi-temporal data, temporal Fourier analysis (TFA) was applied to ordinate the data, generating seven products for each temporal variable: the overall mean, maximum and minimum of the data cycles; the amplitude (maximum variation of the cycle around the mean) and the phase (the timing of the cycle) of the annual and bi-annual cycles [69]. The environmental/climatic variables applied to the BRT model included a digital elevation model (DEM) [70-72], precipitation and temperature [73,74], land surface temperature (LST), middle infrared radiation (MIR) and the normalized difference vegetation index (NDVI) (Advanced Very High Resolution Radiometer (AVHRR) [75-78]), and 22 individual categories of land cover plus a further three grouped classes that encompassed flooded areas, forested areas and dry areas (Globcover [79]).

The AVHRR grids (LST, MIR and NDVI) were applied to all DVS except the European species *An. messeae *and *An. atroparvus*. These two species have the most northerly distribution of all the DVS, with *An. messeae *ranging up to 65° north. At these latitudes, the AVHRR satellite data can be problematic. Instead MODIS (MODerate Resolution Imaging Spectroradiometer) [70] data were used because it provides better coverage and fewer data gaps for these northern distributions. The MODIS grids include the Enhanced Vegetation Index (EVI) and LST [70].

Following the same protocol described in Sinka *et al. *[2], numerous model iterations were run to assess the 'optimal' mapping outputs, including assessing the buffer size surrounding the EO range from where pseudo-absences would be drawn, the number of pseudo-absences to apply to the model and the effects of including half weighted pseudo-presence data, allocated at random from within the EO boundary, alongside the occurrence data. As each of these categories required the use of different data inputs to the BRT, statistical comparison using the evaluation metrics was not strictly possible. Therefore the 'optimal' settings chosen are inherently subjective and based on visual examination and comparison of the various maps guided by, but not relying on, the evaluation statistics.

### Bionomics

A full protocol describing the methodology used to extract species-specific bionomic data from the available literature (Table 3, 4) is given in the supplemental information accompanying Sinka *et al. *[2]. The bionomics summary of each species is included to accompany the predictive maps as the success of interventions and control methods, such as ITNs or IRS, in reducing malaria transmission is closely related to the behavioural characteristics of the local DVS. This review does not, however, include detailed information relating to insecticide resistance. This was a purposeful omission as it would not be possible to do full justice to this highly dynamic and important aspect of the DVS within the space confines of the current work. Moreover, insecticide resistance is being addressed in detail by other groups, including those at the Liverpool School of Tropical Medicine and the Innovative Vector Control Consortium (IVCC) [80]. Furthermore, there are a number of comprehensive reviews that have been recently produced that detail insecticide resistance amongst Afrotropical species which should be considered alongside this current work (*e.g. *[31,35,81,82]).

**Table 3:** Citation search results for the bionomics survey of the seven Africa DVS created from the MAP database.

Species	References
*An. arabiensis*	[48,100-114,117,119,121-136,142,150,155,159,171,174,176,178,179,181,182,184,186,191,192]
	[310,323-348]

*An. funestus*	[19,84,86,92,100,106,112,114,122-125,128,129,131,134,141-143,145-159,162,177,181,183,192]
	[331,349-363]

*An. gambiae*	[90,91,101,109,119,122,123,127,131,142,145,149,150,153,154,157,159,174-192,344,348,363-365]

*An. melas*	[109,119,193-197,199,200,348]

*An. merus*	[150,201,203,206,207,211,213,214]

*An. moucheti*	[86,124,145,174,217,219,220]

*An. nili*	[86,129,145,148,149,217,225-228,353,363,366,367]

Filter terms were: 'behaviour', 'behavior', 'larva', 'biting', 'resting' and 'habitat'.

**Table 4:** Citation search results for the bionomics survey of the six European and Middle Eastern DVS created from MAP database.

Species	References
*An. atroparvus*	[229,235-237,240,241,263,365]

*An. labranchiae*	[247,249,254-256,258,259]

*An. messeae*	[263,264,270]

*An. sacharovi*	[265,276,277,281,284,286,287,290-292,294,368]

*An. sergentii*	[103,259,286,300,303-309,369,370]

*An. superpictus*	[256,282,286,287,304,312-315,371-373]

Filter terms were: 'behaviour', 'behavior', 'larva', 'biting', 'resting' and 'habitat'. Due to a lack of contemporary data for these species, searches were supplemented with pre-1985 literature.

## Results

### African DVS

A total of 4581 independent sites, of which 4234 were successfully geo-referenced, reported the presence of one or more African DVS, relating to 9300 (8646 geo-referenced) occurrences (i.e. including one or more temporal sample conducted at one independent site) (Table 5). The following results refer only to geo-referenced data, and of these 3951 sites were at a resolution (points and wide areas, <10 km^2 ^and between 10 and 25 km^2 ^respectively) suitable to be applied to the BRT model (from here on, for simplicity, referred to as points).

**Table 5:** Geo-referenced independent site and occurrence (includes multiple sampling at a single site) data for the seven African species by country.

	Site			Occurrence		
Country	All	Data	Polygons	All	Data	Polygons
Angola	57	56	1	59	58	1
Benin	96	94	2	150	126	24
Botswana	10	10	0	11	11	0
Burkina Faso	310	301	9	603	589	14
Burundi	29	21	8	97	87	10
Cameroon	383	375	8	686	678	8
Central African Republic	3	3	0	3	3	0
Chad	14	14	0	14	14	0
Comoros	80	70	10	80	70	10
Congo	2	2	0	2	2	0
Côte d'Ivoire	84	84	0	172	172	0
Democratic Republic of the Congo	30	23	7	59	52	7
Egypt	0	0	0	0	0	0
Equatorial Guinea	113	93	20	132	103	29
Eritrea	45	31	14	48	34	14
Ethiopia	56	45	11	161	145	16
Gabon	28	28	0	128	128	0
Ghana	106	95	11	118	107	11
Guinea	11	7	4	25	21	4
Guinea-Bissau	45	45	0	74	74	0
Kenya	757	686	71	1599	1500	99
Liberia	4	4	0	4	4	0
Madagascar	198	183	15	603	531	72
Malawi	41	40	1	52	51	1
Mali	166	156	10	350	324	26
Mauritius	2	0	2	2	0	2
Mozambique	80	79	1	180	179	1
Namibia	5	4	1	5	4	1
Niger	28	28	0	69	69	0
Nigeria	190	175	15	343	318	25
Réunion	14	11	3	14	11	3
São Tomé and Príncipe	16	13	3	25	20	5
Saudi Arabia	13	13	0	13	13	0
Senegal	209	207	2	608	606	2
Sierra Leone	11	10	1	83	82	1
Somalia	5	5	0	5	5	0
South Africa	93	92	1	127	126	1
Sudan	125	121	4	355	312	43
Swaziland	7	7	0	7	7	0
Tanzania (United Republic of)	383	365	18	900	824	76
The Gambia	192	174	18	280	256	24
Togo	1	1	0	1	1	0
Uganda	135	129	6	322	314	8
Yemen	11	9	2	16	9	7
Zambia	32	29	3	42	39	3
Zimbabwe	14	13	1	19	18	1

**Total**	**4234**	**3951**	**283**	**8646**	**8097**	**549**

'Data' includes points (≤10 km2) and wide areas (10-25 km2) both of which are used in the BRT model and displayed on the predictive maps (Additional file 3). 'Polygons' include small (25-100 km2) and large (>100 km2) polygons which are not included in the models or shown on the maps.

Data were recorded from a total of 46 countries, 44 of which reported points. The largest number of data were reported from Kenya, with a total of 757 sites (all area types), 686 points and 1599 occurrence data (all area types). In contrast, only one data point was reported from Togo (Kantindi) where *An. gambiae *was found [83] and studies from Mauritius only provided DVS location information, at a polygon level, for two sites. African DVS data were reported from Egypt, but only in the form of a polygon location that could not be successfully geo-referenced. *Anopheles gambiae *was reported from the largest number of countries (34) and from the highest number of point locations (1443), however occurrence data (from point locations only) were greater for both *An. funestus *and *An. arabiensis *(2692 and 2301, respectively) than for *An. gambiae *(2291). The least prevalent species was *An. moucheti *reported from only 66 point locations (Table 6) and Cameroon had the highest diversity of DVS with three sites (Nkoteng, Tibati and Mayo Mbocki) showing the presence of five DVS (*An. arabiensis*, *An. funestus*, *An. gambiae*, *An. nili *and *An. moucheti*) [84-86].

**Table 6:** Geo-referenced and non geo-referenced data by species and area type: 'Point' is all mapped data included in the BRT model: point (≤10 km2), wide areas (10-25 km2) and 'Polygon' details data not incorporated in BRT model: small (25-100 km2) and large (>100 km2) polygons, for the seven African DVS (geographically independent sites (Site) and temporal independent occurrences (Occ)).

	Geo-referenced				Non geo-referenced			
	**Point and wide area ('Point')**		**Polygon**		**Point and wide area ('Point')**		**Polygon**	

**Species**	**Site**	**Occ**	**Site**	**Occ**	**Site**	**Occ**	**Site**	**Occ**

*An. arabiensis*	1196	2301	79	171	108	231	3	3
*An. funestus*	919	2692	100	221	83	148	12	28
*An. gambiae*	1443	2291	64	93	117	190	2	14
*An. melas*	149	240	9	25	1	1	0	0
*An. merus*	73	104	10	18	9	10	0	0
*An. moucheti*	66	184	7	7	2	2	1	3
*An. nili*	105	285	14	14	7	8	2	16

**Total**	**3951**	**8097**	**283**	**549**	**327**	**590**	**20**	**64**

Adult resting collections were the most popular sampling method, with 424 studies collecting females resting inside houses compared to 178 studies that collected females biting indoors. Outdoor resting sampling was comparably rare with 56 studies collecting from outdoor shelters, 22 studies searching inside animal sheds and 21 studies where the details of the outdoor location sampled were not recorded. Outdoor landing catches were conducted in 132 studies and 181 studies collected larvae, relating to 675 point locations.

Molecular techniques examining nucleic acids, which have only been applied for identification on a regular basis since the 1990s [87], were well represented, with 338 studies reporting the use of Polymerase Chain Reaction (PCR) methods. Morphological methods were used in 363 studies, often in conjunction with PCR techniques. At the other end of the scale, salinity tolerance tests were only attempted in four studies and cross-mating experiments only in five.

### European and Middle Eastern DVS

Across the European and Middle Eastern region, 49 countries reported the presence of one or more DVS from 2820 point locations (all locations: 2891), of which 2784 were successfully geo-referenced (all geo-referenced locations: 2848) (Table 7). Relatively few polygon data were reported (all: 71/2891, georeferenced only: 64/2848) and longitudinal studies were also rare, with only 18 studies reporting sampling on more than one occasion at the same site. A total of 3020 geo-referenced occurrence data across all area types, with 2946 from point locations, were compiled. Considering only the geo-referenced data, DVS presence was reported from the most sites in Italy (all sites: 423, point only: 409).

**Table 7:** Geo-referenced independent site and occurrence (includes multiple sampling at a single site) data for the six European and Middle Eastern species by country.

	Site			Occurrence		
Country	All	Data	Polygons	All	Data	Polygons
Afghanistan	2	0	2	9	0	9
Albania	42	42	0	42	42	0
Armenia	4	4	0	5	5	0
Austria	70	69	1	70	69	1
Belgium	68	68	0	72	72	0
Bosnia and Herzegovina	64	64	0	64	64	0
Bulgaria	114	114	0	114	114	0
Croatia	69	66	3	69	66	3
Czech Republic	58	58	0	58	58	0
Denmark	43	43	0	43	43	0
Egypt	30	22	8	85	77	8
Estonia	3	3	0	3	3	0
Finland	31	31	0	31	31	0
France	72	72	0	83	83	0
Georgia	8	8	0	8	8	0
Germany	150	150	0	150	150	0
Greece	121	118	3	128	125	3
Hungary	78	78	0	78	78	0
India	2	0	2	2	0	2
Iran	23	15	8	52	44	8
Iraq	4	0	4	4	0	4
Israel	2	2	0	2	2	0
Italy	423	409	14	427	413	14
Jordan	1	1	0	1	1	0
Kazakhstan	1	0	1	1	0	1
Latvia	4	4	0	4	4	0
Lithuania	9	9	0	9	9	0
Macedonia, the former Yugoslav Republic of	7	7	0	7	7	0
Moldova, Republic of	3	3	0	3	3	0
Morocco	6	4	2	23	21	2
Netherlands	217	217	0	217	217	0
Norway	2	2	0	2	2	0
Pakistan	1	1	0	1	1	0
Poland	110	110	0	110	110	0
Portugal	120	120	0	120	120	0
Romania	138	138	0	139	139	0
Russian Federation	127	122	5	130	122	8
Saudi Arabia	8	8	0	8	8	0
Serbia	107	107	0	107	107	0
Slovakia	25	25	0	25	25	0
Slovenia	35	35	0	35	35	0
Spain	44	41	3	45	42	3
Sweden	198	198	0	198	198	0
Switzerland	61	61	0	61	61	0
Tajikistan	2	2	0	2	2	0
Turkey	32	28	4	63	59	4
Ukraine	14	14	0	14	14	0
United Kingdom	91	91	0	92	92	0
Uzbekistan	4	0	4	4	0	4

**Total**	**2848**	**2784**	**64**	**3020**	**2946**	**74**

'Data' includes points (≤10 km2) and wide areas (10-25 km2) both of which are used in the BRT model and displayed on the predictive maps (Additional file 3). 'Polygons' include small (25-100 km2) and large (>100 km2) polygons which are not included in the models or shown on the maps.

*Anopheles atroparvus *was the species reported most often across the region, found at 1051 geo-referenced locations, of which 1044 were available to be used in the analyses. *Anopheles sergentii *was only present at 35 point locations, and within 11 polygon areas, but these related to a total of 113 occurrence data (102 points, 11 polygons) (Table 8).

**Table 8:** Geo-referenced and non geo-referenced occurrence data by species and area type: 'Point' includes all mapped data included in BRT: point (≤10 km2), wide areas (10-25 km2) and 'Polygon' details data not incorporated in BRT model: small (25-100 km2) and large (>100 km2) polygons, for the six European and Middle Eastern DVS (geographically independent sites (Site) and temporal independent occurrences (Occ))

	Geo-referenced				Non geo-referenced			
	**Point and wide area ('Point')**		**Polygon**		**Point and wide area ('Point')**		**Polygon**	

**Species**	**Site**	**Occ**	**Site**	**Occ**	**Site**	**Occ**	**Site**	**Occ**

*An. atroparvus*	1044	1062	7	7	1	1	0	0
*An. labranchiae*	234	241	10	10	1	3	1	1
*An. messeae*	903	905	14	17	2	2	1	1
*An. sacharovi*	183	241	14	14	12	25	0	0
*An. sergentii*	35	102	11	11	7	7	1	1
*An. superpictus*	385	395	8	15	13	24	4	4

**Total**	**2784**	**2946**	**64**	**74**	**36**	**62**	**7**	**7**

In the European and Middle Eastern region, larval collections were the most common sampling method, with 23 studies sampling at 86 sites. Sampling methods were unknown for a large proportion of the data (1553 sites), of which 1488 related to a single data source [56]. Possibly due to the zoophilic nature of the majority of the European and Middle Eastern species (see below), resting adult females were collected from animal sheds at 85 locations compared to only 31 where resting collections were conducted inside human dwellings. Human landing collections were conducted indoors in only two studies, relating to only three sites, with three studies collecting by outdoor human landing at only eight sites.

Identification methods, amongst those studies that reported them, mainly relied on morphological characteristics and were conducted on specimens from 175 locations. Only 10 studies reported using PCR identification techniques but due to a large number of unknown or unreported methods, this ranked as the second most popular method, and was applied to specimens collected from 67 sites.

### Mapping trials

The results for each mapping trial are given in Additional file 2 (Additional file 2: Summary tables showing evaluation statistics for all mapping trials and final BRT environmental and climatic variable selections for the final, optimal predictive maps). Optimal mapping categories were evaluated visually and using the deviance and AUC statistics, with the caveat that these could only be used as a guide rather than a definitive indication of predictive performance.

The EO mapping test indicated that where random pseudo-presences were created within the EO range, and no real occurrence data were included, the model would predict a high probability of presence within the whole EO range and calculate a high deviance value for all species, indicating an overall poor predictive performance. This was the case for the African species and those from the European and Middle Eastern region, and consistent with the results for the nine DVS in the Americas [2]. Where the hybrid method was used that incorporated both real occurrence data plus 500 half-weighted pseudo-presence points randomly assigned within the EO range, the mapping performance was greatly improved. Maps created using only the real presence data produced a low deviance value, but visually, predictive performance was judged to be poor, possibly due to a paucity of data for some species. It was therefore considered that the hybrid maps performed better overall and are presented here.

The optimal buffer width for the African DVS was judged to be 1500 km, producing the lowest deviance value for five out of the seven species. For the European and Middle Eastern species maps, all buffer widths other than 1000 km had high deviance values for all species. The 1000 km buffer therefore was judged to perform better for all six species and applied consistently to all final maps.

For both the African and the European and Middle Eastern species, a ratio of 10:1 pseudo-absences to presence data (not taking into account the 500, half weighted pseudo-presence created in the hybrid maps) was judged to perform better overall, but for both regions, the number of pseudo-absences appeared to have little effect on the predictive maps.

### Predictive maps

**Figure 1:**
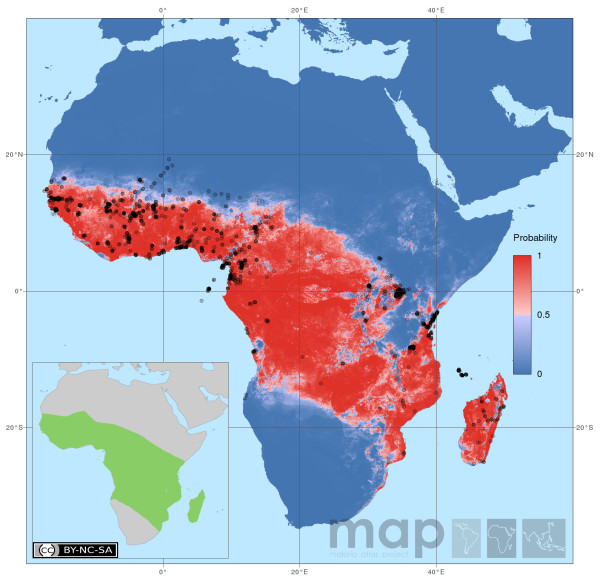
**Map details: The predicted distribution of *An. gambiae *mapped using hybrid data (1443 occurrence data plus 500 pseudo-presences weighted at half that of the occurrence data and randomly selected from within the Expert Opinion (EO) range).** Pseudo-absences (14430) were generated at a ratio of 10:1 absence to presence points, and were randomly selected from within the 1500 km buffer surrounding the EO (EO shown in the inset map). Predictions are not shown beyond the buffer boundary. The black dots show the 1443 occurrence records for *An. gambiae*. Map statistics: Deviance = 0.114, Correlation = 0.9195, Discrimination (AUC) = 0.989, Kappa = 0.9003. Environmental variables: 1. Prec (mean), 2. Prec (max), 3. DEM, 4. Prec (A2) 5. LST (min), (Please see Additional file 2 for abbreviations and definitions). Copyright: Licensed to the Malaria Atlas Project [64] under a Creative Commons Attribution 3.0 License. Citation: Sinka *et al*. (2010) The dominant *Anopheles *vectors of human malaria in Africa, Europe and the Middle East: occurrence data, distribution maps and bionomic précis, *Parasites & Vectors *2010, **3**:117.

The BRT maps for all seven African DVS and for the six European and Middle Eastern species are given in Additional file 3 (Additional file 3: Predictive species distribution maps for the seven DVS of Africa and the six DVS of the Europe and Middle Eastern region). Spatial constraints prevent all species being discussed in detail here, however, *Anopheles gambiae *(Figure 1) is the iconic and possibly the most important vector of malaria [88], and therefore is discussed further below.

There have been a number of attempts to model the distribution of *An. gambiae *but the majority tend to focus on single countries and often just map presence points or abundance without further analysis (*e.g. *[44-49]). Continent-wide predictive maps for *An. gambiae *(plus other members of the *An. gambiae *complex) have also been attempted [1,89], making use of satellite-derived environmental or climatic variables [24,52-55] (Table 9). The methods range from simply overlaying presence and absence points over rainfall maps [24] to the application of more complex, spatial ecological niche models [53,55].

**Table 9:** Summary of continent-wide predictive models available in the literature that map the range of An. gambiae in Africa

Reference	Method	Variables selected
Rogers *et al. *[54]	Maximum likelihood	Not given
Lindsay *et al.*[52]	Data Exploration Tool (DET) within Geographic Information System (GIS), Arc/Info (Non-linear regression)	Annual precipitation between 330-3224 mm Maximum annual temperature 25-42°C Minimum annual temperature 5-22°C Mean Max. temp of the wet season 25-38°C Mean Min. temp of the wet season 11-24°C
Coetzee *et al. *[24]	No model but plot presence/absence against mean annual rainfall	N.A.
Levine *et al. *[53]	Ecologic niche modelling	Frost days mentioned as strongly influential No clear influence of other climatic/environmental variables
Moffett *et al. *[55]	Maximum Entropy (Maxent) niche model	Mean temp of coldest quarter Min. temp of coldest month Precipitation of wettest month Altitude Precipitation of warmest quarter landscape
**Current work**	**Boosted Regression Tree (BRT)**	**Mean precipitation** **Max. precipitation** **Altitude (DEM)** **Precipitation - amplitude of the bi-annual cycle** **Minimum LST**

Precipitation, in one form or another, is identified repeatedly in previous models (where these data are presented, Table 9) as an influential variable in predicting the range of *An. gambiae*. Within the top five contributing covariates from the suite applied to the BRT model, precipitation was identified three times, with mean precipitation as the highest contributor with a relative influence of over 37%. Maximum precipitation was placed second (19.42%) with the amplitude of the bi-annual cycle of precipitation ranked forth (8.85%). In common with the Maxent niche model presented by Moffett *et al. *[55], elevation (altitude) and minimum land surface temperature were also identified by the BRT model within the top five influencing climatic/environmental variables (relative influence of 12.36% and 5.68%, respectively).

*Anopheles gambiae *larvae are commonly found in temporary, shallow, small bodies of water, such as puddles in hoof prints, wheel ruts and small ground pools (see below), sites which are only present after rainfall. Hence the high influence of precipitation on the distribution of this species identified by the BRT model corresponds closely with the known bionomics of *An. gambiae*.

The predictive map of *An. gambiae *(Figure 1) loosely follows the boundary and distribution indicated by the EO map (Figure 1, inset) with one clear exception: the large gap in the range over southern Kenya and a large proportion of northern and central Tanzania. This gap may be driven by the presence of savannah-type vegetation [89] more commonly associated with *An. arabiensis*, or the increasing altitude of this region, and may be causal to the identification of elevation as an influencing factor to the distribution of *An. gambiae. *Similar gaps are also seen in the maps produced by Moffett *et al. *[55] and, to a slightly lesser extent, in the map of Levine *et al. *[53]. In Madagascar, Léong Pock Tsy *et al. *[44] identified altitude as a limiting factor for *An. gambiae *with numbers diminishing as altitude increased until, other than two specimens found at 1300 m, it was considered essentially absent over 1000 m. However, in the Kenyan highlands *An. gambiae *is commonly identified up to 2000 m [89-92] and specimens have been confirmed at sites up to 1800 m in Uganda [93]. Sampling across Africa, as stated by Coetzee [88], reflects the distribution of entomologists and not necessarily the distribution of the mosquitoes, and the area within this predicted gap, along with a great swath through central Africa, is clearly lacking in empirical occurrence data. Acknowledging these caveats, and similar ones in parts of the range of many of the DVS, it is obvious that samples from these poorly known areas would help improve substantially our predictive mapping.

### Bionomics of the African DVS

#### *Anopheles arabiensis*

*Anopheles arabiensis*, when compared to *An. gambiae*, is described as a zoophilic, exophagic and exophilic species [94]. However, it is also known to have a wide range of feeding and resting patterns, depending on geographical location [11,95,96]. This behavioural plasticity allows *An. arabiensis *to adapt quickly to counter indoor IRS control, where suitable genotypes occur [97], showing behavioural 'avoidance' (deterrence from a sprayed surface) depending on the type of insecticide used [95,98].

*Anopheles arabiensis *is considered a species of dry, savannah environments and sparse woodland [11,24,97,99], yet it is known to occur in forested areas, but only where there is a history of recent land disturbance or clearance [24]. Its larval habitats are similar to those of *An. gambiae *(see below): generally small, temporary, sunlit, clear and shallow fresh water pools [100-103] (Table 10), although *An. arabiensis *is able to utilize a greater variety of locations than *An. gambiae*, including slow flowing, partially shaded streams [103-106] and a variety of large and small natural and man-made habitats (Tables 11, 12). It has been found in turbid waters [100,107,108] and, on occasion, in brackish habitats [109] (Harbach, unpub. obs.). It readily makes use of irrigated rice fields (Table 11), where larval densities are related to the height of the rice, peaking when the plants are still relatively short and then dropping off substantially as the rice plants mature [110-113]. Such density fluctuations are also reflected in the adult population, which also peak when rice stalks are small and decline as the plants mature [114-116]. These patterns may be due to a preference for sunlit areas of water with relatively limited emergent vegetation (Table 10), with densities decreasing as shade from the growing plants increases. Moreover, there is evidence that *An. arabiensis *may be attracted by the application of fertilisers or by the amount of dissolved oxygen within the paddy water [111-113,117,118]. However, with fertiliser application occurring at the start of plant cultivation, and dissolved oxygen content related to sunlight exposure (*e.g. *via increasing photosynthesis), the primary oviposition attractant in rice fields is uncertain.

**Table 10:** Larval site characteristics of the African DVS.

Species	Source	Light intensity		Salinity		Turbidity		Movement		Vegetation	
		
		Helio-philic	Helio-phobic	High (brackish)	Low (fresh)	Clear	Polluted	Still or stagnant	Flowing	Higher plants, algae etc	No Veg
*An. arabiensis*	Summary	5	2	1	1	5	5	2	4	11	1

*An. arabiensis*	TAG	●			●	●	○	●		●	●

*An. funestus*	Summary	3	3		1	2		3		6	1

*An. funestus*	TAG	●	○	○	●	●		●	●	●	○

*An. gambiae*	Summary	4	1	1	1	4	4	5	3	5	4

*An. gambiae*	TAG	●			●	●	○	●		●	●

*An. melas*	Summary			5	2					4	

*An. melas*	TAG	●	●	●	○	●	●	●		●	

*An. merus*	Summary			5						2	

*An. merus*	TAG	●	●	●	○	●	●	●		●	

*An. moucheti*	Summary						1	2	2	2	

*An. moucheti*	TAG	●	○		●	●		●	●	●	

*An. nili*	Summary	1							1	1	

*An. nili*	TAG	○	●		●	●		●	●	●	

TAG: Bangs & Mbogo (unpub. obs., 2010), ● = typical, ○ = examples exist. Numbers indicate the number of studies that found larvae under each listed circumstance.

**Table 11:** Large larval sites of the African DVS.

Species	Source	Large natural water collections					Large man-made water collections				
		
		Lagoons	Lakes	Marshes	Slow flowing rivers	Other	Borrow pits	Rice fields	Fish ponds	Irrigation channels	Other
*An. arabiensis*	Summary			1	2	3	2	16	1	2	2

*An. arabiensis*	TAG		●	●	○		●	●	●	●	

*An. funestus*	Summary		1			2		5			1

*An. funestus*	TAG		●	●	●		○	●	●	●	

*An. gambiae*	Summary			1		3		2			2

*An. gambiae*	TAG		●	●	○		●	●	●	●	

*An. melas*	Summary			1		3					

*An. melas*	TAG	●									

*An. merus*	Summary			1		1					

*An. merus*	TAG	●	○								

*An. moucheti*	Summary				2				1		

*An. moucheti*	TAG		●		●			●	●		

*An. nili*	Summary				4						

*An. nili*	TAG				●			●		●	

TAG: Bangs & Mbogo (unpub. obs., 2010), ● = typical, ○ = examples exist. Numbers indicate the number of studies that found larvae under each listed circumstance.

**Table 12:** Small larval sites of the African DVS.

Species	Source	Small natural water collections						Small man-made water collections							Artificial sites
		
		Small streams	Seepage springs	Pools	Wells	Dips in the ground	Other	Overflow water	Irrigation ditches	Borrow pits	Wheel ruts	Hoof prints	Puddles near rice fields	Other	Empty cans, shells etc.
*An. arabiensis*	Summary	4	1	22	8		11		3	4	4	4	4	10	3

*An. arabiensis*	TAG	●	●	●	●	●		●	●	●	●	●	●		○

*An. funestus*	Summary	4					1							2	

*An. funestus*	TAG	●	●	●	●	●		●	●	○	○	○	○		○

*An. gambiae*	Summary	1		10		2	3			2	1	2	2	6	1

*An. gambiae*	TAG	●	●	●	●	●		●	●	●	●	●	●		○

*An. melas*	Summary			3		1	3					1			1

*An. melas*	TAG			●											

*An. merus*	Summary			3			3								

*An. merus*	TAG			●		●									

*An. moucheti*	Summary														

*An. moucheti*	TAG	●		●											

*An. nili*	Summary														

*An. nili*	TAG	●							●						

TAG: Bangs & Mbogo (unpub. obs., 2010), ● = typical, ○ = examples exist. Numbers indicate the number of studies that found larvae under each listed circumstance.

The behavioural variability of *An. arabiensis *is clearly evident (Table 13), with similar numbers of studies reporting either anthropophilic or zoophilic behaviour. Bøgh *et al. *[119] stated: 'There is... great variation in the feeding preference depending on the local variation in host availability and composition of the local genotypes of the vector' [95,96,120]. Tirados *et al. *[121] suggested the existence of an east-west behavioural cline. They proposed that those populations found in western Africa display higher levels of anthropophily, and preferentially feed and rest indoors, whereas those in the east exhibit greater zoophily and rest outdoors. Overall, however, biting patterns tend to be exophagic [121-124], but such behaviour is often reported in comparison with highly endophagic species such as *An. gambiae. *For example, Fontenille *et al. *[125] reported *An. arabiensis *as 'more exophagic than *An. gambiae *and *An. funestus*' with 65.4% of vectors found biting outdoors identified as *An. arabiensis*, yet 59% of those found biting indoors were also identified as *An. arabiensis*.

**Table 13:** Adult feeding and resting behaviour of the African DVS.

Species	Source	Feeding habit		Biting habit		Biting time				Pre-feeding resting habit		Post-feeding resting habit	
		
		Anthro-pophilic	Zoo-philic	Exo-phagic	Endo-phagic	Day	Dusk	Night	Dawn	Exo-philic	Endo-philic	Exo-philic	Endo-philic
*An. arabiensis*	Summary	11	14	8	6		2	9		6	3	12	7

*An. arabiensis*	TAG	●	●	●	○		●	●	●	○	●	●	●

*An. funestus*	Summary	19	6	11	13			11	3	3	13	4	17

*An. funestus*	TAG	●	○	○	●		●	●	●	●	●	○	●

*An. gambiae*	Summary	12	4	10	10			13	3	3	4	5	5

*An. gambiae*	TAG	●	○	○	●		●	●	●	○	●	●	●

*An. melas*	Summary	5	3	3	3		1	3	1		1	3	1

*An. melas*	TAG	●	●	●	○		●	●	●	●	●	●	●

*An. merus*	Summary	3	2	3				1		2		4	1

*An. merus*	TAG	●	●	●	○		●	●	●	●	●	●	●

*An. moucheti*	Summary	5		2	5			1			2	1	3

*An. moucheti*	TAG	●	○	○	●		●	●	●	●	●	●	●

*An. nili*	Summary	6		7	7			2			2	1	2

*An. nili*	TAG	●	●	●	●		●	●	○	●	●	●	●

TAG: Bangs & Mbogo (unpub. obs., 2010), ● = typical, ○ = examples exist. Numbers indicate the number of studies that found adults under each listed circumstance.

Blood feeding times also vary in frequency but biting generally occurs during the night. Peak evening biting times can begin in the early evening (19:00) or early morning (03:00) [121,123,126-131] (Table 13). This species does, however, demonstrate a predisposition to exophilic (or partial exophilic) behaviour regardless of where it has blood fed or the source of its meal [121,125,130,132-135], a behavioural trait considered to be related to polymorphic chromosomal inversions, to a greater or lesser extent, depending on location [97,132,136,137].

#### *Anopheles funestus*

*Anopheles funestus *is a member of the Funestus Subgroup [138] (often mistakenly referred to as *An. funestus *complex), which includes: *An. aruni*, *An. confusus*, *An. funestus*, *An. parensis *and *An. vaneedeni. *The members of this subgroup exhibit important variation in their biology and behaviour, especially in regard to malaria vectorial capacity and are only morphologically distinguishable during certain stages in their development [10,11,18,139]. Only *An. funestus *is regarded as an important vector of malaria in this subgroup [18].

A typical *An. funestus *larval habitat is a large, permanent or semi-permanent body of fresh water with emergent vegetation, such as swamps, large ponds and lake edges. Larvae have been found in shaded and sunlit environments (Table 10) and Gillies & de Meillon [10] concluded that *An. funestus *uses emergent vegetation as refuge against predation while the shading it casts, or the presence of shade from overhanging plants, is of lesser importance. In some areas, *An. funestus *larvae, as with *An. arabiensis*, are associated with rice cultivation (*e.g. *Madagascar, Mali) [140-144] (Table 11). Where they are found, their favoured environmental conditions are very different to those of *An. arabiensis*. *Anopheles funestus *replaces *An. arabiensis *in a successive temporal process during rice plant growth, exhibiting higher densities in older, maturing fields compared to the preceding open conditions preferred by *An. arabiensis *[115,143,144].

*Anopheles funestus *is considered to be highly anthropophilic [10,86,122,145-151] (Table 13) (but see below), which led Charlwood *et al. *[19] to propose that *An. funestus *may have been the first anopheline species to specialise on biting humans, surmising that its preferred larval sites (permanent water bodies in savannah-like environments) are likely to have been areas where humans first settled. Behaviourally, its late-night biting patterns would also allow ready access to human blood without incurring undue density-dependant host avoidance. This late-night biting preference is clearly evident throughout its range, with all studies reviewed reporting a peak biting period occurring after 22:00, and most commonly between midnight and the early hours of the morning [123,124,128,131,145,152-157] (Table 13). Endophilic resting behaviour is also commonly reported [84,86,114,124,125,145,146,149,152,156,158,159], and combined with a relatively high longevity, makes it as good a vector, or better in some areas, as *An. gambiae *[10,11,18,160]. These characteristics are also responsible for promoting the success of vector control using IRS and ITNs. However, this exposure has resulted in selection pressure and rapid development of insecticide resistance to pyrethroids, now well established in some populations and implicated as the primary reason for a major resurgence of epidemic malaria reported in Kwazulu-Natal, South Africa in the late 1990s [18,161].

Compared to other DVS in Africa, *An. funestus *shows fairly consistent behaviour (generally anthropophilic and endophilic) throughout its range; however, it is a highly adaptable species, allowing it to occupy and maintain its wide distribution and utilise and conform to the many habitat types and climatic conditions contained therein. Behavioural differences between chromosomal forms have been identified, for example, Lochouarn *et al. *[162] reported anthropophilic behaviour in western Senegal and zoophilic behaviour in the east of the country, behaviours which correspond to chromosomal polymorphisms that also follow this east-west cline. Costantini *et al. *[60] identified two chromosomal forms in Burkina Faso associated with different resting and biting behaviour. This, coupled with a lack of heterokaryotypes in areas where the two forms co-exist, prompted these authors to suggest that the two forms were incipient species, and hence of the concept of an *An. funestus *complex. More recently, *An. funestus *populations from 12 countries have been divided into three molecular types: M, W, and MW, correlating to geographical locations, whereby M is essentially found in eastern Africa, W from western and central Africa, and MW from southern Africa [61]. Further investigations showed a more complicated situation with specimens from Malawi showing all three types, specimens from Tanzania showed the M- and MW-types, whereas specimens from Kenya showed M- and W-types. In addition, two more types were described, type Y from Malawi, and type Z from four localities of Angola, Malawi, Ghana and Zambia [62]. Finally, adding further to the complexity surrounding this species, recent studies in Malawi have revealed a new species of the subgroup, named *An. funestus*-like [63] that is identical to *An. funestus *but appears to have a different biology and role in malaria transmission, although this needs confirmation.

#### *Anopheles gambiae*

*Anopheles gambiae *is considered to be one of the most efficient vectors of malaria in the world and is one of the most well studied [88]. Like *An. funestus*, the variable ecological conditions present within the large geographical range of *An. gambiae *indicate a highly plastic species with corresponding chromosomal diversity currently separated into five chromosomal forms: Forest, Bamako, Savanna, Mopti and Bissau [163]. There is suggestion of reproductive isolation among the sympatric forms, and hence, of incipient speciation between them [163-165]. Independent of these chromosomal categories, two molecular forms, 'M' and 'S', have also been described [165], and are the forms more commonly referred to in the recent literature. These different forms exhibit ecological adaptations which further indicate possible speciation, for example the Mopti and M forms are associated with semi-permanent, often man-made, larval habitats such as rice fields or flooded areas, whereas the Savanna/Bamako and S forms are seen more commonly in temporary, rain-dependent sites such as ground puddles [166-171]. There appear to be no definitive studies that explicitly describe variability in adult biting or resting behaviour or role in malaria transmission between the two molecular forms.

Despite its wide range and variable ecology, a combination of traits allows *An. gambiae *to maintain its position as one of the most efficient vectors in sub-Saharan Africa. It is a relatively long-lived species (although not as long as *An. funestus *[160]) [172,173], with a short larval development period and is often found in larval habitats associated with human activity (*e.g. *water in hoof prints, wheel ruts or areas of rice cultivation) (Tables 11, 12). It is considered to be highly anthropophilic, with 11 of 15 studies that examined biting behaviour (Table 13) reporting a marked preference for human hosts [131,145,149,150,157,159,174-177]. However, there are a number of studies that indicate *An. gambiae *is less discriminant and more opportunistic in its host selection and that host choice is, as with the majority of African DVS, highly influenced by location, host availability and the genetic make-up of the mosquito population. Moreover, many studies that report host preference using blood meal analysis are often conducted on resting, blood-fed specimens collected inside houses, thus introducing a potential study design or sampling bias favouring the likelihood that the blood meal will be from a human host [178]. Of the studies that report some level of zoophily, Diatta *et al. *[178] specifically examined the host preference of *An. gambiae *and *An. arabiensis *by comparing the number of females of each species captured either in a calf-baited or a human-baited net trap. There was no statistical difference between the host preferences of the two species, both expressing greater zoophily (*e.g. *31% of *An. gambiae *were found in the human-baited trap and 69% in the calf-baited trap). Duchemin *et al. *[122] also reported zoophilic behaviour, yet highlighted this as unusual, suggesting that the high density of cattle in the sampling area may have influenced the propensity for zoophily in the population. Bøgh *et al. *[119] reported no specific preference for either human or animal hosts but that *An. gambiae *would feed readily on cattle.

As with *An. arabiensis*, *An. gambiae *larvae typically inhabit sunlit, shallow, temporary bodies of fresh water such as ground depressions, puddles, pools and hoof prints (although see above) [91,101,175,179-183] (Table 10, 12). Gillies & de Meillon [10] suggested that this aspect of their bionomics allow members of the *An. gambiae *complex to avoid most predators, and the larvae are able to develop very quickly (~six days from egg to adult under optimal conditions and temperatures), possibly in response to the ephemeral nature of their larval habitats. Water in these larval sites can appear clear, turbid or polluted [101,180,184-186] (Table 10). Typically *An. gambiae *larval habitats are described as containing no (or very sparse) vegetation (Mbogo, unpub. obs.) due to their temporary nature. Gillies & de Meillon [10] summarised the great diversity of habitats utilised by *An. gambiae*, and as described before, different molecular or chromosomal forms are associated with either vegetated (*e.g. *rice fields) or temporary and non-vegetated (*e.g. *hoof prints) larval sites [101]. The studies reviewed here report *An. gambiae *from habitats containing floating and submerged algae, emergent grass, rice, or 'short plants' in roadside ditches and from sites devoid of any vegetation [91,101,109,180,181,183] (Table 10).

Females of *An. gambiae *typically feed late at night, a characteristic shared with *An. funestus *that may increase their ability to effectively transmit malaria parasites (see above) [19,123,127,145,153,154,157,175,177,185,187-190] (Table 13). *Anopheles gambiae *is often described as an endophagic and endophilic species, both biting and resting indoors, however, the majority of studies listed herein (nine of 11), that compared indoor and outdoor human-landing catches reported no difference in the numbers of females collected at either location [123,127,145,149,153,157,175,190,191] and an equal number of studies recorded post-feeding exophilic resting [122,131,154,175] as resting indoors [145,149,159,178]. Bockarie *et al. *[175] linked differences in the exo-or endophilic behaviour of *An. gambiae *to their chromosomal forms, suggesting the Forest form (with no inversion) demonstrated stronger exophily in southern Sierra Leone whereas the Savannah form, with a 2La inversion, was mostly endophilic. Odiere *et al. *[192] used clay pots to sample outdoor resting females in western Kenya and found no clear preference for indoor or outdoor resting. They suggested that the designation of *An. gambiae *as a predominantly endophilic species may have been based on poor sampling comparisons. As with host preference, this species appears to exhibit greater phenotypic plasticity and opportunism in blood feeding and resting locations than commonly thought.

#### *Anopheles melas*

There is relatively little contemporary information about the behaviour of *An. melas*, perhaps because it is generally considered to be a vector of lesser importance, specifically where it occurs in sympatry with *An. gambiae *or *An. arabiensis*. *Anopheles melas *has a comparably lower sporozoite rate than either *An. arabiensis *or *An. gambiae *(*e.g. *0.35% compared to 3.5% for *An. gambiae *in The Gambia) [13,95,193], yet in coastal areas where it can occur in very high densities it is still a problematic vector of malaria [13]. With the dearth of available contemporary data, those studies conducted prior to 1985 that closely examined the behaviour of this species have been included here.

*Anopheles melas *is commonly associated with brackish water and can utilise saline environments that other species, for example, *An. gambiae*, cannot tolerate [109,171], yet does not appear to require brackish water for larval stage development [194-196]. It is generally restricted to coastal areas [194-197] but has been found up to 150 km inland along the Gambia River, where salt water can intrude great distances (up to 180 km) upriver [109,171,193]. Unlike other African DVS, the density fluctuations of *An. melas *are closely associated with tidal changes rather than seasons, for example, Gelfand [194] identified a peak in adult numbers 11 days after spring tides. The larvae of this species are associated with salt marsh grass (*Paspalum *spp.) and mangroves, but only trees of the genus *Avicenna*, which include white, grey and black mangrove, and not those from the genus *Rhizophora *('true' or red mangrove spp.) [109,194,195,197]. These positive and negative associations with mangroves are thought to be strongly influenced by the predominant soil type associated with the different tree genera. *Anopheles melas *preferentially oviposits on damp ground at low tide, rather than in open water, where the eggs are able to survive some degree of desiccation [196] until the tides rise again, and appears to prefer the poorly drained, peaty-like soil common to *Avicenna *forests compared to the sandy, gravelly or smooth, fibrous peat soils common to the *Rhizophora *stands [195,198]. Giglioli [198] surmised, that this behaviour guarantees the larvae will have sufficient time to complete their larval development and pupate in the less saline, relatively permanent waters of the new tide before it begins to recede and the water either becomes too salty, or dries out completely.

Adult biting behaviour appears to be opportunistic. *Anopheles melas *has been described as both highly anthropophilic and a zoophilic species [193,194,197,199,200]. In a choice experiment, Muirhead-Thomson [197] varied the numbers of animal and human baits in traps to attempt to describe host preference. He found *An. melas *to be fairly indiscriminate: where there were more animal baits, *An. melas *would feed more often on animals, but still feed on humans. On the contrary, where there was an increase in the number of human hosts, a sharp decrease in the number of females feeding on animals occurred. Sampling bias towards anthropophily may be reported when blood fed females collected resting inside houses are tested for host blood type because *An. melas *generally appears to rest outdoors after feeding [193,194,197], although there has been limited success in locating and collecting from such natural outdoor resting sites. As previously described for *An. gambiae*, those females that bite and rest indoors are more likely to have fed on humans, and those biting or resting outdoors (or in animal sheds) are more likely to have bitten animals. Blood feeding activity appears to be fairly continuous throughout the night [194,197,200]. Gefland [194] observed continual biting from 19:00 to dawn, although Muirhead-Thomson [197] saw two peaks of biting activity: the first, and slightly smaller peak, between midnight and 02:00 and a second, larger peak, between 04:00 and dawn.

#### *Anopheles merus*

*Anopheles merus *has previously been considered as only a minor, or even an unimportant vector, potentially unable to sustain malaria transmission alone [95]. However, is has been identified as playing an 'unexpectedly important role' along the Tanzanian coast [14] and more recently in Mozambique [15]. It is also a species for which there is limited contemporary information. The differences in egg and larval morphology that distinguish *An. melas *from *An. gambiae *do not occur in *An. merus *and identification, before the advent of molecular techniques, was based on physiological characteristics involving larval salinity tolerance tests [201]. Originally, *An. merus *was referred to as a 'salt water *An. gambiae*' variant or subspecies. Indeed, Jepson *et al. *[202] had a number of specimens collected in the 1940s from saline, coastal swamps in Mauritius examined for distinguishing features, and found no obvious morphological distinguishing characters and stated 'All the specimens proved to be typical forms [of *An. gambiae*] and there was no evidence of the presence of *An. gambiae *var. *melas*'. They continued to regard '*An. gambiae*' as a species with 'a considerable tolerance for pollution and salinity and is therefore to be found in domestic wastes and in crab holes and pools near the sea side, in addition to a host of natural breeding places such as marshes, rock pools and casual rainwater pools'. This Mauritian species was finally designated a subspecies of *An. gambiae *by Halcrow [203], who provisionally named it *An. gambiae litoralis *based on larvae found in '...water of high salinity in crab holes, depressions in coralline rocks, small tidal lagoons, pools close to tidal zone and [interior] salt pans, and are not associated with mangroves...' [203,204]. Paterson [205] provided definitive proof of the specific status of *An. merus *and the validity of the name [206].

Halcrow's [203] description highlights a specific difference between *An. merus *and *An. melas*. *Anopheles merus *is rarely found in the mangrove forests on the east coast, however this may be due to the composition of the trees and soil type under of the stands of mangrove in this zone rather than inherent behavioural differences between the two species [10]. *Anopheles merus *is, instead, found in high numbers in shallow brackish pools and marsh or swamp areas along the coast. As a consequence, this species does not exhibit density changes in response to the tidal fluctuations as seen with *An. melas*, nor does it appear to tolerate the same high levels of salinity [201,207]. *Anopheles merus *is also known to occur further inland, using salt pans and saline pools larval habitats [11,208-211], and cross-mating experiments between inland and coastal populations have produced viable offspring indicating they are conspecific [212].

The biting behaviour of *An. merus *is similar to that of *An. melas*: generally opportunistic in host selection, depending on host availability [203,213] and with a tendency to bite [207,214] and rest outdoors [201,206,213,214]. Gillies & de Meillon [10] suggested that *An. merus *shows a preference for animal hosts, referring to a laboratory test where, given a choice, females consistently fed on calf *versus *human bait. Two of the studies reviewed here reported anthropophily [150,214], one indicated zoophily [203] and another concluded that no obvious preference was detected [213]. In the latter study, blood meal analysis was conducted on mosquitoes collected resting indoors (59.2% had fed on humans), and those collected resting outdoors (71.4% had fed on cattle and only 1.6% contained human blood) [213], highlighting the bias in drawing conclusions on host preference if only indoor or outdoor resting specimens are tested. Only one study, conducted on the Kenyan coast, examined the biting times of *An. merus *[214], which reported the number of bites gradually rising from early evening (18:00) peaking between midnight and 01:00 and then declining to 06:00 which corresponds to the accepted biting pattern for this species across its range (Bangs and Mbogo, unpub. obs.).

#### *Anopheles moucheti*

*Anopheles moucheti *is a species with two morphological forms: *An. moucheti moucheti*, and *An. m. nigeriensis *which are distinguishable by morphological features of the adult and larval stages [10]. *Anopheles m. bervoetsi*, previously considered a third morphological form, has recently been raised to full species status: *An. bervoetsi *by Antonio-Nkondjio *et al. *[215]. However, these authors do assert a level of caution in this new status as they point out that *An. bervoetsi *has only ever been reported from its type locality (Tsakalakuku, DRC) and has never been found in sympatry with *An. moucheti*. They do cite unpublished data that detected *P. falciparum *infection in *An. bervoetsi *specimens, and thus raises the possibility that this species could be transmitting malaria in central Africa [215]. The bionomic information detailed here is, in the most part, taken from sources that present data for '*An. moucheti*'. Of these, the majority of studies have been conducted in Cameroon by Antonio-Nkondjio and colleagues or in Nigeria, so based on current knowledge the assumption is that these data refer to *An. moucheti *and not *An. bervoetsi.*

Despite its status as a DVS, *An. moucheti *is a poorly studied species. It is the only DVS with its range entirely restricted to forested areas [216], specifically where the canopy is broken allowing sunlight to penetrate to the ground, such as is found where large rivers flow through the forest [10]. Human activity, such as road building, settlements or cultivation, can therefore be beneficial to this species by breaking up the forest canopy, although larger areas of deforestation may decrease the density of *An. moucheti *and allow replacement by *An. gambiae *[217,218]. *Anopheles moucheti *larvae are found at the edges of large, slow flowing or lentic rivers, often with turbid waters, and are associated with *Pistia *spp (water lettuce/water cabbage) [89,217,219]. Antonio-Nkondjio *et al. *[217] studied the larval habitats along the river networks of southern Cameroon and found the greatest numbers of *An. moucheti *larvae along the margins of rivers within deep, evergreen forest, substantially fewer in the degraded forest and none in the savannah areas. Where they were found, larvae were abundant near to areas of human habitation.

Although the range of *An. moucheti *is relatively restricted within the equatorial forests, it derives its status as a DVS from its highly anthropophilic and endophilic behaviour (Table 13) [86,145,174,219,220]. Gillies & de Meillon [10] suggested such behaviour is unsurprising due to the lack of domestic animals found within forested environments. *Anopheles moucheti *is also described as highly endophagic, however this characteristic appears to be less than clear cut. For example, Antonio-Nkondjio *et al. *[220] found that in urbanised, forested environments (where *An. moucheti *was less abundant and replaced by *An. gambiae*) compared to rural localities (where *An. moucheti *was dominant), only 43% of females were found biting indoors, whereas in the rural areas 66% were found biting indoors. In a study conducted in a village only 2 km from Yaounde, Cameroon, Antonio-Nkondjio *et al. *[145] reported 51% biting indoors and described the sampled populations as 'mainly endophagic'. Overall, *An. moucheti *appears endophilic [86,145] (Table 13). In a countrywide survey of Cameroon, of all females found resting, 1234 were located indoors, whereas only 12 were captured in outdoor shelters [86]. Only two studies examined the biting cycle of *An. moucheti*, with both reporting biting gradually increasing towards the second half of the night to dawn [145,221]; Mattingly [221] reported peak biting activity in the early morning between 03:15 and 06:15.

#### *Anopheles nili* complex

The *An. nili *complex includes *An. carnevalei*, *An. nili*, *An. ovengensis *and *An. somalicus *[12]. As with *An. moucheti*, species of this complex have been generally overlooked in African vector studies despite being described as highly efficient vectors [6,89,222,223]. Amongst members of the complex, *An. nili *is considered the most important vector, although *An. carnevalei *and *An. ovengensis *are implicated as secondary vectors of *P. falciparum *in Cameroon [86,224]. *Anopheles somalicus *is considered zoo- and exophilic [6,10]: it was not found to bite humans in Somalia [10] and no females were found in houses in Cameroon despite an abundance of larvae in the area [6].

Larvae of all members of the *An. nili *complex are found in vegetation at the edges of fast flowing streams and rivers [10,89,195,217]. However, *An. ovengensis *and *An. carnevalei *appear to be restricted to areas of deep forest, whereas *An. nili *is more abundant along rivers in degraded forest and savannah [217]. A comprehensive survey of the river systems across Cameroon found *An. nili *larvae associated with sunlit sites whereas *An. carnevalei *larvae were more commonly found in shaded areas [217].

*Anopheles nili *is considered to be strongly anthropophilic [10,86,145,148,225-227], and will readily bite both indoors and out [145,149,226,228] (Table 13). Carnevale & Zoulani [226] described biting patterns that exploited the behaviour of their human hosts, biting outdoors in the early evening when people are socialising, and then continuing to bite indoors once people move inside, with peak feeding occurring after midnight [145]. The resting habits of *An. nili *are also described as 'variable' [10]. Krafsur [227], in a lowland region of western Ethiopia, rarely found *An. nili *resting indoors despite the high densities found biting indoors, indicative of exophilic behaviour. Conversely, Antonio-Nkondjio *et al. *[86] examined populations across Cameroon and reported *An. nili *overwhelmingly resting indoors (466 females), with only one female captured in an outdoor shelter. In the same study they found no *An. carnevalei *females resting indoors or in outdoor shelters whereas all resting *An. ovengensis *captured were found indoors. Conversely, Awono-Ambene *et al. *[224] stated that *An. ovengensis *was rarely found resting indoors and concluded it had 'exophilic habits'.

### Bionomics of the European and Middle Eastern DVS

#### *Anopheles atroparvus*

*Anopheles atroparvus *is a member of the Maculipennis Subgroup, which also includes *An. *(*Ano.*) *daciae*, *An. *(*Ano.*) *labranchiae*, *An. *(*Ano.*) *maculipennis*, *An. *(*Ano.*) *martinius*, *An. *(*Ano.*) *melanoon*, *An. *(*Ano.*) *messeae*, *An. *(*Ano.*) *persiensis *and *An. *(*Ano.*) *sacharovi *[12]. Of these, *An. labranchiae*, *An. messeae *and *An. sacharovi *are also designated as DVS (see below).

*Anopheles atroparvus *is described as a species with a preference for brackish larval habitats [229-232]. Hackett & Missiroli [231] summarised: 'In general it may be said that over its extensive range [*An.*] *atroparvus *is found in water of moderate salinity not exceeding 10 parts per 1000. It prefers relatively cool water, and its range does not overlap that of [*An.*] *labranchiae*, a warm water breeder'. However, the larval sites listed in the literature still include a number of predominantly fresh water habitats, for example canals, ditches, river margins, pools in river beds and rice fields [230], and Cambournac [233] defines *An. atroparvus *as a 'fresh water breeder'. Hackett [234] also stated that, in southern Europe, *An. atroparvus *'inclines to breed in fresh water'. Of the few studies reporting primary data (Tables 14-17), larvae were identified in marshes and ditches/ground flood pools [235], pools in river beds, river margins and streams, rock pools, cement tanks, rice fields, wells and ground pools [229] and in small collections of water in used tyres [236] (Tables 15, 16).

**Table 14:** Larval site characteristics of the European and Middle Eastern DVS.

Species	Light intensity		Salinity		Turbidity		Movement		Vegetation	
	
	Helio-philic	Helio-phobic	High (brackish)	Low (fresh)	Clear	Polluted	Still or stagnant	Flowing	Higher plants, algae etc	No Veg
*An. atroparvus*	1								2	
*An. labranchiae*				1						
*An. messeae*					1		1	1	1	1
*An. sacharovi*	1		3	3			2	2	1	
*An. sergentii*	1	3	2	6	3	1	4	4	6	
*An. superpictus*	4		1	1	4		2	3	3	1

No TAG summary was available for these species. Numbers indicate the number of studies that found larvae under each listed circumstance.

**Table 15:** Large larval sites of the European and Middle Eastern DVS.

Species	Large natural water collections					Large man-made water collections				
	
	Lagoons	Lakes	Marshes	Slow flowing rivers	Other	Borrow pits	Rice fields	Fish ponds	Irrigation channels	Other
*An. atroparvus*			1				1			
*An. labranchiae*		1		2		1	3		2	2
*An. messeae*		2	1	1	1					
*An. sacharovi*		1	3	1	3		2	1	1	1
*An. sergentii*					1		1		3	1
*An. superpictus*		1			4		1		3	

No TAG summary was available for these species. Numbers indicate the number of studies that found larvae under each listed circumstance.

**Table 16:** Small larval sites of the European and Middle Eastern DVS.

Species	Small natural water collections						Small man-made water collections							Artificial sites
	
	Small streams	Seepage springs	Pools	Wells	Dips in the ground	Other	Overflow water	Irrigation ditches	Borrow pits	Wheel ruts	Hoof prints	Puddles near rice fields	Other	Empty cans, shells etc.
*An. atroparvus*	1		1	1		1							2	2
*An. labranchiae*	3		1			1								
*An. messeae*													1	
*An. sacharovi*	1	1	2			1								
*An. sergentii*	2	5	4	2		2			1				2	
*An. superpictus*	3	3	2	1		3		2	1		1		1	

No TAG summary was available for these species. Numbers indicate the number of studies that found larvae under each listed circumstance.

**Table 17:** Adult feeding and resting behaviour of the European and Middle Eastern DVS.

Species	Feeding habit		Biting habit		Biting time				Pre-feeding resting habit		Post-feeding resting habit	
	
	Anthro-pophilic	Zoo-philic	Exo-phagic	Endo-phagic	Day	Dusk	Night	Dawn	Exo-philic	Endo-philic	Exo-philic	Endo-philic
*An. atroparvus*	1	5							5		5	
*An. labranchiae*	2	3	1	1		1	1		6	2	6	2
*An. messeae*	1	2							1	1	1	1
*An. sacharovi*	2	3								4		6
*An. sergentii*	1	6									1	3
*An. superpictus*									1	3	1	3

No TAG summary was available for these species. Numbers indicate the number of studies that found adults under each listed circumstance.

Becker *et al. *[230] described sites to be 'usually sun exposed' and to contain 'a considerable amount of filamentous green algae and other floating submerged vegetation'. Pires *et al. *[229], in a study that sampled comprehensively across Portugal, reported *An. atroparvus *larvae to be found more frequently in sun-exposed habitats, although 'some shade was provided by grasses and aquatic vegetation'. They also reported filamentous algae present in 48 of 93 sites positive for *An. atroparvus *(Table 14).

*Anopheles atroparvus *is generally considered zoophilic [229,230,237], and described as 'very zoophilic' by Cambournac [233], who also stated that its hosts, in order of preference, are rabbit, horse, cow, pig and sheep, and suggested that a long association between rabbit and *An. atroparvus *(since approx. 1000 BC) may be responsible for this hierarchy of preference. Indeed, *An. atroparvus *has been implicated as an effective vector of the myxomatosis virus to domestic rabbits in the UK [238,239]. Elsewhere, however, *An. atroparvus *is described as anthropophilic [89], which perhaps indicates the opportunistic nature of this species. Four studies identify *An. atroparvus *as zoophilic [229,237,240,241] and one study, that did not distinguish a preference, reported the collection of *An. atroparvus *during night catches on horse bait, from indoor resting sites and during day- or night-time catches on humans [235] (Table 17). There is no clear evidence or information among any of the published studies, nor within the general literature, that identifies this species as preferentially biting indoors or outdoors. The opportunistic nature of its feeding habits and zoophilic proclivity in host choice, however, would suggest it is probably exophagic but that biting location could also depend upon the setting and accessibility of the host.

*Anopheles atroparvus *rests and hibernates in animal sheds and stables [229,230,235,237,238,240,241]. It hibernates as an adult female and is known to periodically feed, specifically if she has taken refuge in a relatively warm locality, but these meals do not result in egg production (i.e. gonotrophic disassociation) [230-232].

A number of investigators have discussed the inability of *An. atroparvus *to transmit tropical strains of *P. falciparum*, with most referring to studies conducted by Shute [242]. Unfortunately this reference could not be found, but in a study testing the susceptibility of Russian anopheline species to imported *P. falciparum *[40], no infection was detected in *An. atroparvus *females. Curtis & White [243] concluded (also referring to Shute [242]) that *An. atroparvus *is refractory to both Asian and African *P. falciparum *but competent in supporting a European strain, a conclusion reiterated by de Zulueta *et al*. [39] with Cambournac [233] stating that refractoriness of *An. atroparvus *to African and eastern strains of *P. falciparum *is an 'uncontroversial fact'. However, Capinha *et al*. [244] claimed the existence of local *An. atroparvus *in Portugal that could be infected with 'exotic strains of plasmodia', with reference to a comprehensive study by Souza [245]. However, on closer examination of these findings, even though Sousa did indeed infect *An. atroparvus *with *P. falciparum*, this was only after numerous attempts that resulted in formation of oocysts in only five out of 736 females. It would seem, therefore, that although *An. atroparvus *can be infected by tropical *P. falciparum *strains, it is very unlikely to happen under natural conditions and there is currently no conclusive evidence that such infection would result in salivary gland invasion by sporozoites.

#### *Anopheles labranchiae*

Despite similarity in larval site characteristics, *An. labranchiae *and *An. atroparvus *do not, or only have limited, overlap in their distributions [231]. This lack of sympatry may be simply a factor of temperature, with *An. labranchiae *making use of warmer waters than typical of *An. atroparvus *[230,231]. However, when Capinha *et al. *[244] modelled the habitat suitability of *An. atroparvus *across Portugal, they concluded that the most suitable locations include drier areas with higher temperatures (i.e. conditions where *An. labranchiae *typically dominate), whereas wetter areas with milder temperatures, where *An. atroparvus *are mostly found, were unsuitable. They concluded that *An. atroparvus *is not found in many other 'suitable' Mediterranean areas due to competitive exclusion. Conversely, de Zulueta [246] suggested that the absence of *An. atroparvus *in Sardinia allowed the wide distribution of *An. labranchiae *on the island, where, despite a five-year eradication campaign instigated in 1946, *An. labranchiae *still occurs [247,248].

Both species utilise brackish water marshes and lagoons along the coast [231], although in contrast to *An. atroparvus*, *An. labranchiae *will preferentially oviposit in fresh water [89,247,249-251]. Marchi & Munstermann [247], in a survey conducted across Sardinia, only identified *An. labranchiae *in fresh water sites, including rock holes, pits, ditches, drains or canals, streams/rivers, flooded ground pools and ponds, lakes or reservoirs. Despite an ability to tolerate some salinity, *An. labranchiae *larvae are not generally found at sites with significant levels of organic or mineral pollutants ([252], Mouchet, pers. com.). Larval sites are typically described as sunlit [89,230,249,253], although in Sardinia Aitken [251] found larvae in 'almost every type of habitat except the very densely shaded', and Macdonald [250] also associated this species with habitats that have some level of shade. In general, *An. labranchiae *larvae are found in stagnant or slow moving waters [230,249] and can make use of, and become very abundant in, rice fields [89,253-256]. Indeed, Bettini *et al. *[254] described a survey in central Italy that identified high numbers of larvae in newly established (two years old) rice fields with correspondingly high numbers of adults found resting in animal shelters near these fields.

Female *An. labranchiae *can aggressively attack human hosts [230,255], and are described as 'persistent' in their attempt to enter bedrooms during the night [230]. Nonetheless, this species is also described as zoophilic in some of the general literature, but overall, *An. labranchiae *appears opportunistic in its host choice, readily biting either humans or animals (Table 17) [89,249,250,253,255-257]. Romi *et al. *[256] found high percentages (86% and 90.7%) of females engorged with human blood resting inside houses whereas they also found that almost all specimens collected resting in animal shelters had fed on animals.

*Anopheles labranchiae *rests inside houses, animal shelters, and, to some degree, in natural shelters, depending on the location of its blood source [249,253-259]. D'Alessandro *et al. *[249] described *An. labranchiae *as both endo- and exophilic, using whatever shelters are available. Females hibernate in stables/animal shelters and in natural sites such as crevices and tree cavities. Both incomplete (with occasional blood feeding but without ovipositioning) and complete (with fat bodies, without feeding and non-gonoactive) hibernation have been noted for this species [89,230,231,249].

As with *An. atroparvus*, *An. labranchiae *has been found to be refractory to exotic strains of *P. falciparum*, with de Zulueta *et al. *[39] failing to infect *An. labranchiae*, albeit a small sample, with a Kenyan strain of *P. falciparum*. However, Toty *et al*. [58] reported historical evidence of naturally infected *An. labranchiae*, plus the results of a contemporary study conducted by the Centre de Production et d'Infection d'Anophèles (CEPIA) in Paris where 14% (13/99 specimens) of Corsican *An. labranchiae *were experimentally infected with the African NF54 laboratory-cultured strain of *P. falciparum*. This study also detected sporozoites in the salivary glands of three specimens, indicating that *An. labranchiae *is not only susceptible but also potentially able to transmit at least some strains of African *P. falciparum *[58]. However, this conclusion must only be considered alongside the knowledge that the NF54 *P. falciparum *strain is a highly attenuated, long-standing laboratory culture which may no longer reflect its origins (Bangs, unpub. obs.).

#### *Anopheles messeae*

*Anopheles messeae *is the third member of the Maculipennis Subgroup [12] to be designated as a DVS. It is the most widespread species of the subgroup [230], with a distribution extending from Ireland across Europe and Asia and into China and Russia [260]. A great deal of work on this species has been conducted in Russia and China. This review is therefore presented with the caveat that there may be details and data reported in the Chinese or Russian literature that are not included here due to access difficulties.

Di Luca *et al. *[261] identified a number of genetic polymorphisms within *An. messeae *and defined five separate haplotypes associated with different geographical areas across its distribution. However, they could not confirm whether these polymorphisms were indicative of altered behaviour at these different locations, although the large range of this species combined with such genetic variability would suggest that some area-specific biological or behavioural adaptations are likely to have occurred.

The larvae of *An. messeae *are typically found in shaded, clear, very slow flowing or stagnant, fresh water sites [230,262-264] such as lake margins and marshes [263-265]. Despite only sampling resting females, Adamovic, in Serbia and Montenegro [266-268] and Adamovic & Paulus [269], in surveys of Slovenia and Croatia, continually associated the presence of adult *An. messeae *with stagnant, fresh water oxbow swamps and marshes within alluvial plains or valleys of large river systems and at sites near large lakes. Localities along rivers with saline or alkaline soil did not provide the same association [266,268], however they did report the presence of *An. messeae *at sites near a marshy plain with brackish water [267]. Takken *et al. *[263] also inferred the presence of *An. messeae *in more brackish habitats, presenting a photograph in their paper of a drainage ditch labelled as containing brackish water and vegetation which 'supports *Anopheles messeae*'. However, they also indicated that engineering works in the Netherlands allowed the transition of brackish sites to fresh water, so whether or not they did find this species in brackish water is still unclear. Nonetheless, Takken *et al. *[263] did identify locations where *An. messeae *larvae were collected, including sites containing reeds, and those containing floating aquatic weeds and algae, relatively open ditches inside forests and clear water in small lakes within dunes.

Only one study could be found that sampled *An. messeae *females inside human habitations, animal shelters and in natural outdoor shelters [270]. All other studies only searched in animals shelters [240,263,266-269,271-273]. Where comparisons were made, no *An. messeae *were found resting outdoors in urban areas (*e.g. *in vegetation surrounding buildings) but were found indoors such as in entryways, staircases and basements, although not in large numbers. In rural areas, *An. messeae *dominated the collections made from cattle sheds, with few specimens collected from natural outdoor sites (hollows, ground cavities, amongst vegetation surrounding marshes, ponds and streams) [270].

Takken *et al. *[263] argued that in the Netherlands *An. messeae *has never been considered as a malaria vector despite it being as susceptible to *P. vivax *infection as *An. atroparvus *[40]. They stated that its high degree of zoophily and outdoor feeding behaviour makes the likelihood of it being involved in local malaria transmission very remote. They supported their argument reporting that all resting *An. messeae *females collected in their study had fed on animals; however, all their samples were collected from animal shelters. Bates [265] also mentioned that in Albania, *An. messeae *(along with other members of the Maculipennis Group) is not considered a malaria vector using the same reasoning: '[*An. maculipennis*, *An. messeae *and *An. melanoon *(as *An. subalpinus*)] are generally supposed not to be malaria vectors because of their non-anthropophilous [*sic*] food habits'. Fyodorova *et al. *[270] found that 40% of the *An. messeae *females collected in urban areas contained human blood, with the remaining 60% having fed on cats (40%) and chickens (20%). However, in rural areas, no human blood meal was detected. Becker *et al. *[230] summed up the biting preferences of *An. messeae *somewhat ambiguously, stating that 'Blood-meals are taken from humans only when the density of *An. messeae *is very high and there is a shortage of livestock, but they also may attack humans in houses'. No studies were found that examined the feeding cycle of *An. messeae*.

*Anopheles messeae*, like *An. atroparvus *and *An. labranchiae*, hibernates as an adult female. However, unlike these other two species, *An. messeae *chooses hibernation sites in abandoned buildings, in the absence of animals [230,271]. They enter full diapause, and do not feed during the winter, but instead, gain energy from fat reserves [271].

There is some evidence to suggest that, along with *An. atroparvus*, *An. messeae *may also be refractory (or essentially refractory) to tropical *P. falciparum *strains. In their study, testing the susceptibility of Russian anophelines to imported *P. falciparum*, Daškova & Rasnicyn [40] were unable to infect *An. messeae. *Indeed, the vector status of *An. messeae *has come into question, specifically since the discovery of a new species in 2004, formally named *An. daciae*, in Romania [273], which has since been recorded from south-western England [274]. *Anopheles daciae *can only be distinguished from *An. messeae *using egg morphology or by sequencing the internal transcribed spacer 2 (ITS2) of ribosomal DNA and the cytochrome oxidase 1 (COI) of mitochondrial DNA. It has been suggested that the presence of *An. daciae*, potentially sympatric across the full range of *An. messeae*, may be responsible for the high polymorphism previously reported for *An. messeae *[261,274]. Combined with an ongoing debate about the capacity of *An. messeae *to transmit malaria (*e.g. *it is not considered a vector in northwestern Europe [275]), it is feasible that *An. daciae*, and not *An. messeae*, could be involved in malaria transmission, which will only be confirmed with further investigation into the epidemiological importance of each respective species (Harbach, unpub. obs.).

#### *Anopheles sacharovi*

*Anopheles sacharovi *is the final member of the Maculipennis Subgroup defined as a DVS and has been the target of a number of focussed, anti-vector campaigns across its range including Israel, Greece and Turkey [262,276-279], yet this species still persists in all areas. *Anopheles sacharovi *is highly plastic in both adult behaviour and its choice of larval habitats. Zahar [262] states simply: '[*An. sacharovi*] breeds in all small water collections containing aquatic vegetation'. It makes use of fresh water habitats but is also described as more tolerant of salinity (up to 20%) than any other member of the Maculipennis Subgroup [230,262]. It can survive in waters up to 38-40°C ([280] references within), and although it is generally considered to breed in stagnant waters, it can also cope with some, albeit weak, current [281,282]. Throughout the literature there is general agreement that this species prefers sunlit sites with plenty of emergent and/or floating vegetation [89,230,262,283-285]. A typical habitat would be an area of swamp or marsh [265,279,282], but larvae are also found at margins of rivers, streams and springs [281,282], seepages [281], wadis [286], pools and ditches [265,287]. It is associated with rice cultivation and other irrigated areas, specifically where irrigation channels are poorly constructed causing leakage, creating boggy areas or standing water [89,230,277,279,282,284,288,289]. Despite its apparent adaptability, *An. sacharovi *cannot tolerate organic pollutants [262,285]. Indeed, Saliternik [285] lists the organic pollution of streambed habitats, previously densely populated with *An. sacharovi *larvae, of greater impact than the wide-scale IRS application of DDT as causal to the near elimination of this species in Israel in the 1960s.

*Anopheles sacharovi *females feed opportunistically, despite being generally considered as anthropophilic [89,230]. Only one study reviewed specifically tested host preference. Demirhan & Kasap [290], using baited feeding rooms, concluded that in the presence of other, equally available hosts (human, cow, sheep, chicken, horse and donkey), *An. sacharovi *preferentially fed on donkeys, and had a negative preference for humans. They also analysed the blood meals of engorged females from human habitations, animal shelters and abandoned or ruined buildings and reported the 'feeding preference' of females captured in the human dwellings to be cow, human, sheep, horse and chicken. Other studies reported similar results. Yaghoobi-Ershadi *et al. *[291] found high numbers of females collected from cow sheds or chicken coops had fed on animals (85.6 - 92.5%), whereas of those collected from bedrooms, only 38.5% had fed on humans, 38.5% on other animals and 23% on both. Boreham & Garrett-Jones [292] reported predominantly human blood in specimens collected from houses, predominantly animal blood (sheep or goat) from animal shelters, and those collected from outdoor pit shelters generally contained blood of mixed animal origin (sheep, goat, horse, dog or cow). Hadjinicolaou & Betzios [276] reported a high percentage of females containing human blood from human habitations, whereas females taken in pit shelters and animal sheds had mostly fed on domesticated animals. They concluded that *An. sacharovi *still exhibited significant levels of anthropophily despite a high ratio of animals to people (between 9:1 and 7.2:1) in the study area. Boreham & Garrett-Jones [292] suggested that *An. sacharovi *had increased tendencies towards zoophilic behaviour due to previous DDT spraying campaigns, but was reverting back to anthropophily.

*Anopheles sacharovi*, contrary to the accepted night-time biting habits of most anophelines, can 'in deeply shaded situations... attack viciously throughout the day' [289]. However, Djadid *et al. *[293], indicated that *An. sacharovi *(plus other members of the Maculipennis Subgroup) generally start biting in the early evening, peaking between 20:00 or 21:00 (refers to Djadid MSc thesis), with Alten *et al. *[294] noting higher densities of *An. sacharovi *between 20:00 and 22:00, although they did not specifically examine biting behaviour. Hadjinicolaou & Betzios [276] observed *An. sacharovi *to bite indoors and outdoors. Biting location is likely to be driven by host behaviour, for example, in the hotter parts of Turkey where both people and animals spend the night outdoors, biting would tend towards exophagy [289,294].

*Anopheles sacharovi *is principally described as endophilic [89,230,284]. Its choice of resting location (and, arguably, for all species) is most likely driven by the need to find the most suitable microclimate for increased survival [289]. Demirhan & Kasap [290] observed *An. sacharovi *feeding on cows outside, and then entering houses or abandoned shelters to rest. Yaghoobi-Ershadi *et al. *[291] found *An. sacharovi *in cow sheds, chicken coops and bedrooms, but were unable to find any females resting outdoors. Boreham & Garrett-Jones [292] searched two artificial pit shelters and found 10 and 42 specimens compared to 377 and 333 from two cattle sheds and 260 in a village house. Abdel-Malek [282] failed to find *An. sacharovi *resting outdoors, but again, repeatedly found them resting in animal stables and human habitations. However, insecticide residual spraying in many areas has apparently affected endophilic behaviour [283,295,296], summed up by Gokberk [283]: 'Following the last ten years of DDT spraying, *An. sacharovi *recently began to show a tendency to be less domestic in habits'. Yet, there is evidence that once these IRS programmes ceased, *An. sacharovi *began to revert to more typical endophilic behavioural patterns [276].

As with other European or Middle East DVS that occur in warmer climates, hibernation is incomplete, with intermittent feeding during winter, but without oviposition [89,297], often making use of the same localities chosen for resting in the summer months [288].

#### *Anopheles sergentii*

There is some confusion as to the taxonomic status of *An. sergentii*. It has previously been considered to have two geographical forms: *An. sergentii sergentii *and *An. sergentii macmahoni*, but in accordance with the published literature, the Walter Reed Biosystematics Unit (WRBU) online catalogue of the Culicidae [298] and the Mosquito Taxonomic Inventory [299], *An. macmahoni *is currently considered a subspecies of *An. sergentii*. This subspecies has never been found biting humans and is of no known medical importance [10,300].

*Anopheles sergentii *is known as the 'oasis vector' or the 'desert malaria vector' due to its distribution within oases across the Saharan belt in northern Africa into the Middle East, and its ability to cope with the extreme climate across this region [301,302]. It may be able to survive in such harsh conditions due to its adaptability. It makes use of a range of larval habitats, including streams, seepages, canals, irrigation channels, springs, rice fields [103,230,262,286,300,303-308], and most other non-polluted, shallow sites that contain fresh water with a slow current, slight shade and emergent vegetation or algae (Table 14) [89,103,230,262,302,306,308]. However, larvae have also been found in moderately brackish habitats, areas of stagnant water, light to moderately polluted locations or in sites in full sunlight [303-305,307]. In general, the presence of vegetation or algae seems to be the only characteristic common to all larval habitats of this species [103,230,302,303,305-308].

Farid [302] described *An. sergentii *as '...an indiscriminate biter of both humans and animals, both indoors and out.', however, no study could be found that specifically tested host preference. Six studies have reported blood meal analyses of resting mosquitoes, taken from both human and animal shelters (Table 17). Of these, five described *An. sergentii *as principally or even highly zoophilic [103,259,306,308,309], with Kenawy *et al*. [308] stating the key factor that limits oasis malaria transmission in Egypt is the zoophilic feeding behaviour of *An. sergentii*. Faraj *et al. *[259], in Morocco, also described 'a marked preference for zoophily' in this species.

Kenawy *et al*. [309] related human biting to local animal stabling practices. They found that in villages where animals were housed in rooms within human habitations, a lower proportion of the *An. sergentii *females collected resting in the houses contained human blood. In an earlier study, however, the proportion of females with human blood was higher in those taken from houses containing animal rooms (86%), although the absolute numbers (calculated here from percentages reported in the paper) of 18/21 versus 26/54 (48%) from houses with no animal rooms also indicate that *An. sergentii *were diverted away from human hosts and towards the animals when in close proximity of one another. In this latter study, the animal rooms within the houses had the highest number of resting mosquitoes (209), of which 13% (equivalent to 27 mosquitoes) had fed on humans compared to those in isolated animal sheds (126) with only 7% (equivalent to nine mosquitoes) containing human blood.

No studies were found that specifically tested biting location, yet Abdoon & Alshahrani [310] reported high numbers biting outdoors and concluded *An. sergentii *was both exophagic and exophilic after finding few females resting inside houses. However, with no comparable indoor biting data and no sampling of resting mosquitoes from animal shelters, this conclusion may not indicate a true blood feeding preference. Indeed, Saliternik [285] described *An. sergentii *as feeding and resting both indoors and outdoors, but referred to 'exophilic habits'. Barkai & Saliternik [304] suggested that an 'exophilic strain' of *An. sergentii *had developed because of indoor spraying with DDT in Israel, finding fewer adults at indoor resting places where they had been common in the past, despite the local abundance of larvae.

*Anopheles sergentii *can overwinter as both adult females or larvae [230,302], although no details regarding hibernation, blood feeding and oviposition could be found.

#### *Anopheles superpictus*

Preliminary data on *An. superpictus *populations sampled across Iran recently identified three genotypes (designated X, Y and Z) and raised the possibility of *An. superpictus *as a species complex [311]. These data have yet to be confirmed, but the wide distribution of this species across a number of diverse climatic regions (Mediterranean across to central and southwestern Asia) and the existence of eight junior synonyms, suggests the realistic possibility of *An. superpictus *being a complex of species and therefore warrants further investigation (Harbach, unpub. obs.).

In the published literature, *An. superpictus *larvae are continually associated with gravel or pebble river and stream beds in shallow, slow-flowing clear water in full sunlight [230,250,265,282,285,289,304,312-314]. Typical, natural sites are small pools within or next to drying river beds, conditions which are closely related to seasonal fluctuations in precipitation [230,265,289,314,315]. At such sites, larval abundance increases only in late summer when pools are created as the river levels decline and, once water levels rise with the increasing rain during the onset of winter, these locations again become unsuitable as aquatic habitats [230,289,315].

Such natural limiting conditions could restrict both the distribution, abundance and period of adult activity of this species, however *An. superpictus *has easily adapted to human-influenced habitats, making use of irrigation channels and storage tanks and pools formed from their leakage, rice fields, ditches, borrow pits and hoof prints, amongst others [230,282,313,315,316]. *Anopheles superpictus *larvae have also been found in brackish water habitats [314] and in stagnant water [304,313]. Jetten & Takken [275] state that it can occur in polluted sites, although here, no primary data were found to confirm this statement, which is also contradicted by other observations. For example, Berberian [314], stated that '*A. superpictus *is never found in polluted or filthy water...' and the decline of *An. superpictus *(and *An. sergentii*) in Israel has been closely associated with sewage pollution of many of the natural streams it previously inhabited [285]. *Anopheles superpictus *survives at relatively high altitudes, up to 2800 m [264], replacing *An. sacharovi *[316] that may dominate at lower altitudes.

No publication could be found that reports any definitive host preference for *An. superpictus*, but it is generally given to be a zoophilic species that also readily feeds on humans [230]. Tshinaev [315] reported from Latyshaev [317] (reference unavailable) that, in Uzbekistan 'individuals that who sleep out during the summer on the flat roofs of houses and on towers are not attacked by this mosquito'. Conversely, Ramsdale & Haas [289] stated that *An. superpictus *in Turkey has a 'marked preference for animals but feeds on man in their absence...', but still described *An. superpictus *as an 'unusually dangerous mosquito' for those people who spend nights out in the open, away from villages and towns.

Again, no primary data were found describing biting location. However, *An. superpictus *appears to be opportunistic in its feeding habits and will enter houses to feed [250], but is generally regarded as exophagic [318,319].

## Discussion

The BRT model has been applied to contemporary data on the occurrence of 13 DVS in Africa, Europe and the Middle East using the most comprehensive database of DVS occurrence currently available. These maps and the underlying database will be made available in the public domain. We stress that the predictive maps produced will not be perfect representations of the true geographical distributions of these species but nevertheless, they represent a substantive step in improving our knowledge of the range of those DVS studied. One particular issue for any environmental-niche based mapping technique is predictions in areas that, although environmentally suitable, may not contain the vector for other biogeographical reasons. The model predicts, for example, the presence of *An. arabiensis *throughout Madagascar. *Anopheles arabiensis *is a species commonly associated with dry, savannah-type habitats and is considered absent, or at least, is rarely encountered in the humid climate of the eastern coast of Madagascar (Manguin, unpub. obs.). Conversely, *An. nili *has never been recorded in Madagascar (Manguin, unpub. obs.) but, as identified in the predicted distribution, there are areas where conditions are suitable for *An. nili *to become established if it were to be introduced.

Biases in collection location, variation in sampling methodologies, limited data for some species or an absence of data over large areas of suspected occurrence all contribute to uncertainty in the final predictions. Yet despite these limitations, the maps represent the first attempt to model DVS distributions across Africa, Europe and the Middle East using a combination of extensive occurrence data combined with contemporary EO distributions. All this information should be triangulated when evaluating the utility of the maps which are best considered as the beginning of an on-going process of understanding, describing and better predicting the range of these DVS. This process may be further complicated by the ever evolving revision of taxonomic status of a number of the African DVS that may lead to further stratification of the occurrence data and revisions of the predictions. This is particularly important where newly identified forms are associated with varying bionomics relevant to their control; the molecular and chromosomal forms of *An. gambiae *are but one example.

### Bionomics

The behavioural plasticity, large geographic ranges, and changing taxonomic categorisation, in particular with the African DVS, present challenges when summarising the bionomics of individual species. Moreover, conclusions drawn about behavioural characteristics based on biased sampling may mask the true variability in a population and behavioural adaptation to human influences, such as insecticide use or environmental disturbance, can also influence local variation in species bionomics. The bionomics data are again viewed as a significant compendium but with the caveat that expert, local knowledge should always complement the information provided.

### Future work

This is the second in a series of three publications describing the distribution and relevant bionomics of the global DVS of malaria. The first publication [2] detailed the DVS of the Americas and the final publication will examine the DVS of the Asian Pacific region (Sinka *et al*: The dominant *Anopheles *vectors of human malaria in the Asia Pacific region: occurrence data, distribution maps and bionomic précis, unpublished). Together, these three publications are intended to provide a baseline set of data and maps and summarise the current knowledge of the bionomics of the 41 DVS identified as the primary vectors of *P. falciparum *and *P. vivax *malaria.

## Conclusions

The maps and data presented here, and those relating to the DVS of the Americas [2], and the Asian Pacific region, will be available on the MAP website [64] in accordance with the open access principles of the MAP (please contact authors for details). These data and maps are provided as a dataset to be improved and built upon. Undoubtedly, the process of species distribution mapping will improve, environmental and climatic spatial data will become available at higher resolutions, and more refined understanding of the ecology that limits a given DVS distribution attained. The single most important factor, however, will be more spatially comprehensive occurrence data and this exercise has been additionally valuable in identifying the paucity of information in large areas in Africa, Europe and the Middle East. An increasing willingness to share data between research groups and national malaria control programmes has been instrumental in this initiative and is critical to its sustained future.

## List of abbreviations

AUC: Area Under the operating characteristic Curve; AVHRR: Advanced Very High Resolution Radiometer; BRT: Boosted Regression Trees; COI: (mitochondrial) Cytochrome Oxidase 1; DEM: Digital Elevation Model; DVS: Dominant Vector Species; EO: Expert Opinion; EVI: Enhanced Vegetation Index; GIS: Geographic Information System; IRS: Insecticide Residual Spraying; ITNs: Insecticide Treated Bednets; ITS2: Internal Transcribed Spacer 2; IVCC: Innovative Vector Control Consortium; LST: Land Surface Temperature; MAP: Malaria Atlas Project; MIR: Middle Infrared Radiation; MODIS: MODerate Resolution Imaging Spectroradiometer; NDVI: Normalized Difference Vegetation Index; PCR: Polymerase Chain Reaction; TAG: Technical Advisory Group; TFA: Temporal Fourier Analysis; WRBU: Walter Reed Biosystematics Unit.

## Competing interests

The authors declare that they have no competing interests.

## Authors' contributions

SIH conceived the study and managed its design and implementation. MES wrote the first draft of the manuscript and assembled the occurrence data with assistance from CWK and RMO, CWK also digitised and edited all the expert opinion maps. WHT designed and maintained the databases and implemented the map figures. APP implemented the BRT scripts for predictive mapping. PWG processed the environmental and climatic data grids, with assistance from TVB. All TAG members (MJB, SM, MC, CMM and JH) provided data and advice in updating the EO range maps. Experiments were devised by SIH and MES and implemented by MES. All authors participated in the interpretation of results and in the writing and editing of the manuscript. MJB, MC, SM, HCJG, CMM and REH advised on bionomics and nomenclature issues, and provided additional comments and input to the manuscript.

## Supplementary Material

Additional file 1 **Expert opinion distribution maps for the seven DVS of Africa and the six DVS of the Europe and Middle Eastern region**. Click here for file

Additional file 2 **Summary tables showing evaluation statistics for all mapping trials and final Boosted Regression Tree environmental and climatic variable selections for the final, optimal predictive maps**. Click here for file

Additional file 3 **Predictive species distribution maps for the seven DVS of Africa and the six DVS of the Europe and Middle Eastern region**. Click here for file
