# Supplementary material for: The dominant Anopheles vectors of human malaria in Africa, Europe and the Middle East: occurrence data, distribution maps and bionomic précis
Source: Parasit Vectors. 2010 Dec 3;3:117. doi: 10.1186/1756-3305-3-117 (PMC3016360; doi:10.1186/1756-3305-3-117)
Supplement: Additional file 2 — Summary tables showing evaluation statistics for all mapping trials and final Boosted Regression Tree environmental and climatic variable selections for the final, optimal predictive maps. [file 1756-3305-3-117-S2.DOC]

**Additional file 2:** Summary tables showing evaluation statistics for all mapping trials and final BRT environmental and climatic variable selection for final, optimal predictive maps.

**Table 2.1**. Environmental and climatic variables grids available to the BRT species mapping listing the abbreviations used in the mapping figures.

| **File name** | **Abbreviation** | **Description** |
| --- | --- | --- |
| Wd0103a0 | MIR (mean) | Middle Infrared (MIR) - mean |
| wd0103a1 | MIR (A1) | Middle Infrared (MIR) - amplitude of the annual cycle |
| wd0103a2 | MIR (A2) | Middle Infrared (MIR) - amplitude of the bi-annual cycle |
| wd0103p1 | MIR (P1) | Middle Infrared (MIR) - phase of the annual cycle |
| wd0103p2 | MIR (P2) | Middle Infrared (MIR) - phase of the bi-annual cycle |
| wd0103mn | MIR (min) | Middle Infrared (MIR) - minimum |
| wd0103mx | MIR (max) | Middle Infrared (MIR) - maximum |
| wd0107a0 | LST (mean) | Land Surface Temperature (LST) - mean |
| wd0107a1 | LST (A1) | Land Surface Temperature (LST) - amplitude of the annual cycle |
| wd0107a2 | LST (A2) | Land Surface Temperature (LST) - amplitude of the bi-annual cycle |
| wd0107p1 | LST (P1) | Land Surface Temperature (LST) - phase of the annual cycle |
| wd0107p2 | LST (P2) | Land Surface Temperature (LST) - phase of the bi-annual cycle |
| wd0107mn | LST (min) | Land Surface Temperature (LST) - minimum |
| wd0107mx | LST (max) | Land Surface Temperature (LST) - maximum |
| wd0114a0 | NDVI (mean) | Normalized Difference Vegetation Index - mean |
| wd0114a1 | NDVI (A1) | Normalized Difference Vegetation Index - amplitude of the annual cycle |
| wd0114a2 | NDVI (A2) | Normalized Difference Vegetation Index - amplitude of the bi-annual cycle |
| wd0114p1 | NDVI (P1) | Normalized Difference Vegetation Index - phase of the annual cycle |
| wd0114p2 | NDVI (P2) | Normalized Difference Vegetation Index - phase of the bi-annual cycle |
| wd0114mn | NDVI (min) | Normalized Difference Vegetation Index - minimum |
| wd0114mx | NDVI (max) | Normalized Difference Vegetation Index - maximum |

**Table 2.1 (cont)**. Environmental and climatic variables grids available to the BRT species mapping listing the abbreviations used in the mapping figures.

| **File name** | **Abbreviation** | **Description** |
| --- | --- | --- |
| mod_dem | DEM | Digital Elevation Model (DEM) |
| mod_lst_a0 | LST (mean) | Land Surface Temperature (LST) - mean |
| mod_lst_a1 | LST (A1) | Land Surface Temperature (LST) - amplitude of the annual cycle |
| mod_lst_a2 | LST (A2) | Land Surface Temperature (LST) - amplitude of the bi-annual cycle |
| mod_lst_p1 | LST (P1) | Land Surface Temperature (LST) - phase of the annual cycle |
| mod_lst_p2 | LST (P2) | Land Surface Temperature (LST) - phase of the bi-annual cycle |
| mod_evi_a0 | EVI (mean) | Enhanced Vegetation Index (LST) - mean |
| mod_evi_a1 | EVI (A1) | Enhanced Vegetation Index (LST) - amplitude of the annual cycle |
| mod_evi_a2 | EVI (A2) | Enhanced Vegetation Index (LST) - amplitude of the bi-annual cycle |
| mod_evi_p1 | EVI (P1) | Enhanced Vegetation Index (LST) - phase of the annual cycle |
| mod_evi_p2 | EVI (P2) | Enhanced Vegetation Index (LST) - phase of the bi-annual cycle |
| prec57a0 | Prec (mean) | Precipitation - mean |
| prec57a1 | Prec (A1) | Precipitation - amplitude of the annual cycle |
| prec57a2 | Prec (A2) | Precipitation - amplitude of the bi-annual cycle |
| prec57mn | Prec (min) | Precipitation - minimum |
| prec57mx | Prec (max) | Precipitation - maximum |
| prec57p1 | Prec (P1) | Precipitation - phase of the annual cycle |
| prec57p2 | Prec (P2) | Precipitation - phase of the bi-annual cycle |
| globcover5k | GLOB (ch. no.) | See table 3.2 |
| gc5k_dry | GLOB (dry) | Globcover – dry land cover classes [140, 150, 200] – see table 3.2 |
| gc5k_flo | GLOB (flood) | Globcover – flooded land cover classes [160, 170, 180] – see table 3.2 |
| gc5k_frs | GLOB (forest) | Globcover – forested land cover classes [40, 50, 60, 90, 100] – see table 3.2 |

**Table 2.2.** Globcover channels (land cover classes) available to the BRT species mapping (Channels 210: water bodies; 220: Permanent snow and ice and 230: no data were not included in the modelling).

| **Channel** | **Description** |
| --- | --- |
| 11 | Post-flooding or irrigated croplands (or aquatic) |
| 14 | Rainfed croplands |
| 20 | Mosaic cropland (50-70%)/vegetation (grassland/shrubland/forest) (20-50%) |
| 30 | Mosaic vegetation (grassland/shrubland/forest) (50-70%)/cropland (20-50%) |
| 40 | Closed to open (>15%) broadleaved evergreen or semi-deciduous forest (>5m) |
| 50 | Closed (>40%) broadleaved deciduous forest (>5m) |
| 60 | Open (15-40%) broadleaved deciduous forest/woodland (>5m) |
| 70 | Closed (>40%) needleleaved evergreen forest (>5m) |
| 90 | Open (15-40%) needleleaved deciduous or evergreen forest (>5m) |
| 100 | Closed to open (>15%) mixed broadleaved and needleleaved forest (>5m) |
| 110 | Mosaic forest or shrubland (50-70%) / grassland (20-50%) |
| 120 | Mosaic grassland (50-70%) / forest or shrubland (20-50%) |
| 130 | Closed to open (>15%) (broadleaved or needleleaved, evergreen or deciduous) shrubland (<5m) |
| 140 | Closed to open (>15%) herbaceous vegetation (grassland, savannas or lichens/mosses) |
| 150 | Sparse (<15%) vegetation |
| 160 | Closed to open (>15%) broadleaved forest regularly flooded (semi-permanently or temporarily) - Fresh or brackish water |
| 170 | Closed (>40%) broadleaved forest or shrubland permanently flooded - Saline or brackish water |
| 180 | Closed to open (>15%) grassland or woody vegetation on regularly flooded or waterlogged soil - Fresh, brackish or saline water |
| 190 | Artificial surfaces and associated areas (Urban areas >50%) |
| 200 | Bare areas |

**Table 2.3**: Evaluation statistics and the top five environmental/climatic variables selected by the BRT for the seven DVS in Africa using a combination of data and 500 pseudo-presences generated within the EO range, but given a weight rating of half the true data (‘hybrid’), and 10:1 pseudo-absence:presence generated from within a 1500 km buffer area.

| Species | Evaluation | | Environmental variables |
| --- | --- | --- | --- |
| *An. arabiensis* (1196) | Deviance: | 0.094 | | 1 | NDVI (P1) | | --- | --- | | 2 | Prec (A2) | | 3 | LST (P1) | | 4 | Prec (max) | | 5 | MIR (P1) | |
|  | Correlation: | 0.926 |  |
|  | Discrimination (AUC): | 0.992 |  |
|  | Kappa: | 0.907 |  |
| *An. funestus* (919) | Deviance: | 0.062 | | 1 | Prec (max) | | --- | --- | | 2 | NDVI (mean) | | 3 | Prec (A2) | | 4 | MIR (mean) | | 5 | NDVI (A1) | |
|  | Correlation: | 0.954 |  |
|  | Discrimination (AUC): | 0.998 |  |
|  | Kappa: | 0.944 |  |
| *An. gambiae* (1443) | Deviance: | 0.114 | | 1 | Prec (mean) | | --- | --- | | 2 | Prec (max) | | 3 | DEM | | 4 | Prec (A2) | | 5 | LST (min) | |
|  | Correlation: | 0.920 |  |
|  | Discrimination (AUC): | 0.989 |  |
|  | Kappa: | 0.900 |  |
| *An. melas* (149) | Deviance: | 0.187 | | 1 | DEM | | --- | --- | | 2 | LST (max) | | 3 | Prec (P1) | | 4 | Prec (max) | | 5 | LST (mean) | |
|  | Correlation: | 0.907 |  |
|  | Discrimination (AUC): | 0.989 |  |
|  | Kappa: | 0.869 |  |
| *An. merus* (73) | Deviance: | 0.240 | | 1 | DEM | | --- | --- | | 2 | LST (A1) | | 3 | Prec (P2) | | 4 | MIR (P1) | | 5 | NDVI (P2) | |
|  | Correlation: | 0.901 |  |
|  | Discrimination (AUC): | 0.983 |  |
|  | Kappa: | 0.876 |  |
| *An. moucheti* (66) | Deviance: | 0.225 | | 1 | Prec (mean) | | --- | --- | | 2 | MIR (mean) | | 3 | LST (min) | | 4 | LST (mean) | | 5 | LST (P2) | |
|  | Correlation: | 0.908 |  |
|  | Discrimination (AUC): | 0.986 |  |
|  | Kappa: | 0.881 |  |
| *An. nili* (105) | Deviance: | 0.213 | | 1 | Prec (max) | | --- | --- | | 2 | GLOB (dry) | | 3 | NDVI (max) | | 4 | LST (min) | | 5 | NDVI (A2) | |
|  | Correlation: | 0.908 |  |
|  | Discrimination (AUC): | 0.985 |  |
|  | Kappa: | 0.889 |  |

**Table 2.4**: Evaluation metrics of mapping trials of data only maps (‘data’); expert opinion maps where 500 pseudo-presences were generated randomly within the EO range (‘EO’); and a combination of data and 500 pseudo-presences generated within the EO range, but given a weight rating of half the true data (‘hybrid’). All maps were run using a 1000 km buffer against 1000 pseudo-absences at 5 x 5 km resolution.

|  |  | Metrics | | | |
| --- | --- | --- | --- | --- | --- |
| Species (no. of presence data) | Trial | Deviance  (0-1) | Correlation  (0-1) | Discrimination (AUC)  (0-1) | Kappa (κ)  (-1 to 1) |
| *An. arabiensis* (1196) | **data** | 0.135 | 0.965 | 0.996 | 0.958 |
|  | **EO** | 0.350 | 0.890 | 0.982 | 0.857 |
|  | **hybrid** | 0.189 | 0.925 | 0.989 | 0.899 |
| *An. funestus* (919) | **data** | 0.078 | 0.981 | 0.998 | 0.977 |
|  | **EO** | 0.196 | 0.940 | 0.994 | 0.918 |
|  | **hybrid** | 0.097 | 0.964 | 0.997 | 0.956 |
| *An. gambiae* (1443) | **data** | 0.191 | 0.954 | 0.992 | 0.939 |
|  | **EO** | 0.409 | 0.876 | 0.974 | 0.836 |
|  | **hybrid** | 0.219 | 0.918 | 0.986 | 0.899 |
| *An. melas* (149) | **data** | 0.049 | 0.968 | 0.999 | 0.961 |
|  | **EO** | 0.293 | 0.915 | 0.986 | 0.896 |
|  | **hybrid** | 0.203 | 0.918 | 0.988 | 0.891 |
| *An. merus* (73) | **data** | 0.156 | 0.846 | 0.975 | 0.811 |
|  | **EO** | 0.288 | 0.918 | 0.986 | 0.899 |
|  | **hybrid** | 0.275 | 0.891 | 0.976 | 0.868 |
| *An. moucheti* (66) | **data** | 0.062 | 0.938 | 0.994 | 0.922 |
|  | **EO** | 0.270 | 0.915 | 0.989 | 0.888 |
|  | **hybrid** | 0.208 | 0.897 | 0.987 | 0.865 |
| *An. nili* (105) | **data** | 0.080 | 0.944 | 0.994 | 0.921 |
|  | **EO** | 0.332 | 0.897 | 0.982 | 0.862 |
|  | **hybrid** | 0.222 | 0.900 | 0.986 | 0.874 |

**Table 2.5**: Evaluation statistics for a range of buffer sizes. All maps were run using ‘hybrid’ data (a combination of data and 500 pseudo-presences generated within the EO range, but given a weight rating of half the true data) against 1000 pseudo-absences at 5 x 5 km resolution.

|  |  | Metrics | | | |
| --- | --- | --- | --- | --- | --- |
| Species (no. of presence data) | Buffer size (km) | Deviance  (0-1) | Correlation  (0-1) | Discrimination (AUC)  (0-1) | Kappa (κ)  (-1 to 1) |
| ***An. arabiensis* (1196)** | **100** | 0.320 | 0.869 | 0.974 | 0.837 |
|  | **500** | 0.217 | 0.915 | 0.987 | 0.893 |
|  | **1000** | 0.189 | 0.925 | 0.989 | 0.899 |
|  | **1500** | 0.173 | 0.932 | 0.992 | 0.915 |
| ***An. funestus* (919)** | **100** | 0.253 | 0.908 | 0.983 | 0.890 |
|  | **500** | 0.144 | 0.953 | 0.993 | 0.941 |
|  | **1000** | 0.097 | 0.964 | 0.997 | 0.956 |
|  | **1500** | 0.116 | 0.957 | 0.997 | 0.945 |
| ***An. gambiae* (1443)** | **100** | 0.374 | 0.855 | 0.967 | 0.829 |
|  | **500** | 0.262 | 0.902 | 0.981 | 0.890 |
|  | **1000** | 0.219 | 0.918 | 0.986 | 0.899 |
|  | **1500** | 0.201 | 0.923 | 0.988 | 0.901 |
| ***An. melas* (149)** | **100** | 0.506 | 0.735 | 0.914 | 0.673 |
|  | **500** | 0.315 | 0.854 | 0.970 | 0.815 |
|  | **1000** | 0.203 | 0.918 | 0.988 | 0.891 |
|  | **1500** | 0.183 | 0.924 | 0.990 | 0.901 |
| ***An. merus* (73)** | **100** | 0.483 | 0.747 | 0.921 | 0.672 |
|  | **500** | 0.292 | 0.876 | 0.974 | 0.848 |
|  | **1000** | 0.275 | 0.891 | 0.976 | 0.868 |
|  | **1500** | 0.212 | 0.912 | 0.987 | 0.887 |

**Table 2.5:** (cont.) Evaluation statistics for a range of buffer sizes. All maps were run using ‘hybrid’ data (a combination of data and 500 pseudo-presences generated within the EO range, but given a weight rating of half the true data) against 1000 pseudo-absences at 5 x 5 km resolution.

|  |  | Metrics | | | |
| --- | --- | --- | --- | --- | --- |
| Species | Buffer size (km) | Deviance  (0-1) | Correlation  (0-1) | Discrimination (AUC)  (0-1) | Kappa (κ)  (-1 to 1) |
| *An. moucheti* (66) | **100** | 0.251 | 0.877 | 0.964 | 0.843 |
|  | **500** | 0.231 | 0.889 | 0.983 | 0.853 |
|  | **1000** | 0.208 | 0.897 | 0.987 | 0.865 |
|  | **1500** | 0.204 | 0.903 | 0.988 | 0.866 |
| *An. nili* (105) | **100** | 0.328 | 0.846 | 0.956 | 0.815 |
|  | **500** | 0.210 | 0.903 | 0.983 | 0.868 |
|  | **1000** | 0.222 | 0.900 | 0.986 | 0.874 |
|  | **1500** | 0.216 | 0.903 | 0.985 | 0.870 |

**Table 2.6**: Evaluation statistics for a range of pseudo-absence:occurrence data ratios and constant values. All maps were run at a 5 x 5 km resolution using ‘hybrid’ data (a combination of data and 500 pseudo-presences generated within the EO range, but given a weight rating of half the true data) with the pseudo-absences taken from within a 1000km buffer.

|  |  | Metrics | | | |
| --- | --- | --- | --- | --- | --- |
| Species (no. of presence data) | Pseudo-absence:presence | Deviance  (0-1) | Correlation  (0-1) | Discrimination  (AUC: 0-1) | Kappa (κ)  (-1 to 1) |
| *An. arabiensis* (1196) | **1:1** | 0.179 | 0.933 | 0.992 | 0.915 |
|  | **2:1** | 0.172 | 0.936 | 0.992 | 0.917 |
|  | **5:1** | 0.128 | 0.937 | 0.991 | 0.925 |
|  | **10:1** | 0.099 | 0.925 | 0.989 | 0.908 |
|  | **500 points** | 0.207 | 0.888 | 0.985 | 0.851 |
|  | **1000 points** | 0.195 | 0.921 | 0.989 | 0.899 |
| *An. funestus* (919) | **1:1** | 0.120 | 0.958 | 0.994 | 0.947 |
|  | **2:1** | 0.121 | 0.959 | 0.994 | 0.953 |
|  | **5:1** | 0.091 | 0.955 | 0.997 | 0.943 |
|  | **10:1** | 0.073 | 0.949 | 0.995 | 0.941 |
|  | **500 points** | 0.088 | 0.964 | 0.997 | 0.953 |
|  | **1000 points** | 0.107 | 0.964 | 0.996 | 0.960 |
| *An. gambiae* (1443) | **1:1** | 0.266 | 0.913 | 0.981 | 0.894 |
|  | **2:1** | 0.267 | 0.912 | 0.982 | 0.891 |
|  | **5:1** | 0.156 | 0.928 | 0.987 | 0.916 |
|  | **10:1** | 0.133 | 0.904 | 0.985 | 0.884 |
|  | **500 points** | 0.173 | 0.907 | 0.989 | 0.885 |
|  | **1000 points** | 0.227 | 0.919 | 0.985 | 0.905 |
| *An. melas* (149) | **1:1** | 0.165 | 0.909 | 0.984 | 0.884 |
|  | **2:1** | 0.153 | 0.912 | 0.987 | 0.897 |
|  | **5:1** | 0.220 | 0.907 | 0.987 | 0.874 |
|  | **10:1** | 0.220 | 0.895 | 0.982 | 0.865 |
|  | **500 points** | 0.293 | 0.892 | 0.974 | 0.866 |
|  | **1000 points** | 0.250 | 0.887 | 0.982 | 0.841 |

**Table 2.6:** (cont.) Evaluation statistics for a range of pseudo-absence:occurrence data ratios and constant values. All maps were run at a 5 x 5 km resolution using ‘hybrid’ data (a combination of data and 500 pseudo-presences generated within the EO range, but given a weight rating of half the true data) with the pseudo-absences taken from within a 1000km buffer.

|  |  | Metrics | | | |
| --- | --- | --- | --- | --- | --- |
| Species (no. of presence data) | Pseudo-absence:presence | Deviance  (0-1) | Correlation  (0-1) | Discrimination (AUC: 0-1) | Kappa (κ)  (-1 to 1) |
| *An. merus* (73) | **1:1** | 0.161 | 0.863 | 0.978 | 0.843 |
|  | **2:1** | 0.171 | 0.849 | 0.969 | 0.824 |
|  | **5:1** | 0.275 | 0.897 | 0.978 | 0.874 |
|  | **10:1** | 0.255 | 0.897 | 0.983 | 0.863 |
|  | **500 points** | 0.271 | 0.898 | 0.981 | 0.873 |
|  | **1000 points** | 0.277 | 0.885 | 0.978 | 0.857 |
| *An. moucheti* (66) | **1:1** | 0.140 | 0.886 | 0.981 | 0.868 |
|  | **2:1** | 0.164 | 0.869 | 0.974 | 0.846 |
|  | **5:1** | 0.239 | 0.890 | 0.984 | 0.840 |
|  | **10:1** | 0.191 | 0.917 | 0.988 | 0.894 |
|  | **500 points** | 0.215 | 0.903 | 0.988 | 0.865 |
|  | **1000 points** | 0.219 | 0.896 | 0.986 | 0.865 |
| *An. nili* (105) | **1:1** | 0.221 | 0.823 | 0.976 | 0.750 |
|  | **2:1** | 0.216 | 0.823 | 0.976 | 0.752 |
|  | **5:1** | 0.254 | 0.893 | 0.982 | 0.863 |
|  | **10:1** | 0.220 | 0.893 | 0.986 | 0.857 |
|  | **500 points** | 0.239 | 0.906 | 0.984 | 0.885 |
|  | **1000 points** | 0.213 | 0.902 | 0.987 | 0.872 |

**Table 2.7**: Evaluation statistics and the top five environmental/climatic variables selected by the BRT for the six DVS in Europe and the Middle East using a combination of data and 500 pseudo-presences generated within the EO range, but given a weight rating of half the true data (‘hybrid’), and 10:1 pseudo-absence:presence generated from within a 1000 km buffer area. *Anopheles atroparvus* and *An. messeae* maps were run using MODIS environmental variables instead of AVHRR (see main text).

| Species | Evaluation | | Environmental variables |
| --- | --- | --- | --- |
| *An. atroparvus* (1044) | Deviance: | 0.157 | | 1 | GLOB (190) | | --- | --- | | 2 | Prec (min) | | 3 | EVI (mean) | | 4 | EVI (P2) | | 5 | LST (P1) | |
|  | Correlation: | 0.888 |  |
|  | Discrimination (AUC): | 0.984 |  |
|  | Kappa: | 0.859 |  |
| *An. labranchiae* (234) | Deviance: | 0.269 | | 1 | Prec (P2) | | --- | --- | | 2 | GLOB (190) | | 3 | Prec (A2) | | 4 | NDVI (min) | | 5 | MIR (P1) | |
|  | Correlation: | 0.832 |  |
|  | Discrimination (AUC): | 0.971 |  |
|  | Kappa: | 0.781 |  |
| *An. messeae* (903) | Deviance: | 0.208 | | 1 | Prec (min) | | --- | --- | | 2 | Prec (P2) | | 3 | GLOB (190) | | 4 | DEM | | 5 | EVI (mean) | |
|  | Correlation: | 0.829 |  |
|  | Discrimination (AUC): | 0.967 |  |
|  | Kappa: | 0.785 |  |
| *An. sacharovi* (183) | Deviance: | 0.366 | | 1 | Prec (A1) | | --- | --- | | 2 | MIR (P2) | | 3 | DEM | | 4 | Prec (max) | | 5 | LST (min) | |
|  | Correlation: | 0.792 |  |
|  | Discrimination (AUC): | 0.957 |  |
|  | Kappa: | 0.726 |  |
| *An. sergentii* (35) | Deviance: | 0.437 | | 1 | LST (min) | | --- | --- | | 2 | Prec (P1) | | 3 | Prec (P2) | | 4 | Prec (A2) | | 5 | DEM | |
|  | Correlation: | 0.792 |  |
|  | Discrimination (AUC): | 0.942 |  |
|  | Kappa: | 0.730 |  |
| *An superpictus* (385) | Deviance: | 0.290 | | 1 | GLOB (190) | | --- | --- | | 2 | Prec (P1) | | 3 | Prec (P2) | | 4 | LST (min) | | 5 | NDVI (min) | |
|  | Correlation: | 0.797 |  |
|  | Discrimination (AUC): | 0.964 |  |
|  | Kappa: | 0.728 |  |

**Table 2.8**: Evaluation metrics of mapping trials of data only maps (‘data’); expert opinion maps where 500 pseudo-presences were generated randomly within the EO range (‘EO’); and a combination of data and 500 pseudo-presences generated within the EO range, but given a weight rating of half the true data (‘hybrid’). All maps were run using a 1000 km buffer against 1000 pseudo-absences at 5 x 5 km resolution.

|  |  | Metrics | | | |
| --- | --- | --- | --- | --- | --- |
| Species (no. of presence data) | Trial | Deviance  (0-1) | Correlation  (0-1) | Discrimination (AUC)  (0-1) | Kappa (κ)  (-1 to 1) |
| *An. atroparvus* (1044) | **data** | 0.333 | 0.901 | 0.983 | 0.874 |
|  | **EO** | 0.331 | 0.897 | 0.983 | 0.864 |
|  | **hybrid** | 0.305 | 0.891 | 0.981 | 0.859 |
| *An. labranchiae* (234) | **data** | 0.211 | 0.913 | 0.986 | 0.889 |
|  | **EO** | 0.368 | 0.889 | 0.978 | 0.866 |
|  | **hybrid** | 0.293 | 0.868 | 0.977 | 0.825 |
| *An. messeae* (903) | **data** | 0.296 | 0.915 | 0.985 | 0.893 |
|  | **EO** | 0.538 | 0.818 | 0.957 | 0.770 |
|  | **hybrid** | 0.388 | 0.835 | 0.965 | 0.788 |
| *An. sacharovi* (183) | **data** | 0.295 | 0.827 | 0.974 | 0.791 |
|  | **EO** | 0.457 | 0.856 | 0.968 | 0.821 |
|  | **hybrid** | 0.400 | 0.823 | 0.960 | 0.762 |
| *An. sergentii* (35) | **data** | 0.119 | 0.769 | 0.954 | 0.695 |
|  | **EO** | 0.568 | 0.816 | 0.951 | 0.778 |
|  | **hybrid** | 0.474 | 0.734 | 0.927 | 0.647 |
| *An superpictus* (385) | **data** | 0.301 | 0.894 | 0.979 | 0.865 |
|  | **EO** | 0.446 | 0.859 | 0.968 | 0.820 |
|  | **hybrid** | 0.440 | 0.821 | 0.954 | 0.770 |

**Table 2.9**: Evaluation statistics for a range of buffer sizes. All maps were run using ‘hybrid’ data (a combination of data and 500 pseudo-presences generated within the EO range, but given a weight rating of half the true data) against 1000 pseudo-absences at 5 x 5 km resolution.

|  |  | Metrics | | | |
| --- | --- | --- | --- | --- | --- |
| Species (no. of presence data) | Buffer size (km) | Deviance  (0-1) | Correlation  (0-1) | Discrimination (AUC)  (0-1) | Kappa (κ)  (-1 to 1) |
| *An. atroparvus* (1044) | **100** | 0.513 | 0.803 | 0.947 | 0.769 |
|  | **500** | 0.522 | 0.799 | 0.945 | 0.765 |
|  | **1000** | 0.305 | 0.891 | 0.981 | 0.859 |
|  | **1500** | 0.518 | 0.799 | 0.946 | 0.763 |
| *An. labranchiae* (234) | **100** | 0.552 | 0.723 | 0.910 | 0.659 |
|  | **500** | 0.553 | 0.722 | 0.911 | 0.663 |
|  | **1000** | 0.293 | 0.868 | 0.977 | 0.825 |
|  | **1500** | 0.556 | 0.722 | 0.910 | 0.660 |
| *An. messeae* (903) | **100** | 0.648 | 0.702 | 0.901 | 0.655 |
|  | **500** | 0.651 | 0.698 | 0.898 | 0.659 |
|  | **1000** | 0.388 | 0.835 | 0.965 | 0.788 |
|  | **1500** | 0.648 | 0.701 | 0.899 | 0.661 |
| *An. sacharovi* (183) | **100** | 0.632 | 0.682 | 0.887 | 0.598 |
|  | **500** | 0.643 | 0.674 | 0.884 | 0.595 |
|  | **1000** | 0.400 | 0.823 | 0.960 | 0.762 |
|  | **1500** | 0.640 | 0.675 | 0.883 | 0.593 |
| *An. sergentii* (35) | **100** | 0.500 | 0.711 | 0.895 | 0.632 |
|  | **500** | 0.498 | 0.714 | 0.895 | 0.636 |
|  | **1000** | 0.474 | 0.734 | 0.927 | 0.647 |
|  | **1500** | 0.501 | 0.713 | 0.895 | 0.627 |
| *An superpictus* (385) | **100** | 0.659 | 0.701 | 0.897 | 0.631 |
|  | **500** | 0.655 | 0.703 | 0.898 | 0.636 |
|  | **1000** | 0.440 | 0.821 | 0.954 | 0.770 |
|  | **1500** | 0.653 | 0.705 | 0.899 | 0.645 |

**Table 2.10**: Evaluation statistics for a range of pseudo-absence:occurrence data ratios and constant values. All maps were run at a 5 x 5 km resolution using ‘hybrid’ data (a combination of data and 500 pseudo-presences generated within the EO range, but given a weight rating of half the true data) with the pseudo-absences taken from within a 1000km buffer.

|  |  | Metrics | | | |
| --- | --- | --- | --- | --- | --- |
| Species (no. of presence data) | Pseudo-absence:presence | Deviance  (0-1) | Correlation  (0-1) | Discrimination  (AUC: 0-1) | Kappa (κ)  (-1 to 1) |
| *An. atroparvus* (1044) | **1:1** | 0.329 | 0.882 | 0.977 | 0.848 |
|  | **2:1** | 0.287 | 0.901 | 0.983 | 0.881 |
|  | **5:1** | 0.239 | 0.887 | 0.981 | 0.858 |
|  | **10:1** | 0.157 | 0.888 | 0.984 | 0.859 |
|  | **500 points** | 0.288 | 0.867 | 0.978 | 0.834 |
|  | **1000 points** | 0.305 | 0.891 | 0.981 | 0.859 |
| *An. labranchiae* (234) | **1:1** | 0.262 | 0.870 | 0.972 | 0.824 |
|  | **2:1** | 0.316 | 0.875 | 0.972 | 0.848 |
|  | **5:1** | 0.279 | 0.877 | 0.978 | 0.837 |
|  | **10:1** | 0.269 | 0.832 | 0.971 | 0.781 |
|  | **500 points** | 0.291 | 0.884 | 0.975 | 0.865 |
|  | **1000 points** | 0.293 | 0.868 | 0.977 | 0.825 |
| *An. messeae* (903) | **1:1** | 0.417 | 0.817 | 0.957 | 0.765 |
|  | **2:1** | 0.386 | 0.838 | 0.963 | 0.800 |
|  | **5:1** | 0.330 | 0.816 | 0.955 | 0.771 |
|  | **10:1** | 0.208 | 0.829 | 0.967 | 0.785 |
|  | **500 points** | 0.401 | 0.786 | 0.951 | 0.716 |
|  | **1000 points** | 0.388 | 0.835 | 0.965 | 0.788 |
| *An. sacharovi* (183) | **1:1** | 0.346 | 0.811 | 0.946 | 0.778 |
|  | **2:1** | 0.445 | 0.805 | 0.944 | 0.762 |
|  | **5:1** | 0.426 | 0.807 | 0.953 | 0.745 |
|  | **10:1** | 0.366 | 0.792 | 0.957 | 0.726 |
|  | **500 points** | 0.379 | 0.837 | 0.964 | 0.793 |
|  | **1000 points** | 0.400 | 0.823 | 0.960 | 0.762 |

**Table 2.10:** (cont.) Evaluation statistics for a range of pseudo-absence:occurrence data ratios and constant values. All maps were run at a 5 x 5 km resolution using ‘hybrid’ data (a combination of data and 500 pseudo-presences generated within the EO range, but given a weight rating of half the true data) with the pseudo-absences taken from within a 1000km buffer.

|  |  | Metrics | | | |
| --- | --- | --- | --- | --- | --- |
| Species (no. of presence data) | Pseudo-absence:presence | Deviance  (0-1) | Correlation  (0-1) | Discrimination (AUC: 0-1) | Kappa (κ)  (-1 to 1) |
| *An. sergentii* (35) | **1:1** | 0.220 | 0.617 | 0.915 | 0.512 |
|  | **2:1** | 0.367 | 0.615 | 0.879 | 0.525 |
|  | **5:1** | 0.411 | 0.773 | 0.929 | 0.750 |
|  | **10:1** | 0.437 | 0.792 | 0.942 | 0.730 |
|  | **500 points** | 0.417 | 0.810 | 0.952 | 0.759 |
|  | **1000 points** | 0.474 | 0.734 | 0.927 | 0.647 |
| *An superpictus* (385) | **1:1** | 0.392 | 0.828 | 0.951 | 0.806 |
|  | **2:1** | 0.425 | 0.833 | 0.956 | 0.795 |
|  | **5:1** | 0.346 | 0.832 | 0.967 | 0.783 |
|  | **10:1** | 0.290 | 0.797 | 0.964 | 0.728 |
|  | **500 points** | 0.390 | 0.834 | 0.961 | 0.790 |
|  | **1000 points** | 0.440 | 0.821 | 0.954 | 0.770 |
