# Supplementary material for: The dominant Anopheles vectors of human malaria in Africa, Europe and the Middle East: occurrence data, distribution maps and bionomic précis
Source: Parasit Vectors. 2010 Dec 3;3:117. doi: 10.1186/1756-3305-3-117 (PMC3016360; doi:10.1186/1756-3305-3-117)

Anopheles (*Cellia*) *arabiensis* Patton, 1905

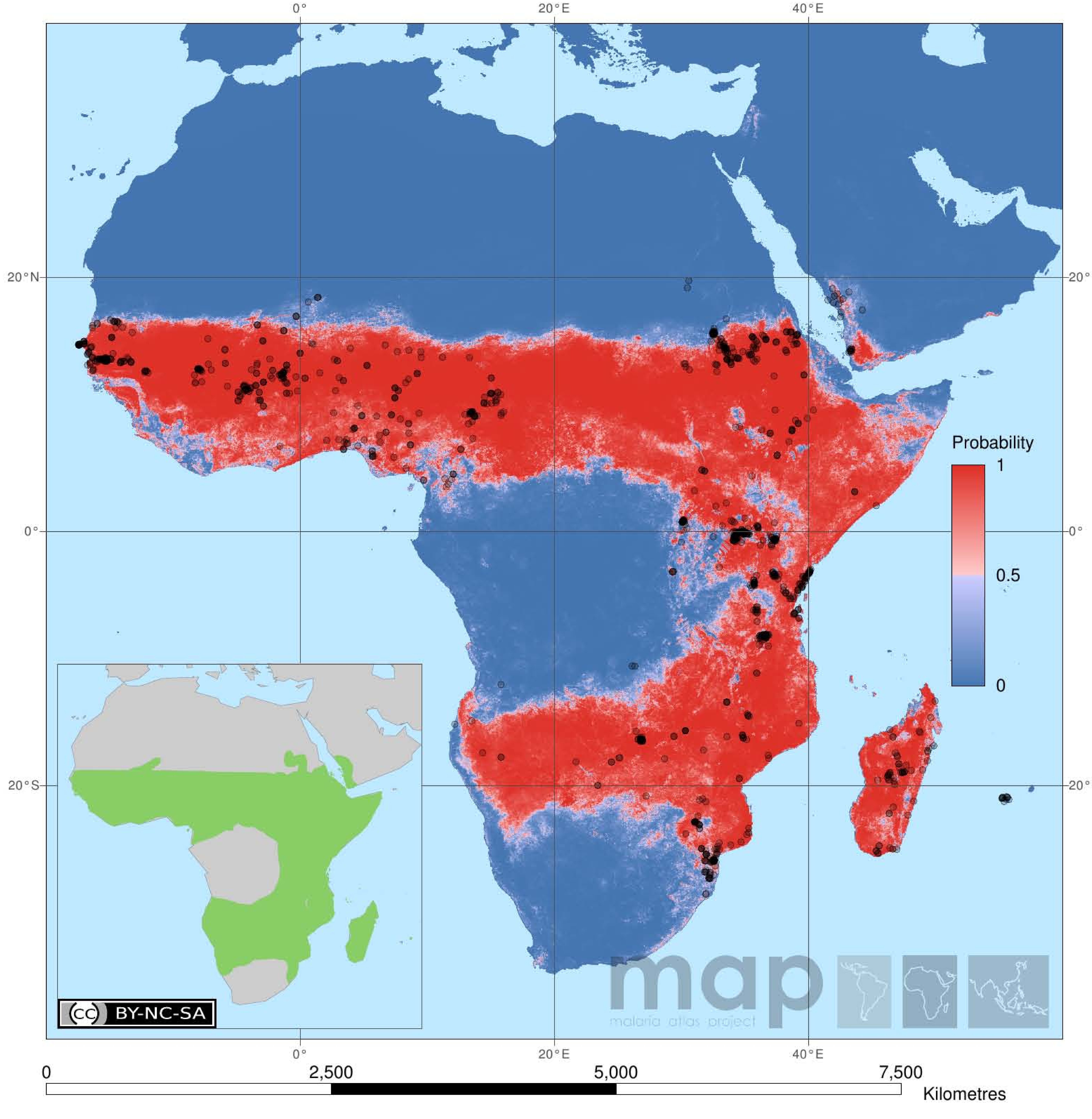

Mapping details: This map shows the predicted probability of occurrence of *An. arabiensis* in Africa. The map was created with the Boosted Regression Trees (BRT) technique using 1,196 occurrence points, 500 pseudo occurrence points generated from a stratified random sample within the expert opinion range (see inset), balanced by 11,960 pseudo absence points sampled within a 1,500 km buffer outside the expert opinion range. The pseudo presence data were given half the weight of observed occurrence data. Predictions are not shown beyond the 1,500 km buffer. The black dots show 1,196 records of occurrence for *An. arabiensis* as detailed in Hay *et al.* [1].

Map statistics: Deviance=0.0941, Correlation=0.9257, Discrimination (AUC)=0.9922, Kappa=0.9071.

Environmental variables used: 1. NDVI (P1), 2. Prec (A2), 3. LST (P1), 4. Prec (max) and 5. MIR (P1). Please see additional file 2 for abbreviations and definitions.

Copyright: Licensed to the Malaria Atlas Project (MAP; www.map.ox.ac.uk) under a Creative Commons Attribution 3.0 License (http://creativecommons.org/).

Citation: Sinka *et al.* (2010). The dominant *Anopheles* vectors of human malaria in Africa, Europe and the Middle East: occurrence data, distribution maps and bionomic précis. *Parasites & Vectors*, **3**:117.

Anopheles (*Cellia*) *funestus* Giles, 1900

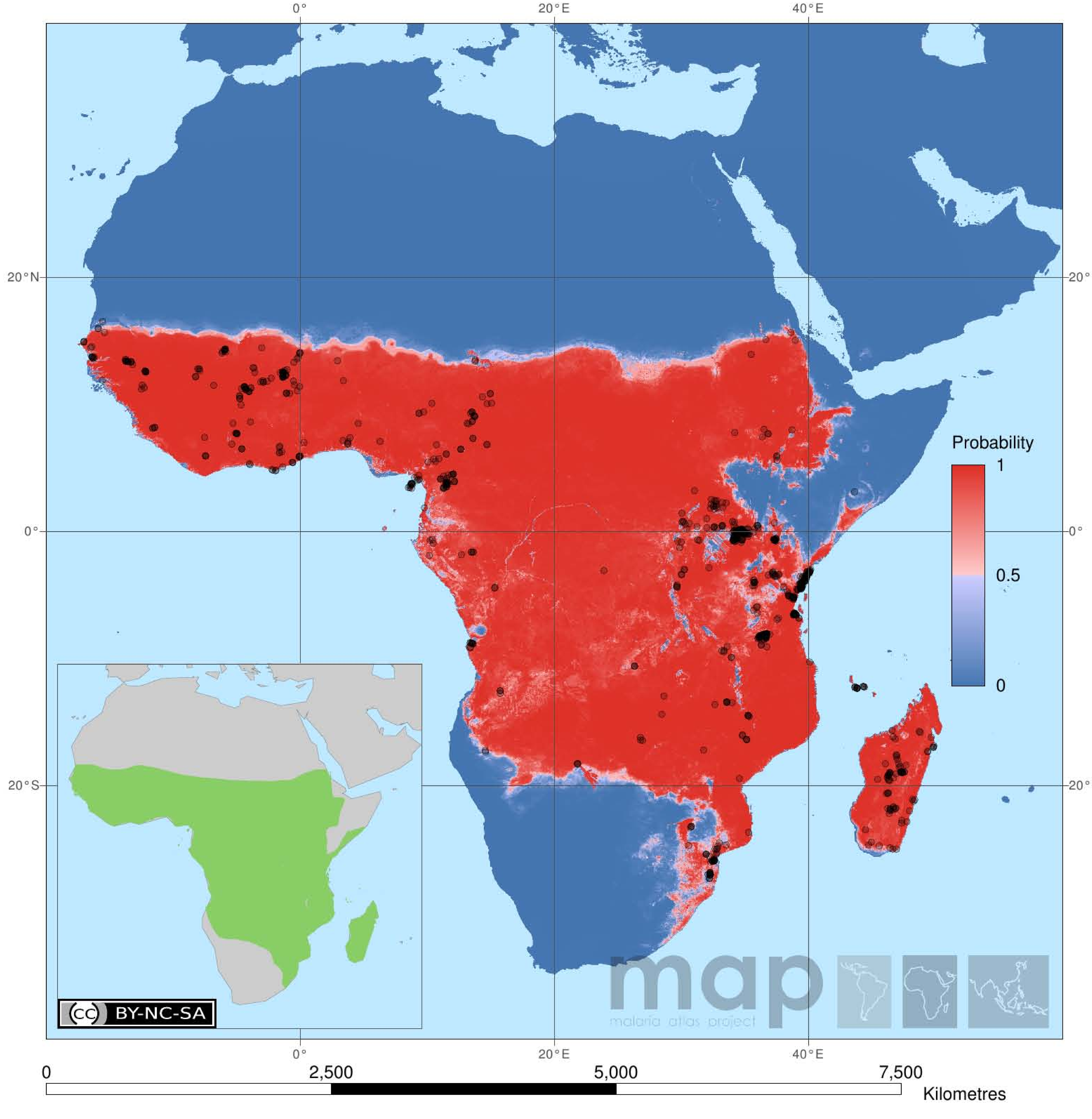

Mapping details: This map shows the predicted probability of occurrence of *An. funestus* in Africa. The map was created with the Boosted Regression Trees (BRT) technique using 919 occurrence points, 500 pseudo occurrence points generated from a stratified random sample within the expert opinion range (see inset), balanced by 9,190 pseudo absence points sampled within a 1,500 km buffer outside the expert opinion range. The pseudo presence data were given half the weight of observed occurrence data. Predictions are not shown beyond the 1,500 km buffer. The black dots show 919 records of occurrence for *An. funestus* as detailed in Hay *et al.* [1].

Map statistics: Deviance=0.0618, Correlation=0.9539, Discrimination (AUC)=0.9977, Kappa=0.9441.

Environmental variables used: 1. Prec (max), 2. NDVI (mean), 3. Prec (A2), 4. MIR (mean) and 5. NDVI (A1). Please see additional file 2 for abbreviations and definitions.

Copyright: Licensed to the Malaria Atlas Project (MAP; www.map.ox.ac.uk) under a Creative Commons Attribution 3.0 License (http://creativecommons.org/).

Citation: Sinka *et al.* (2010). The dominant *Anopheles* vectors of human malaria in Africa, Europe and the Middle East: occurrence data, distribution maps and bionomic précis. *Parasites & Vectors*, **3**:117.

Anopheles (*Cellia*) *gambiae* Giles, 1902

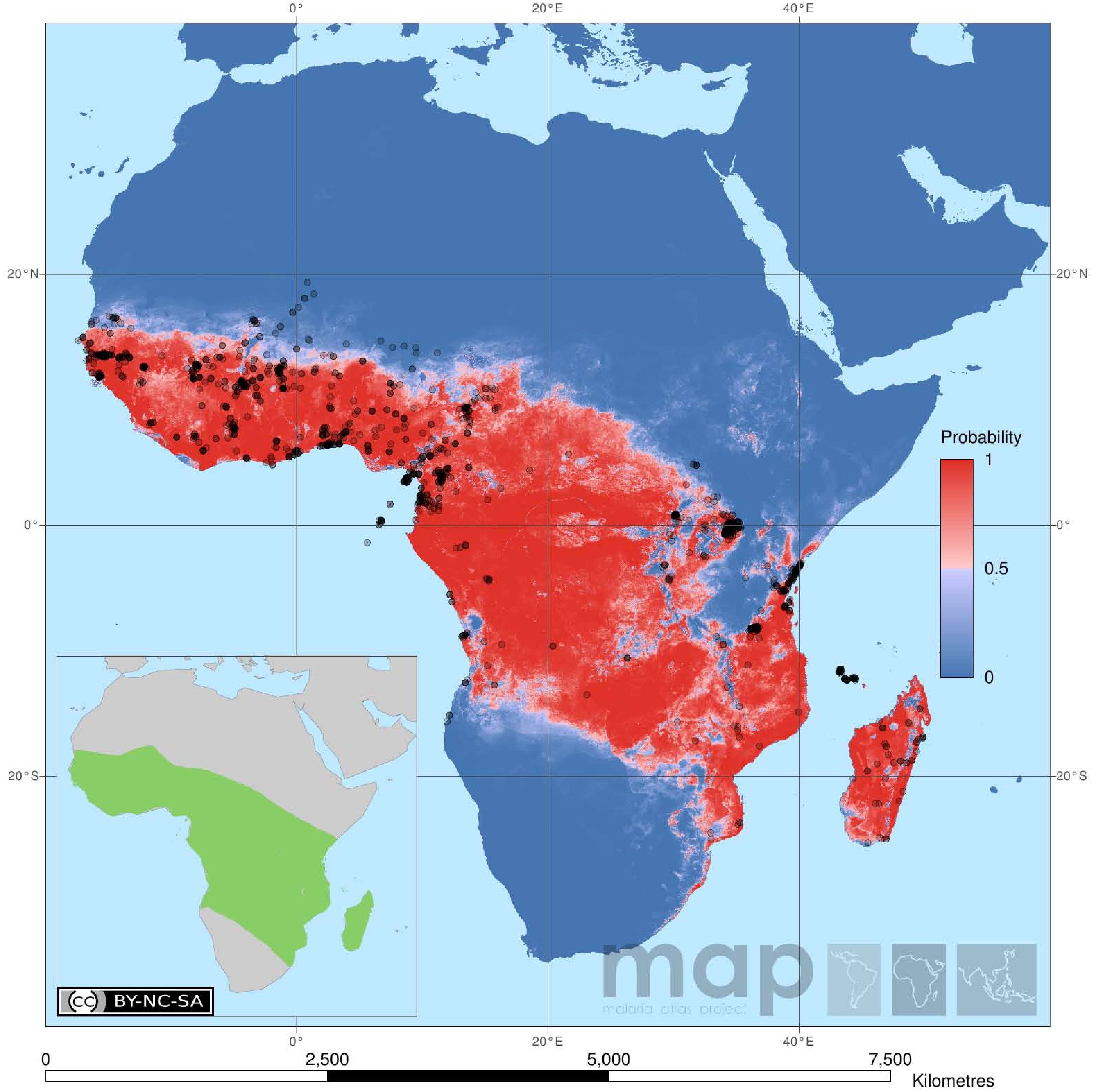

N

Mapping details: This map shows the predicted probability of occurrence of *An. gambiae* in Africa. The map was created with the Boosted Regression Trees (BRT) technique using 1,443 occurrence points, 500 pseudo occurrence points generated from a stratified random sample within the expert opinion range (see inset), balanced by 14,430 pseudo absence points sampled within a 1,500 km buffer outside the expert opinion range. The pseudo presence data were given half the weight of observed occurrence data. Predictions are not shown beyond the 1,500 km buffer. The black dots show 1,443 records of occurrence for *An. gambiae* as detailed in Hay *et al.* [1].

Map statistics: Deviance=0.114, Correlation=0.9195, Discrimination (AUC)=0.989, Kappa=0.9003.

Environmental variables used: 1. Prec (mean), 2. Prec (max), 3. DEM, 4. Prec (A2) and 5. LST (min). Please see additional file 2 for abbreviations and definitions.

Copyright: Licensed to the Malaria Atlas Project (MAP; www.map.ox.ac.uk) under a Creative Commons Attribution 3.0 License (http://creativecommons.org/).

Citation: Sinka *et al.* (2010). The dominant *Anopheles* vectors of human malaria in Africa, Europe and the Middle East: occurrence data, distribution maps and bionomic précis. *Parasites & Vectors*, **3**:117.

Anopheles (*Cellia*) *melas* Theobald, 1903

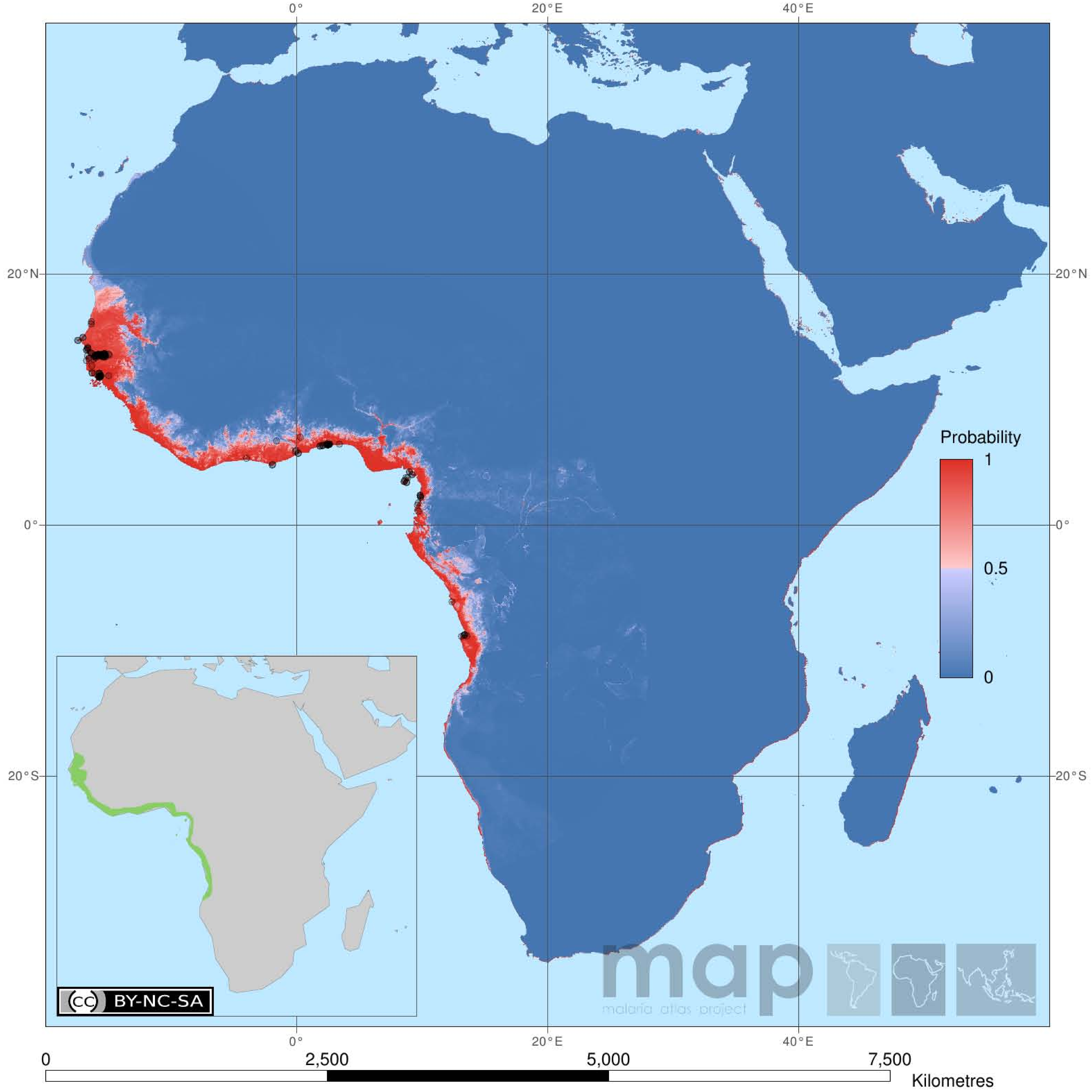

Mapping details: This map shows the predicted probability of occurrence of *An. melas* in Africa. The map was created with the Boosted Regression Trees (BRT) technique using 149 occurrence points, 500 pseudo occurrence points generated from a stratified random sample within the expert opinion range (see inset), balanced by 1,490 pseudo absence points sampled within a 1,500 km buffer outside the expert opinion range. The pseudo presence data were given half the weight of observed occurrence data. Predictions are not shown beyond the 1,500 km buffer. The black dots show 149 records of occurrence for *An. melas* as detailed in Hay *et al.* [1].

Map statistics: Deviance=0.1867, Correlation=0.9072, Discrimination (AUC)=0.9895, Kappa=0.8687.

Environmental variables used: 1. DEM, 2. LST (max), 3. Prec (P1), 4. Prec (max) and 5. LST (mean). Please see additional file 2 for abbreviations and definitions.

Copyright: Licensed to the Malaria Atlas Project (MAP; www.map.ox.ac.uk) under a Creative Commons Attribution 3.0 License (http://creativecommons.org/).

Citation: Sinka *et al.* (2010). The dominant *Anopheles* vectors of human malaria in Africa, Europe and the Middle East: occurrence data, distribution maps and bionomic précis. *Parasites & Vectors*, **3**:117.

Anopheles (*Cellia*) *merus* Dönitz, 1902

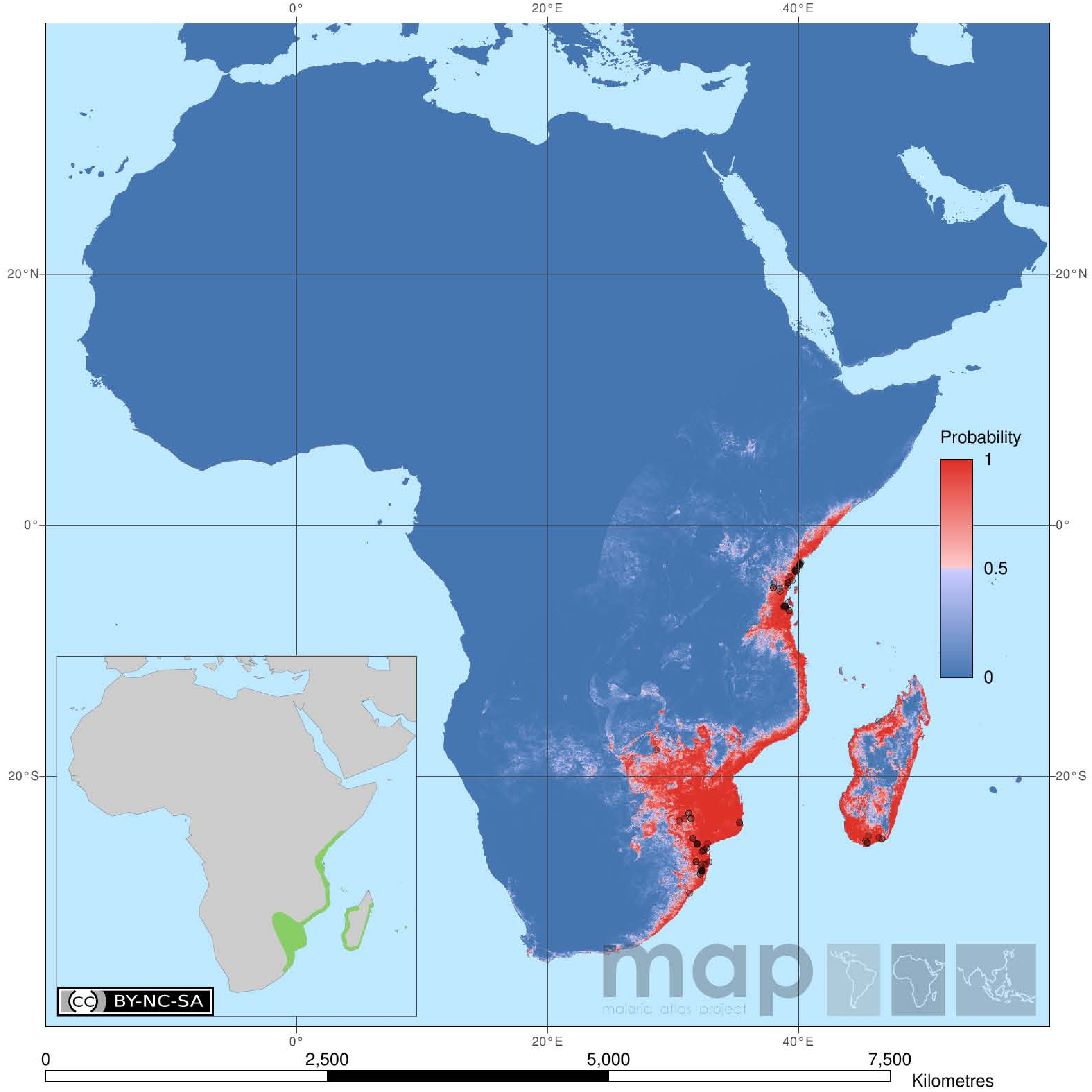

Mapping details: This map shows the predicted probability of occurrence of *An. merus* in Africa. The map was created with the Boosted Regression Trees (BRT) technique using 73 occurrence points, 500 pseudo occurrence points generated from a stratified random sample within the expert opinion range (see inset), balanced by 730 pseudo absence points sampled within a 1,500 km buffer outside the expert opinion range. The pseudo presence data were given half the weight of observed occurrence data. Predictions are not shown beyond the 1,500 km buffer. The black dots show 73 records of occurrence for *An. merus* as detailed in Hay *et al.* [1].

Map statistics: Deviance=0.2403, Correlation=0.9006, Discrimination (AUC)=0.983, Kappa=0.8763.

Environmental variables used: 1. DEM, 2. LST (A1), 3. Prec (P2), 4. MIR (P1) and 5. NDVI (P2). Please see additional file 2 for abbreviations and definitions.

Copyright: Licensed to the Malaria Atlas Project (MAP; www.map.ox.ac.uk) under a Creative Commons Attribution 3.0 License (http://creativecommons.org/).

Citation: Sinka *et al.* (2010). The dominant *Anopheles* vectors of human malaria in Africa, Europe and the Middle East: occurrence data, distribution maps and bionomic précis. *Parasites & Vectors*, **3**:117.

Anopheles (*Cellia*) *moucheti* Evans, 1925

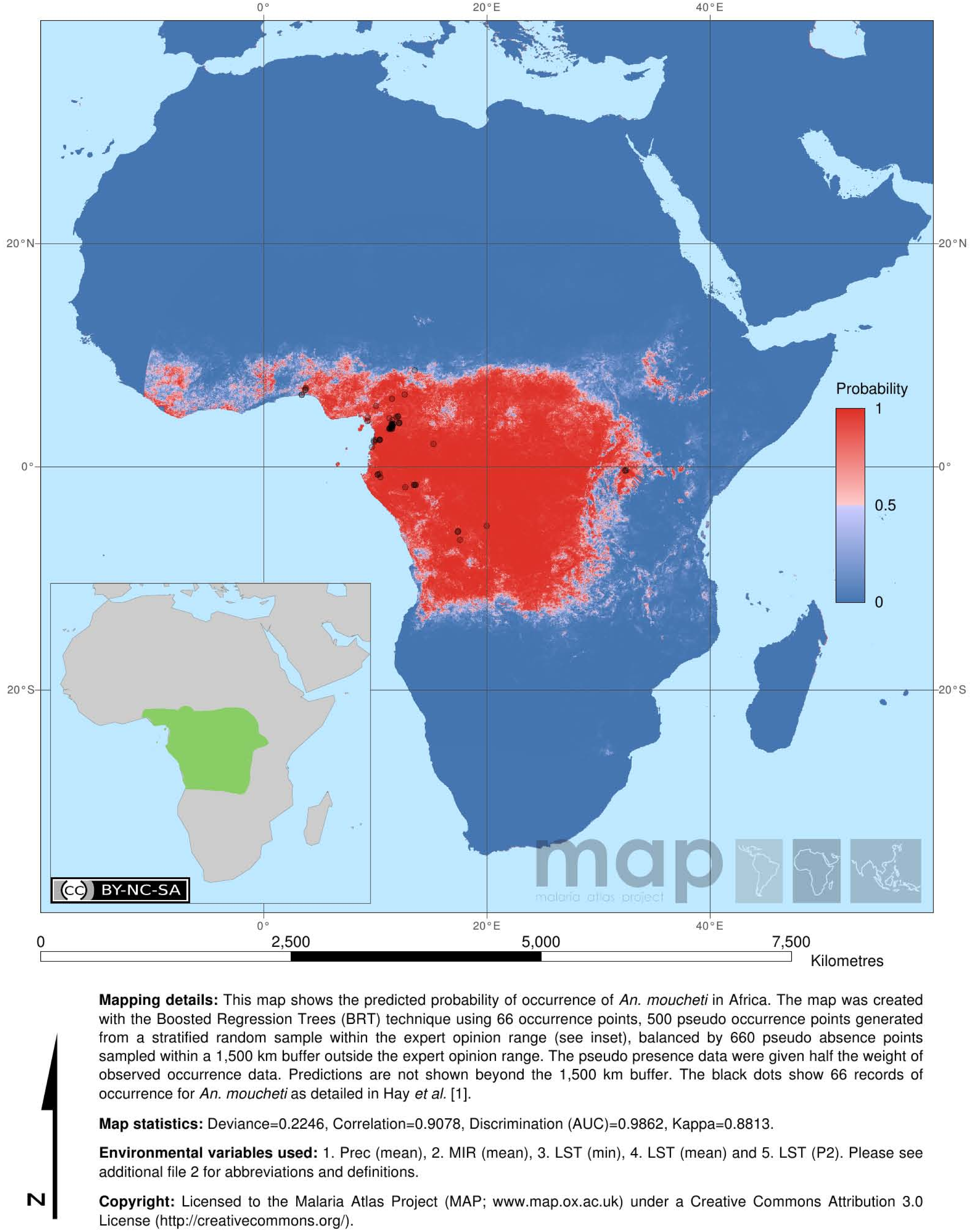

Mapping details: This map shows the predicted probability of occurrence of *An. moucheti* in Africa. The map was created with the Boosted Regression Trees (BRT) technique using 66 occurrence points, 500 pseudo occurrence points generated from a stratified random sample within the expert opinion range (see inset), balanced by 660 pseudo absence points sampled within a 1,500 km buffer outside the expert opinion range. The pseudo presence data were given half the weight of observed occurrence data. Predictions are not shown beyond the 1,500 km buffer. The black dots show 66 records of occurrence for *An. moucheti* as detailed in Hay *et al.* [1].

Map statistics: Deviance=0.2246, Correlation=0.9078, Discrimination (AUC)=0.9862, Kappa=0.8813.

Environmental variables used: 1. Prec (mean), 2. MIR (mean), 3. LST (min), 4. LST (mean) and 5. LST (P2). Please see additional file 2 for abbreviations and definitions.

Copyright: Licensed to the Malaria Atlas Project (MAP; www.map.ox.ac.uk) under a Creative Commons Attribution 3.0 License (http://creativecommons.org/).

Citation: Sinka *et al.* (2010). The dominant *Anopheles* vectors of human malaria in Africa, Europe and the Middle East: occurrence data, distribution maps and bionomic précis. *Parasites & Vectors*, **3**:117.

Anopheles (*Cellia*) *nili* species complex

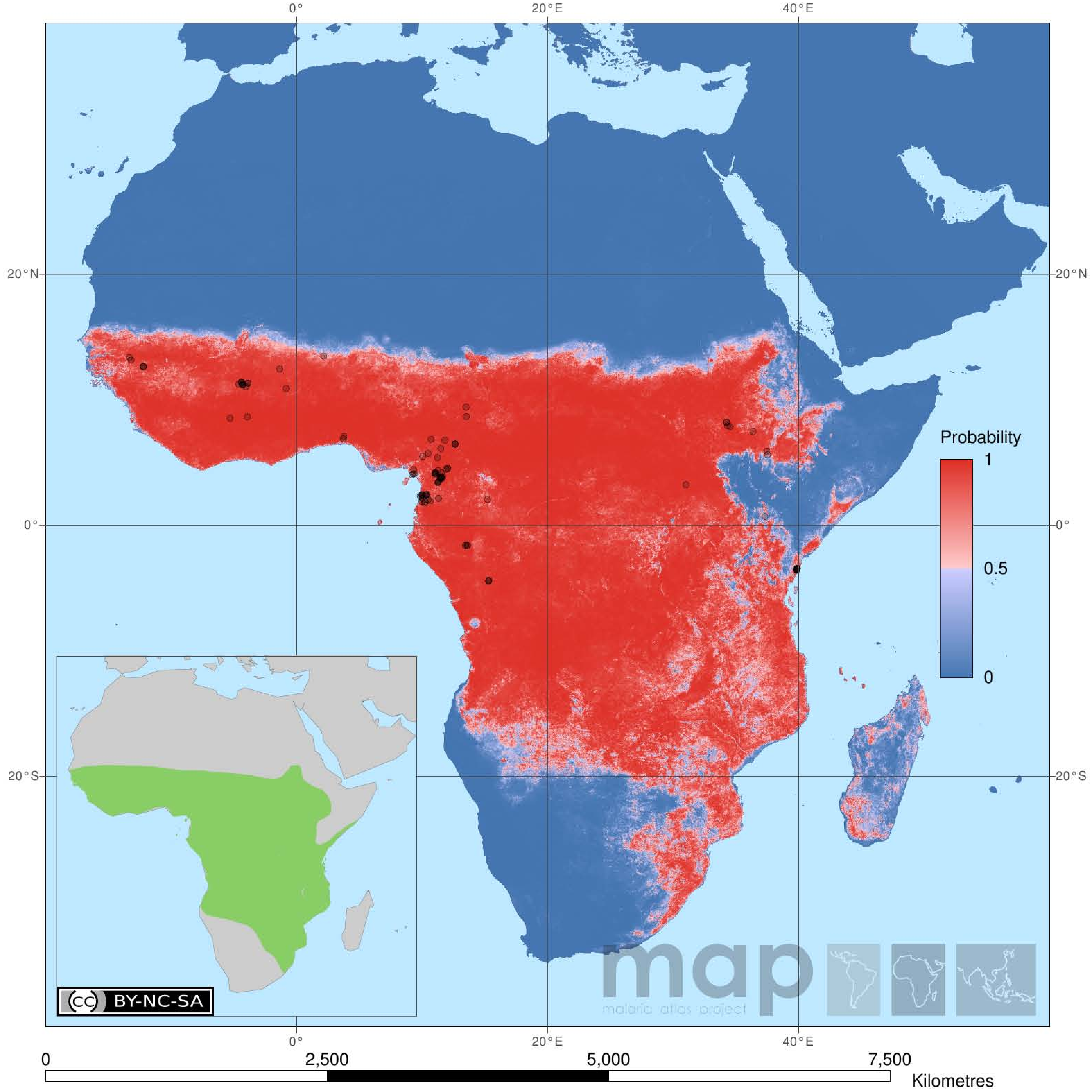

Mapping details: This map shows the predicted probability of occurrence of *An. nili* in Africa. The map was created with the Boosted Regression Trees (BRT) technique using 105 occurrence points, 500 pseudo occurrence points generated from a stratified random sample within the expert opinion range (see inset), balanced by 1,050 pseudo absence points sampled within a 1,500 km buffer outside the expert opinion range. The pseudo presence data were given half the weight of observed occurrence data. Predictions are not shown beyond the 1,500 km buffer. The black dots show 105 records of occurrence for *An. nili* as detailed in Hay *et al.* [1].

Map statistics: Deviance=0.2135, Correlation=0.9076, Discrimination (AUC)=0.9854, Kappa=0.8889.

Environmental variables used: 1. Prec (max), 2. GLOB (dry), 3. NDVI (max), 4. LST (min) and 5. NDVI (A2). Please see additional file 2 for abbreviations and definitions.

Copyright: Licensed to the Malaria Atlas Project (MAP; www.map.ox.ac.uk) under a Creative Commons Attribution 3.0 License (http://creativecommons.org/).

Citation: Sinka *et al.* (2010). The dominant *Anopheles* vectors of human malaria in Africa, Europe and the Middle East: occurrence data, distribution maps and bionomic précis. *Parasites & Vectors*, **3**:117.

Anopheles (*Anopheles*) *atroparvus* van Thiel, 1927

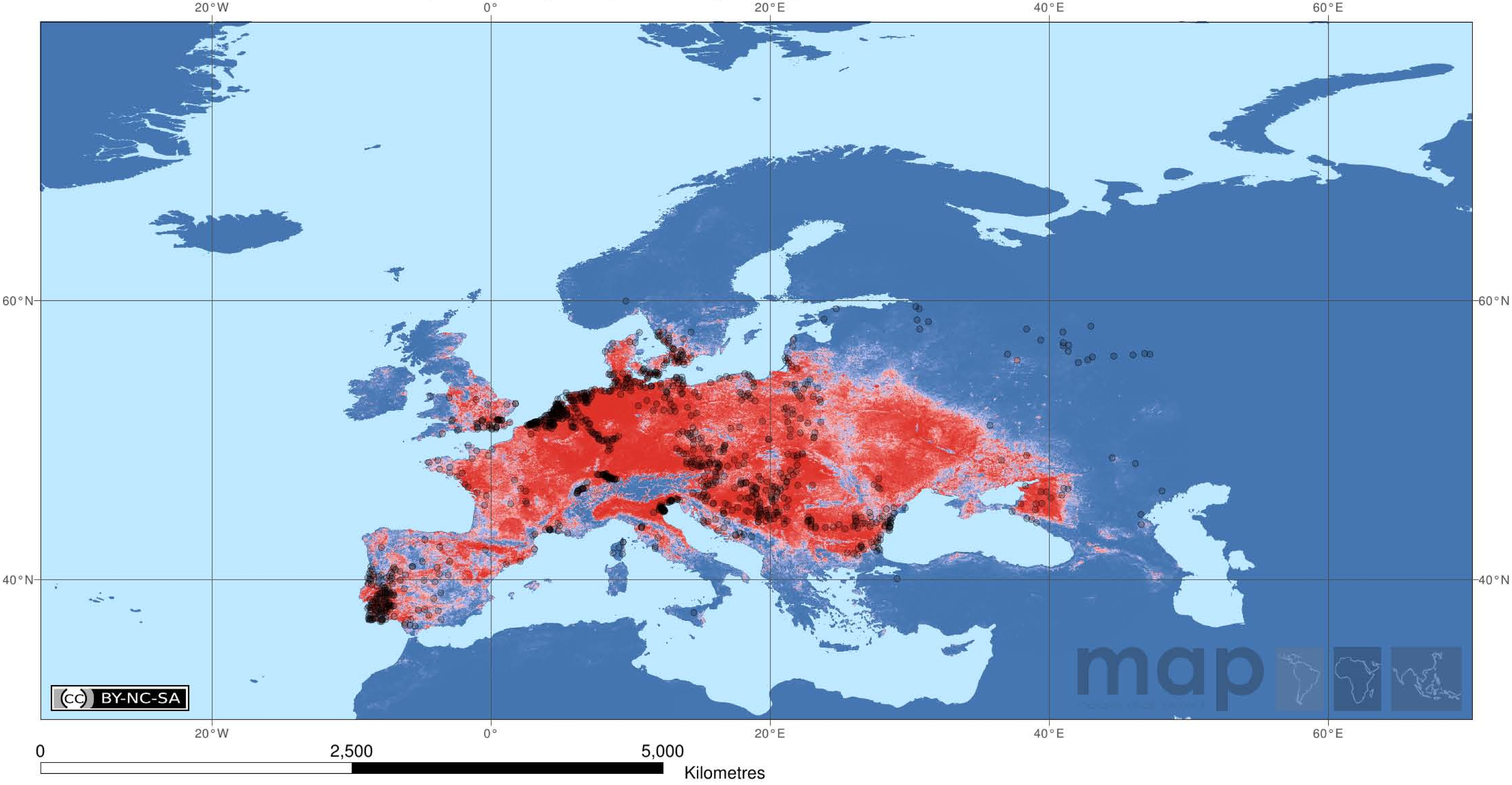

Mapping details: This map shows the predicted probability of occurrence of *An. atroparvus* in Europe and the Middle East. The map was created with the Boosted Regression Trees (BRT) technique using 1,044 occurrence points, 500 pseudo occurrence points generated from a stratified random sample within the expert opinion range (see inset), balanced by 10,440 pseudo absence points sampled within a 1,000 km buffer outside the expert opinion range. The pseudo presence data were given half the weight of observed occurrence data. Predictions are not shown beyond the 1,000 km buffer. The black dots show 1,044 records of occurrence for *An. atroparvus* as detailed in Hay *et al.* [1].

Map statistics: Deviance=0.1565, Correlation=0.8883, Discrimination (AUC)=0.9837, Kappa=0.8588.

Environmental variables used: 1. GLOB (190), 2. Prec (min), 3. EVI (mean), 4. EVI (P2) and 5. LST (P1). Please see additional file 2 for abbreviations and definitions.

Copyright: Licensed to the Malaria Atlas Project (MAP; www.map.ox.ac.uk) under a Creative Commons Attribution 3.0 License (http://creativecommons.org/).

Citation: Sinka *et al.* (2010). The dominant *Anopheles* vectors of human malaria in Africa, Europe and the Middle East: occurrence data, distribution maps and bionomic précis. *Parasites & Vectors*, **3**:117.

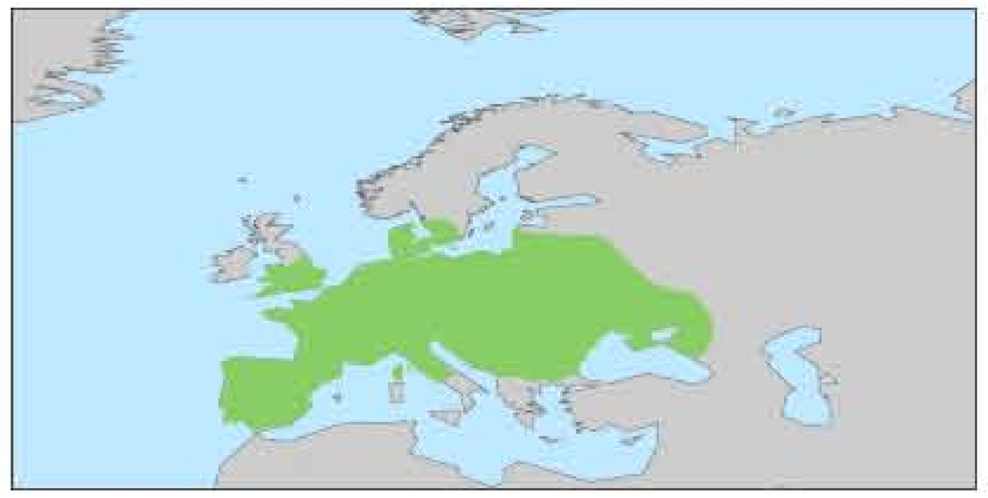

Probability

0 0.5 1

N

Anopheles (*Anopheles*) *labranchiae* Falleroni, 1926

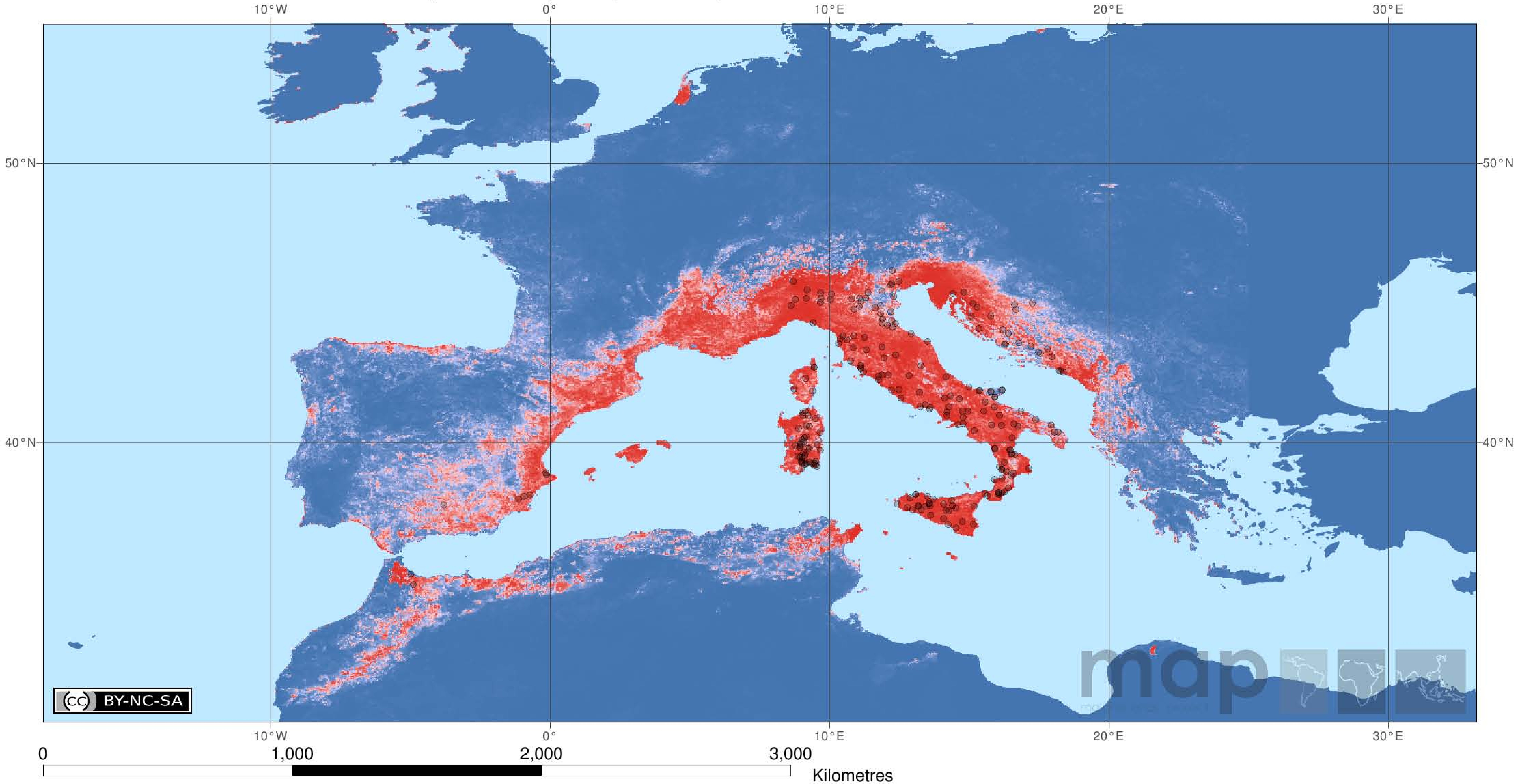

Mapping details: This map shows the predicted probability of occurrence of *An. labranchiae* in Africa and Europe. The map was created with the Boosted Regression Trees (BRT) technique using 234 occurrence points, 500 pseudo occurrence points generated from a stratified random sample within the expert opinion range (see inset), balanced by 2,340 pseudo absence points sampled within a 1,000 km buffer outside the expert opinion range. The pseudo presence data were given half the weight of observed occurrence data. Predictions are not shown beyond the 1,000 km buffer. The black dots show 234 records of occurrence for *An. labranchiae* as detailed in Hay *et al.* [1].

Map statistics: Deviance=0.2691, Correlation=0.8321, Discrimination (AUC)=0.9712, Kappa=0.781.

Environmental variables used: 1. Prec (P2), 2. GLOB (190), 3. Prec (A2), 4. NDVI (min) and 5. MIR (P1). Please see additional file 2 for abbreviations and definitions.

Copyright: Licensed to the Malaria Atlas Project (MAP; www.map.ox.ac.uk) under a Creative Commons Attribution 3.0 License (http://creativecommons.org/).

Citation: Sinka *et al.* (2010). The dominant *Anopheles* vectors of human malaria in Africa, Europe and the Middle East: occurrence data, distribution maps and bionomic précis. *Parasites & Vectors*, **3**:117.

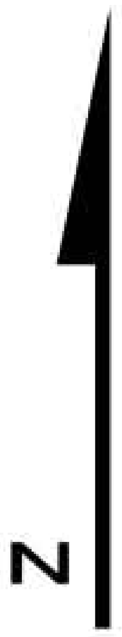

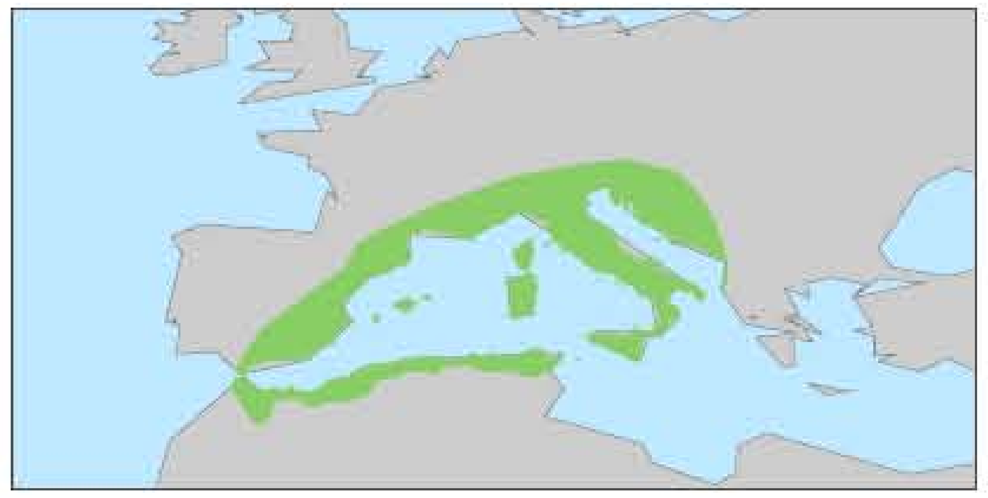

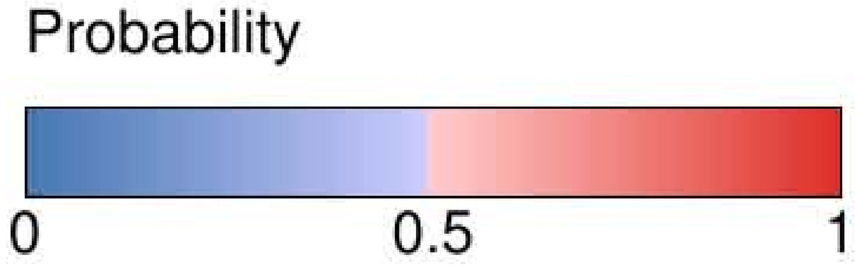

Anopheles (*Anopheles*) *messeae* Falleroni, 1926

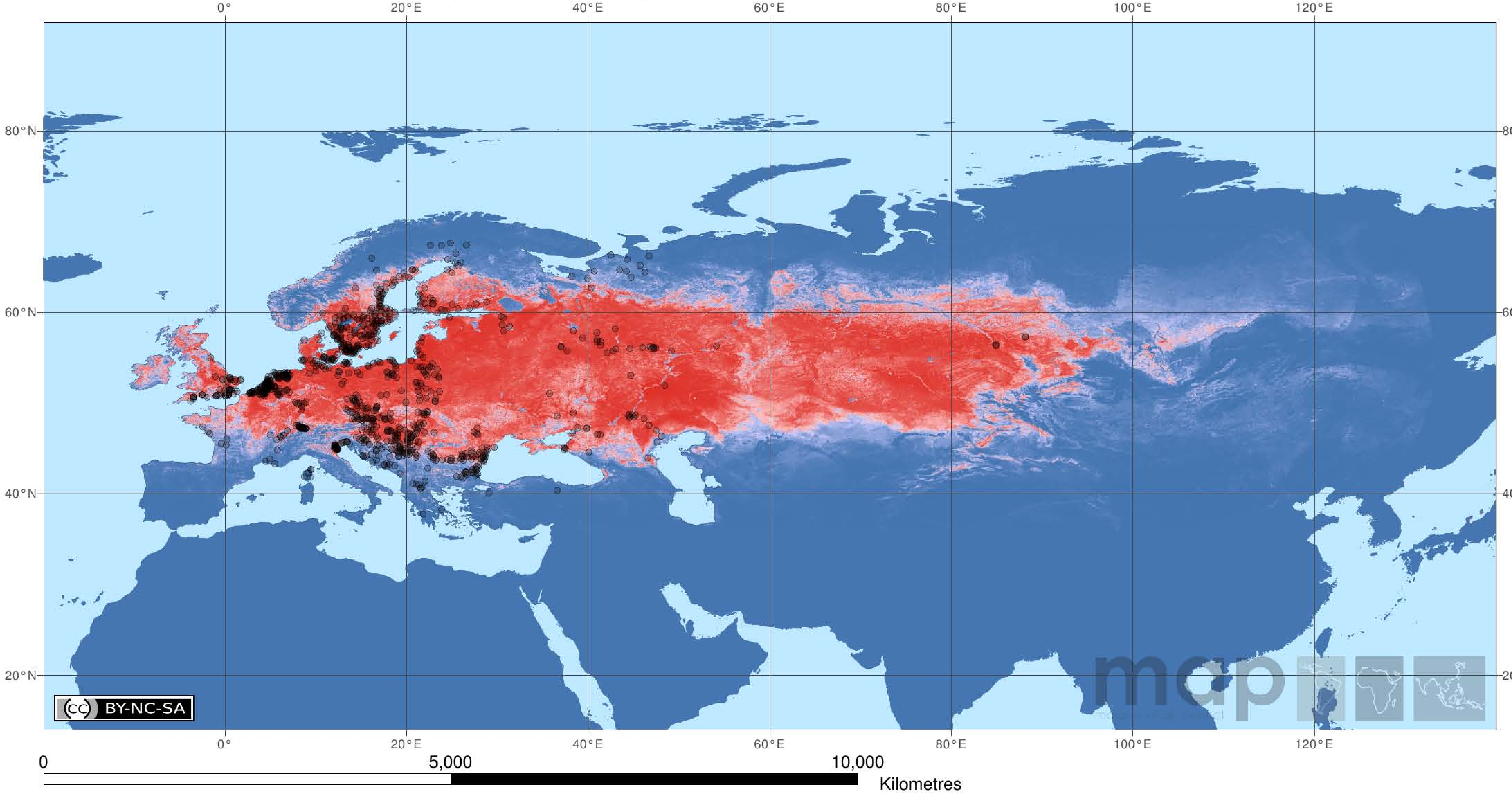

Mapping details: This map shows the predicted probability of occurrence of *An. messeae* in Europe and the Middle East. The map was created with the Boosted Regression Trees (BRT) technique using 903 occurrence points, 500 pseudo occurrence points generated from a stratified random sample within the expert opinion range (see inset), balanced by 9,030 pseudo absence points sampled within a 1,000 km buffer outside the expert opinion range. The pseudo presence data were given half the weight of observed occurrence data. Predictions are not shown beyond the 1,000 km buffer. The black dots show 903 records of occurrence for *An. messeae* as detailed in Hay *et al.* [1].

Map statistics: Deviance=0.208, Correlation=0.829, Discrimination (AUC)=0.9666, Kappa=0.7846.

Environmental variables used: 1. Prec (min), 2. Prec (P2), 3. GLOB (190), 4. DEM and 5. EVI (mean). Please see additional file 2 for abbreviations and definitions.

Copyright: Licensed to the Malaria Atlas Project (MAP; www.map.ox.ac.uk) under a Creative Commons Attribution 3.0 License (http://creativecommons.org/).

Citation: Sinka *et al.* (2010). The dominant *Anopheles* vectors of human malaria in Africa, Europe and the Middle East: occurrence data, distribution maps and bionomic précis. *Parasites & Vectors*, **3**:117.

N

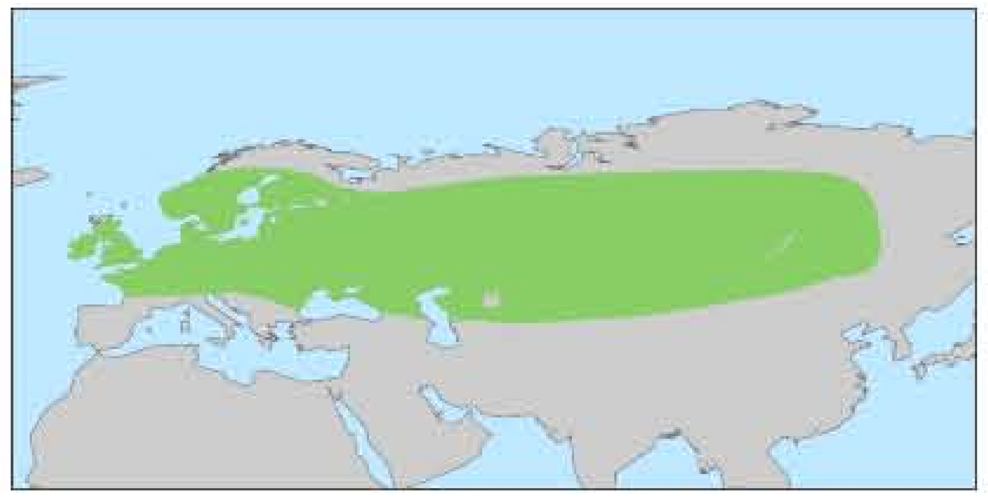

Probability

0 0.5 1

Anopheles (*Anopheles*) *sacharovi* Favre, 1903

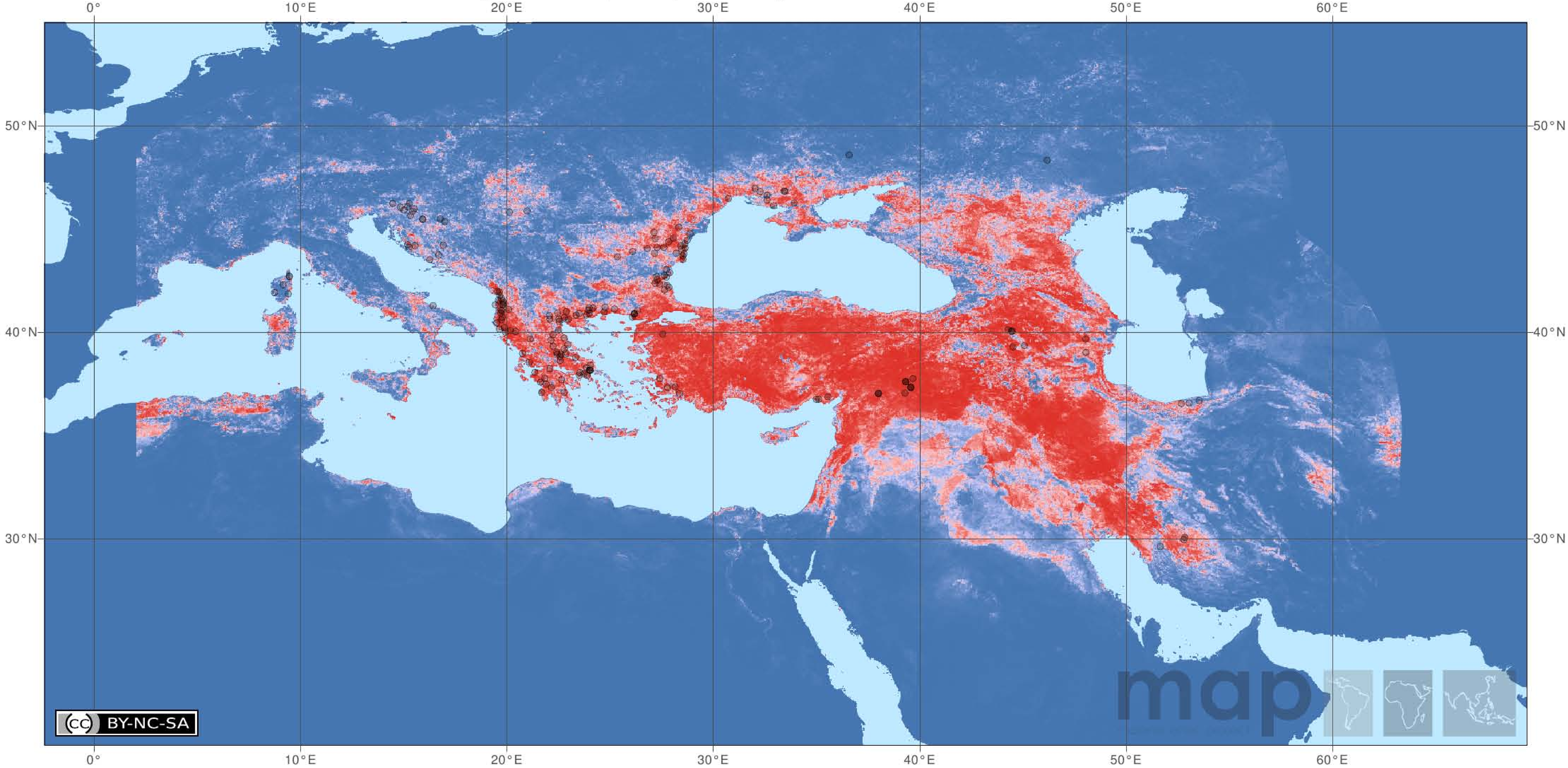

0 2,000 4,000
Kilometres

Mapping details: This map shows the predicted probability of occurrence of *An. sacharovi* in Europe and the Middle East. The map was created with the Boosted Regression Trees (BRT) technique using 183 occurrence points, 500 pseudo occurrence points generated from a stratified random sample within the expert opinion range (see inset), balanced by 1,830 pseudo absence points sampled within a 1,000 km buffer outside the expert opinion range. The pseudo presence data were given half the weight of observed occurrence data. Predictions are not shown beyond the 1,000 km buffer. The black dots show 183 records of occurrence for *An. sacharovi* as detailed in Hay *et al.* [1].

Map statistics: Deviance=0.3664, Correlation=0.7921, Discrimination (AUC)=0.9573, Kappa=0.726.

Environmental variables used: 1. Prec (A1), 2. MIR (P2), 3. DEM, 4. Prec (max) and 5. LST (min). Please see additional file 2 for abbreviations and definitions.

Copyright: Licensed to the Malaria Atlas Project (MAP; www.map.ox.ac.uk) under a Creative Commons Attribution 3.0 License (http://creativecommons.org/).

Citation: Sinka *et al.* (2010). The dominant *Anopheles* vectors of human malaria in Africa, Europe and the Middle East: occurrence data, distribution maps and bionomic précis. *Parasites & Vectors*, **3**:117.

N

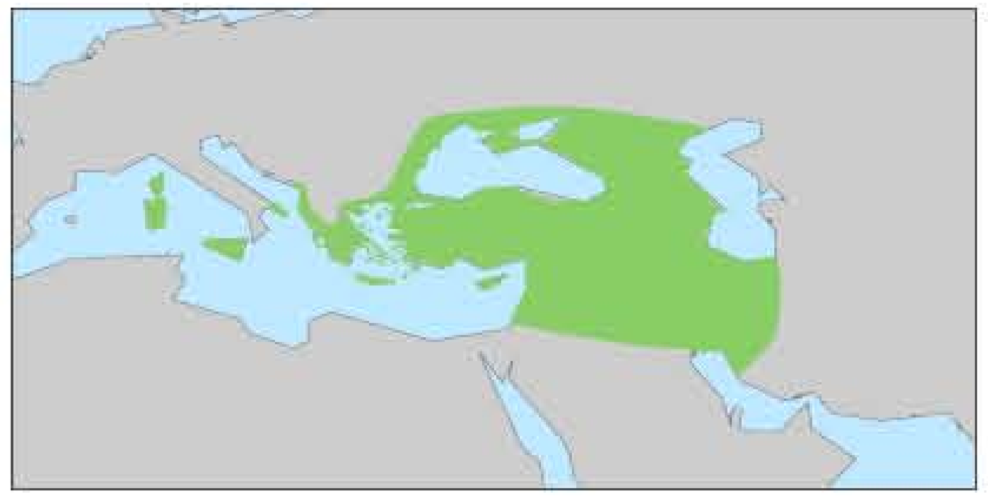

Probability

0 0.5 1

Anopheles (*Cellia*) *sergentii* species complex

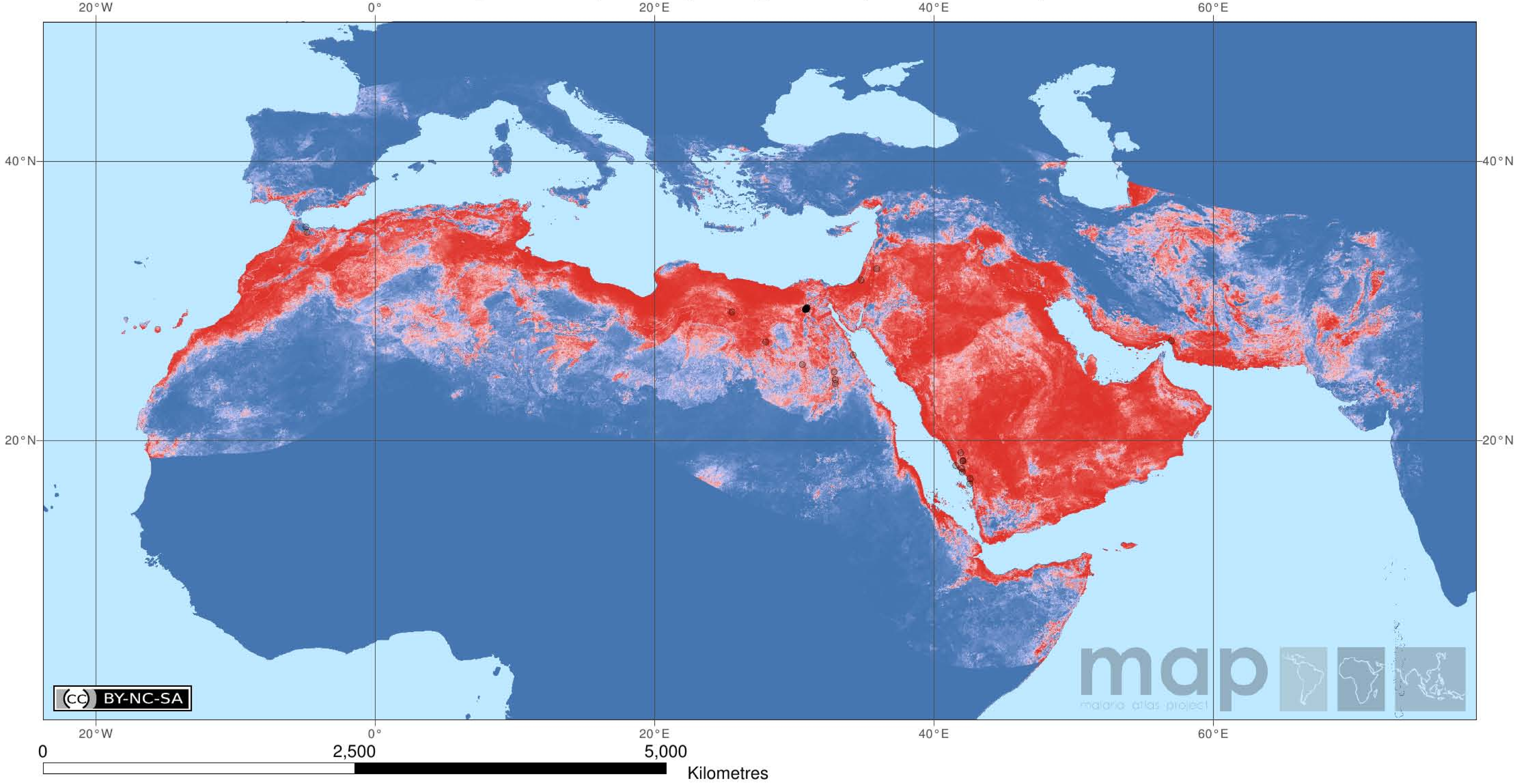

(cc) BY-NC-SA

Mapping details: This map shows the predicted probability of occurrence of *An. sergentii* in Africa and the Middle East. The map was created with the Boosted Regression Trees (BRT) technique using 35 occurrence points, 500 pseudo occurrence points generated from a stratified random sample within the expert opinion range (see inset), balanced by 350 pseudo absence points sampled within a 1,000 km buffer outside the expert opinion range. The pseudo presence data were given half the weight of observed occurrence data. Predictions are not shown beyond the 1,000 km buffer. The black dots show 35 records of occurrence for *An. sergentii* as detailed in Hay *et al.* [1].

Map statistics: Deviance=0.4372, Correlation=0.792, Discrimination (AUC)=0.9416, Kappa=0.7303.

Environmental variables used: 1. LST (min), 2. Prec (P1), 3. Prec (P2), 4. Prec (A2) and 5. DEM. Please see additional file 2 for abbreviations and definitions.

Copyright: Licensed to the Malaria Atlas Project (MAP; www.map.ox.ac.uk) under a Creative Commons Attribution 3.0 License (http://creativecommons.org/).

Citation: Sinka *et al.* (2010). The dominant *Anopheles* vectors of human malaria in Africa, Europe and the Middle East: occurrence data, distribution maps and bionomic précis. *Parasites & Vectors*, **3**:117.

N

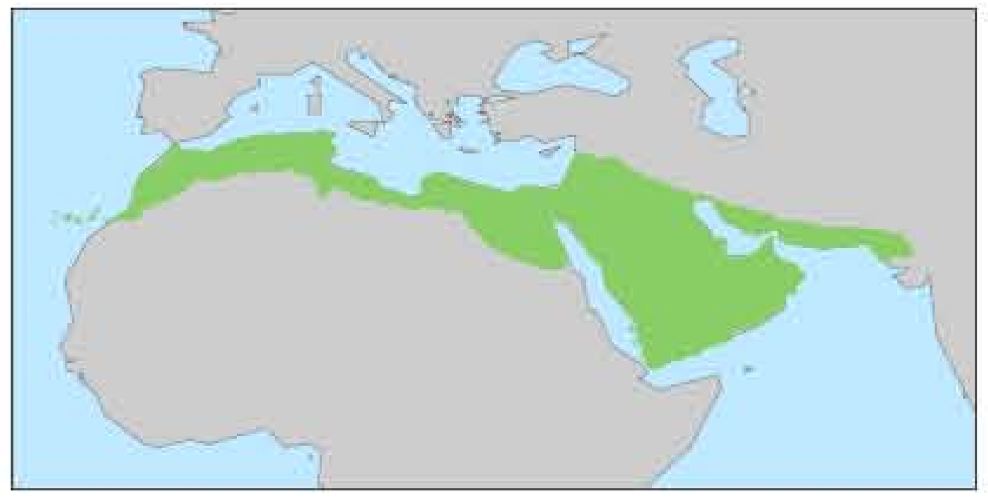

Probability

0 0.5 1

Anopheles (*Cellia*) *superpictus* Grassi, 1899

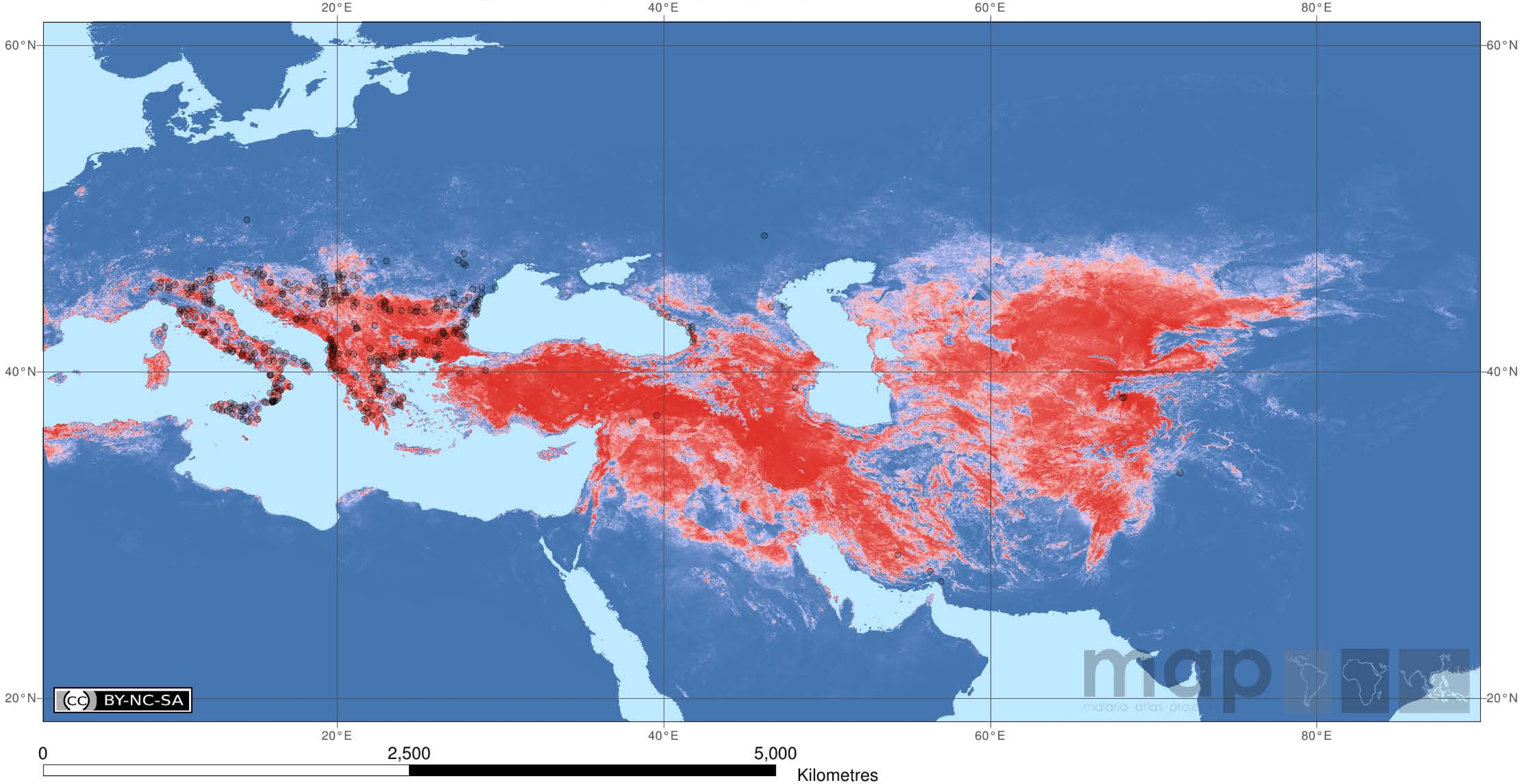

Mapping details: This map shows the predicted probability of occurrence of *An. superpictus* in Europe and the Middle East. The map was created with the Boosted Regression Trees (BRT) technique using 385 occurrence points, 500 pseudo occurrence points generated from a stratified random sample within the expert opinion range (see inset), balanced by 3,850 pseudo absence points sampled within a 1,000 km buffer outside the expert opinion range. The pseudo presence data were given half the weight of observed occurrence data. Predictions are not shown beyond the 1,000 km buffer. The black dots show 385 records of occurrence for *An. superpictus* as detailed in Hay *et al.* [1].

Map statistics: Deviance=0.2904, Correlation=0.7966, Discrimination (AUC)=0.9644, Kappa=0.7275.

Environmental variables used: 1. GLOB (190), 2. Prec (P1), 3. Prec (P2), 4. LST (min) and 5. NDVI (min). Please see additional file 2 for abbreviations and definitions.

Copyright: Licensed to the Malaria Atlas Project (MAP; www.map.ox.ac.uk) under a Creative Commons Attribution 3.0 License (http://creativecommons.org/).

Citation: Sinka *et al.* (2010). The dominant *Anopheles* vectors of human malaria in Africa, Europe and the Middle East: occurrence data, distribution maps and bionomic précis. *Parasites & Vectors*, **3**:117.

N

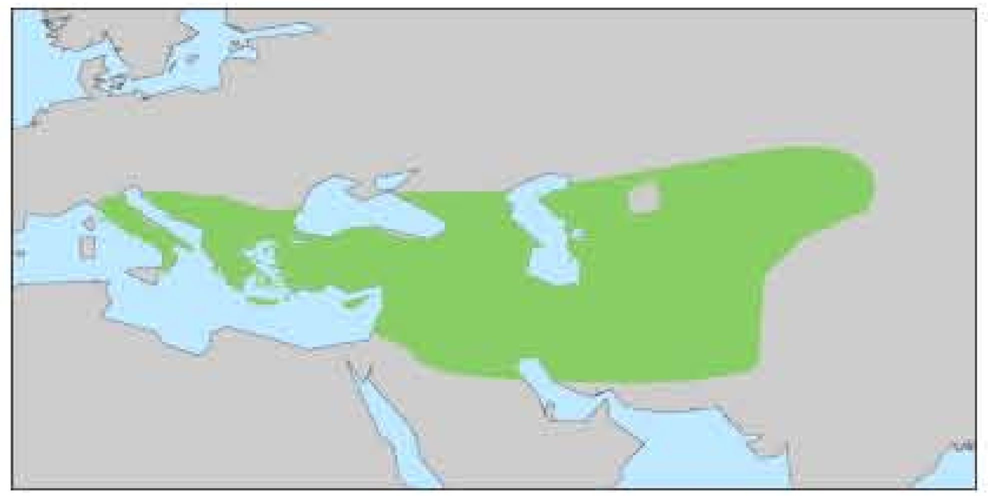

Probability

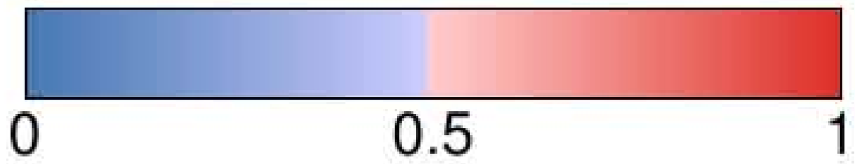

Supplement: Additional file 3 — Predictive species distribution maps for the seven DVS of Africa and the six DVS of the Europe and Middle Eastern region. [file 1756-3305-3-117-S3.PDF]
